# Environmental shaping of the bacterial and fungal community in infant bed dust and correlations with the airway microbiota

**DOI:** 10.1186/s40168-020-00895-w

**Published:** 2020-08-07

**Authors:** Shashank Gupta, Mathis H. Hjelmsø, Jenni Lehtimäki, Xuanji Li, Martin S. Mortensen, Jakob Russel, Urvish Trivedi, Morten A. Rasmussen, Jakob Stokholm, Hans Bisgaard, Søren J. Sørensen

**Affiliations:** 1 Section of Microbiology, Department of Biology, University of Copenhagen, Universitetsparken 15, bldg. 1, DK2100, Copenhagen, Denmark; 2 Copenhagen Prospective Studies on Asthma in Childhood, Faculty of Health Sciences, Copenhagen University Hospital Gentofte, University of Copenhagen, Gentofte, Denmark; 3 Host-Microbiota Interactions Laboratory, Wellcome Sanger Institute, Wellcome Genome Campus, Hinxton, UK; 4 Section of Chemometrics and Analytical Technologies, Department of Food Science, University of Copenhagen, Rolighedsvej 30, 1958 Frederiksberg C, Copenhagen, Denmark

**Keywords:** House dust, Bacterial microbiome, Fungal microbiome, Airway microbiome, Infant microbiome

## Abstract

**Background:**

From early life, children are exposed to a multitude of environmental exposures, which may be of crucial importance for healthy development. Here, the environmental microbiota may be of particular interest as it represents the interface between environmental factors and the child. As infants in modern societies spend a considerable amount of time indoors, we hypothesize that the indoor bed dust microbiota might be an important factor for the child and for the early colonization of the airway microbiome. To explore this hypothesis, we analyzed the influence of environmental exposures on 577 dust samples from the beds of infants together with 542 airway samples from the Copenhagen Prospective Studies on Asthma in Childhood_2010_ cohort.

**Results:**

Both bacterial and fungal community was profiled from the bed dust. Bacterial and fungal diversity in the bed dust was positively correlated with each other. Bacterial bed dust microbiota was influenced by multiple environmental factors, such as type of home (house or apartment), living environment (rural or urban), sex of siblings, and presence of pets (cat and/or dog), whereas fungal bed dust microbiota was majorly influenced by the type of home (house or apartment) and sampling season. We further observed minor correlation between bed dust and airway microbiota compositions among infants. We also analyzed the transfer of microbiota from bed dust to the airway, but we did not find evidence of transfer of individual taxa.

**Conclusions:**

Current study explores the influence of environmental factors on bed dust microbiota (both bacterial and fungal) and its correlation with airway microbiota (bacterial) in early life using high-throughput sequencing. Our findings demonstrate that bed dust microbiota is influenced by multiple environmental exposures and could represent an interface between environment and child.

Video Abstract

## Background

As societies become more modernized, people tend to spend an increasing amount of time indoors, especially within their homes [1]. Here, humans are exposed to a large number of microbes, which can have important implications for health and disease. With the advancement of sequencing technologies, it is now possible to study the indoor microbiome [2–4] and how microbes therein affect the inhabitants [5, 6]. Most studies to date have characterized the indoor microbiome in schools, homes, offices, hospitals, or kindergarten classrooms [5, 7–10]. In homes, studies were mostly done on floors, kitchen sinks, and bathrooms [5, 11]. However, very little is known about microbial communities present in beds with which humans have extended daily exposure [12].

Environmental factors such as pets, type of housing, and land use of the surrounding area have been associated with the microbiota of homes [2, 13, 14]. Many published studies have looked at the influence of pets in homes, but none have addressed a pet’s influence on the bed microbiome. Many pet owners share their bedroom space with their pets [15]; therefore, the pets may influence the bed dust composition, including health relevant taxa, as microbes can be airborne and get enriched in closed systems. Some studies reported that the exposure to pets (e.g., dogs and cats) decreases the risk of allergic diseases [16–18], where another has shown increased risk [19]. With the reasonably consistent findings across studies, exposure to pets, specifically dogs, remains a promising approach for identifying a prevention strategy for allergic diseases in early life.

Furthermore, especially among young infants, the bed dust is a highly relevant place of sampling for capturing the indoor dust microbiota, which can serve as a proxy for many environmental exposures and act as a seeding source for the microbiota colonizing the child. Early infancy characterizes a rapid developmental phase of the airway microbial colonization [20] but also with regard to immune function [21]. Elucidating the relationship between the indoor and infant airway microbiome in early life could be important in understanding human development, especially as the early life airway microbiota has been associated with later asthma development [22, 23]. The relationship between microbial exposures from surroundings and the composition of the infant airway microbiota is still poorly understood [24, 25].

In this study, we evaluate which environmental factors influence the bacterial and fungal composition of the infants’ beds at 6 months after birth. Additionally, we compare bed dust to the bacterial composition of the airways at age 3 months. All samples were collected in the Copenhagen Prospective Studies on Asthma in Childhood_2010_ (COPSAC_2010_) cohort [26]. Through a comprehensive analysis of the microbiomes in bed dust and early life airways, we aim to elucidate the interactions between the two and secondarily identify the external factors that affect the microbial interactions between bacterial and fungal microbiome. To our knowledge, this is the first study to provide detailed qualitative and quantitative descriptions of microbial taxa and diversity in bed dust.

## Results

### Characteristics of the cohort

In this study, we included 584 bed dust samples collected from the infants’ beds at 6 months after birth and 658 airway samples collected from the infants 3 months after birth. The demographic information about the study population in this study is summarized in Table S1.

### Sequencing results and quality control

In total, for the 584 dust samples and 70 controls (including negative and positive controls) obtained from the bed of the cohort children at age 6 months, we had 65,183,188 and 57,936,573 raw reads, from 16S ribosomal RNA gene (16S rRNA gene) (V3-V4 region) and internal transcribed spacer (ITS) amplicon (ITS2 region) sequencing including controls, respectively. After quality filtering and chimera detection, amplicon sequences were clustered into 79,347 and 24,474 amplicon sequence variants (ASVs) for 16S rRNA gene and ITS data. The coverage of our sequencing was assessed by rarefaction curves, showing a beginning plateau at 10,000 reads per sample (Fig. S1a and b). After removing the negative controls and the samples that did not reach a satisfactory read depth (minimum 3000 reads), we were left with 577 samples, representing 49,371 and 20,211 unique bacterial and fungal ASVs.

In the total 658 airway samples obtained from the cohort from children at age three, 34,319,874 raw reads passed quality filtering. After quality filtering and chimera detection, amplicon sequences were clustered into 3,692 ASVs for 16S rRNA gene (V4 region) data. We removed airway samples without a matching bed dust sample from the downstream analysis, ending up with 542 samples that contained a total of 2,272 ASVs.

### Microbial community composition in bed dust

**Fig. 1:**
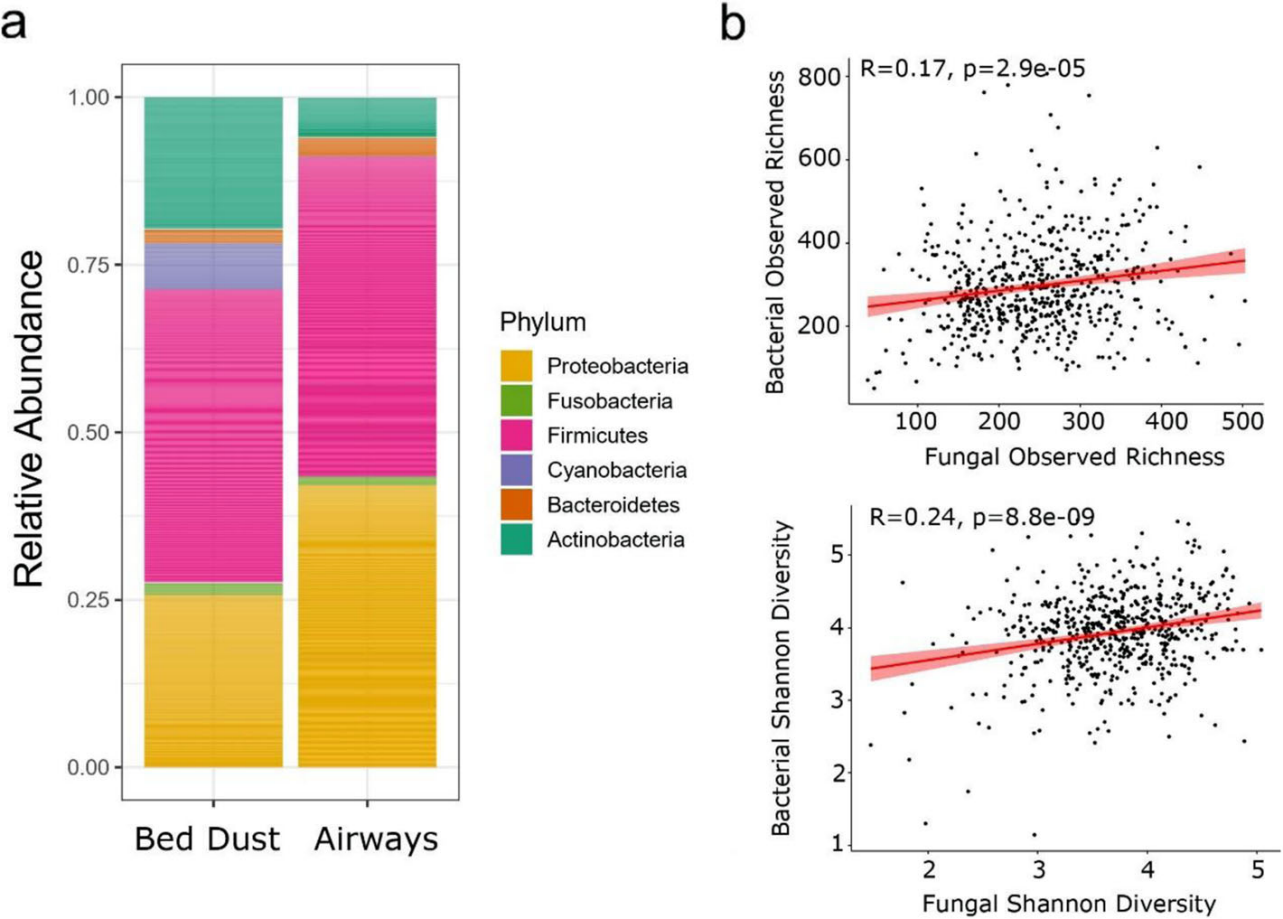
**a** Relative abundance of bacterial phyla in bed dust and airway samples. Phyla with a mean abundance of at least 1% abundance across bed dust samples are represented in colors. **b** Associations between fungal and bacterial alpha diversity (observed richness and Shannon diversity index values) for a given sample. The shaded gray region represents 95% confidence intervals. Linear regression analysis: *p* = 2.9e−05, *R* = 0.17 for observed richness and *p* = 8.8e−09, *R* = 0.25 for Shannon diversity

A total of 930 bacterial genera from 31 phyla were detected in the beds of 6-month-old infants. The most abundant phyla were Firmicutes (43.05%), Proteobacteria (25.69%), Actinobacteria (19.27%), Cyanobacteria (6.89%), Bacteroidetes (2.17%), and Fusobacteria (1.85%) (Fig. 1a, Fig. S3a, Table S2). The remaining 25 phyla combined represented 1% of the relative abundance. Taxonomic identification at the class, family, and genus levels (Fig. S3b, c, and d, respectively) revealed that most of the Firmicutes belonged to the class Bacilli, with varying amounts of the families Streptococcaceae and Staphylococcaceae. Among these, the most abundant genera were *Streptococcus* (23.6%), *Staphylococcus* (12.43%), *Rothia* (6.17%), *Haemophilus* (4.15%), *Paracoccus* (4.12%), and *Corynebacterium* (4.09%) (Table S3).

A total of 102 fungal genera from 6 phyla were detected. The most abundant phyla were Ascomycota (82.47%) and Basidiomycota (6.60%) (Fig. S4a, Table S4). The remaining four phyla represented less than 1% of the overall abundance with an additional 10.53% of the sequences not classified at the phylum level (Fig. S4b, Table S4). A similar trend was observed at the family level (Fig. S4c) and class level (Table S5). The most abundant genera were *Spegazzinia* (9.61%), *Aureobasidium* (5.34%), *Sphaerellopsis* (4.99%), *Curvularia* (4.83%), *Saccharomyces* (4.50%), and *Penicillium* (3.55%) (Fig. S4d, Table S6), with 44.1% of reads being unclassified Ascomycota.

### Correlations between the bacteria and fungi in bed dust

We explored the relationship between the bacterial and fungal members in the bed dust microbiota of the children at 6 months. Bacterial and fungal alpha diversity values were positively correlated (*r*_observed_ = 0.17, *r*_shannon_ = 0.24), when assessed by linear regression (*p*_observed_ = 2.9e−05, *p*_shannon_ = 8.8e−09) (Fig. 1b). We further looked at the correlation both within and between the fungal and bacterial microbiomes for the genera present in at least 30% of the samples (*n* = 173). For fungal-fungal correlation, most of the significant correlations (*p* < 0.01) were positive, while correlations between *Saccharomyces* with *Spegazzinia* (Spearman correlation coefficient, *r* = − 0.15), *Curvularia* (Spearman correlation coefficient, *r* = − 0.23), *Sphaerellopsis* (Spearman correlation coefficient, *r* = − 0.19), and *Neophaeosphaeria* (Spearman correlation coefficient, *r* = − 0.13) were negative (Fig. S5). The strongest positive correlation occurred between *Spegazzinia* and *Curvularia* (Spearman correlation coefficient, *r* = 0.54), while *Curvularia* and *Saccharomyces* (Spearman correlation coefficient, *r* = − 0.23) exhibited the strongest negative correlation.

A higher number of significant correlations were found for bacteria-bacteria correlation compared to fungal-fungal correlation. The strength of negative correlation is very low in bacteria-bacteria correlation (Spearman correlation coefficient, (*r*) range from − 0.12 to − 0.28). For example, the genera *Sphingomonas* was significantly correlated (*p* < 0.01) with more than 30 genera; most correlations were positive (Spearman correlation coefficient, (*r*) range from 0.15 to 0.72) and showed negative correlations with 10 genera (Spearman correlation coefficient, (*r*) range from − 0.12 to − 0.16). *Peptoniphilus* genera showed the strongest positive correlation with *Finegoldia* (Spearman correlation coefficient, *r* = 0.75). We also observed many clinically relevant genera that were significantly correlating with many other genera, for example, Moraxella showed strongest positive correlation with *Abiotrophia* genus (Spearman correlation coefficient, *r* = 0.32), whereas Staphylococcus showed the strongest positive correlation with *Corynebacterium* (Spearman correlation coefficient, *r* = 0.6) and strongest negative correlation with Streptococcus (Spearman correlation coefficient, *r* = − 0.27). On the other hand, *Streptococcus* shows significantly positive correlation with *Gemella* (Spearman correlation coefficient, *r* = 0.64) (Fig. S6).

**Fig. 2:**
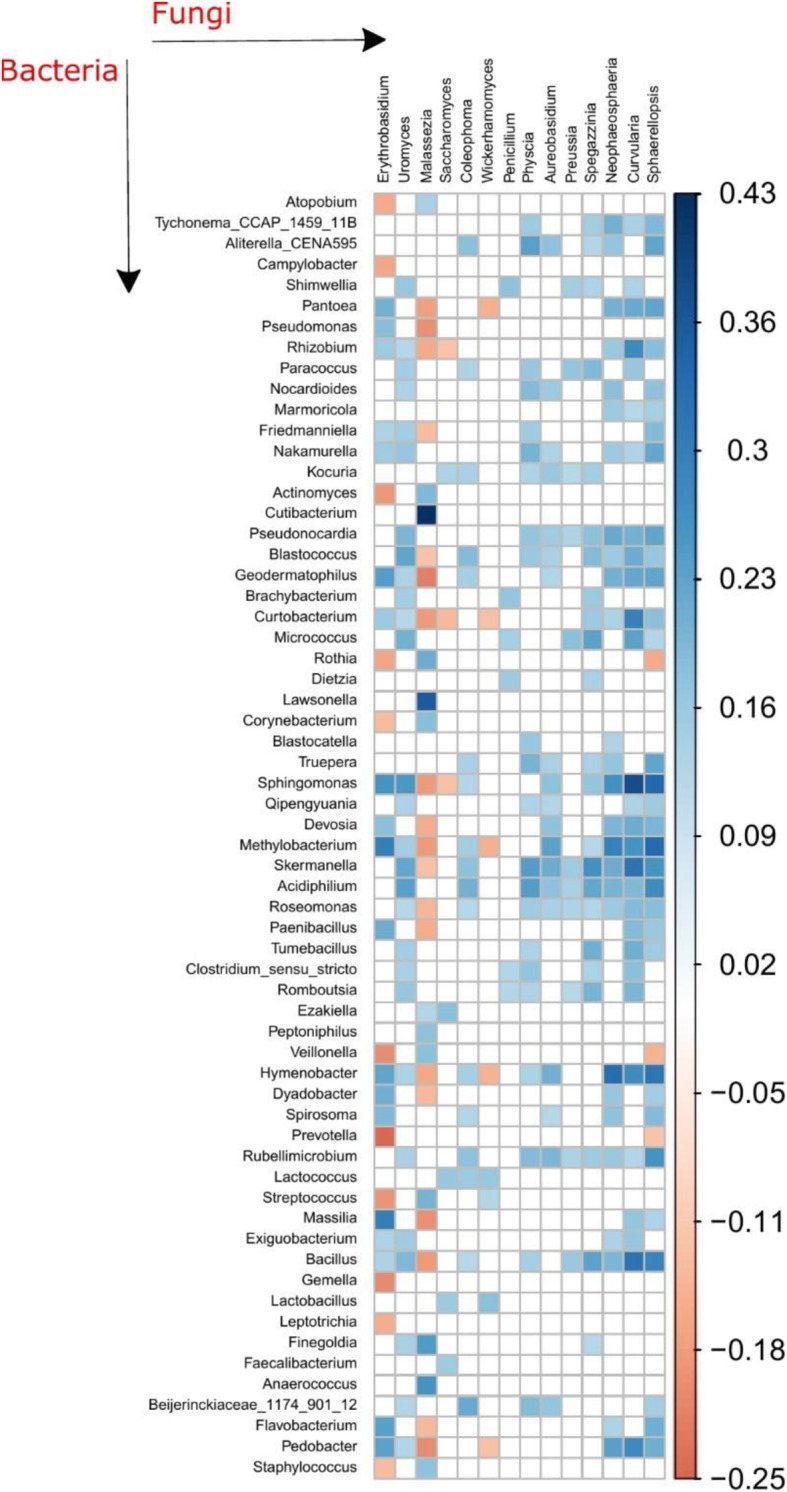
Spearman correlation by genus abundance. Only significant values (*p* < 0.05 after FDR adjustment) are shown. Correlation inferred for the bed dust microbiome based on bacteria and fungi combined. Orange and blue represents significant negative correlations and positive correlations. Darker color represents stronger correlations

We next assessed the correlations between the fungal and bacterial microbiota at the genus level. Comparing the relative abundances, both positive and negative correlations existed between fungal and bacterial taxa (Fig. 2). *Cutibacterium* and *Malassezia* showed the strongest inter-domain positive correlation (Spearman correlation coefficient, *r* = 0.43), whereas *Prevotella* with *Erythrobasidium* (Spearman correlation coefficient, *r* = − 0.25) exhibited the strongest negative correlation.

### Environmental factors shaping the bed dust microbiome

The effect of environmental factors on the bed dust microbiome, i.e., income level, type of home, type of living environment, pets, season of dust sample collection, race, and number of male or female siblings, was evaluated (Table 1). The bacterial richness in the bed dust was significantly affected by the type of home (house or apartment, median richness 294 and 269, respectively), type of living environment (rural or urban, median richness 298 and 267, respectively), and pets (cat and dog vs no pets, median richness 346 and 273, respectively) (Wilcoxon test, adjusted *p* = 0.0089, adjusted *p* = 0.0004, and adjusted *p* = 0.00045, respectively). We investigated the bacterial richness in the apartment and house locating either in the rural and urban areas. Apartments in rural areas showed significantly higher bacterial richness compared to apartments in urban areas (apartments in rural vs urban, median richness 302 vs 263) (Wilcoxon test, adjusted *p* = 0.024) while houses had no significant differences between rural and urban areas (Fig. S7a). On the other hand, bacterial richness between houses and apartments in rural and urban areas was not significantly different (Fig. S7b).

**Table 1:** The effects of environmental factors on alpha and beta diversity on the bed dust microbiome

Category	Variable	Overall *n* (%)	Bacterial Alpha diversity (median richness)	Bacterial Alpha diversity^**^**^ Adjusted *p* value	Bacterial Beta diversity^**#**^ *R*^2^/*p* value	Fungal Alpha diversity (median richness)	Fungal Alpha diversity^**^**^ Adjusted *p* value	Fungal Beta diversity^**#**^ *R*^2^/*p* value
	*n*	577						
Sex	Male	301 (52.2)						
Income level*	Low	52 (9)	308	0.46	0.004/0.132	228	0.51	0.003/0.417
	Medium	302 (52.3)	285			246		
	High	222 (38.5)	274			246		
Type of home	House^↑^	316 (54.8)	294	**0.0089**	0.003/0.076	251	**0.0016**	0.003/0.139
	Apartment	231 (40)	269			230		
Type of living environment	Rural^↑^	251 (43.5)	298	**0.0004**	**0.008/0.001**	246	0.14	**0.014/0.001**
	Urban	295 (51.1)	266			242		
Pets	Cat	87 (15.1)	292	**0.0039**	**0.007/0.005**	255	0.52	0.004/0.468
	Dog	69 (11.9)	294			252		
	Both^↑^	32 (5.5)	346			247		
Season of dust sample collection	Winter (December, January, February)	140 (24.5)	272	0.97	**0.01/0.001**	233	**1.3e**−**05**	**0.105/0.001**
	Spring^↑^ (March, April, May)	115 (20.1)	288			266		
	Summer^↑^ (June, July, August)	149 (26)	283			262		
	Autumn (September, October, November)	168 (29.4)	277			229		
Race	Caucasian	552 (95.7)	281	0.12	0.002/0.054	243	0.9	0.001/0.442
Siblings	No	132 (22.87)	265	**0.03**	**0.003/0.026**	225	**0.0023**	0.0023/0.329
	Yes^↑^	329 (57.02)	298			255		
Siblings	No	132 (22.87)	265	**0.049**	**0.009/0.003**	225	**0.0093**	0.011/0.053
	Male only	140 (24.26)	311 ^↑^			258 ^↑^		
	Female only	121 (20.97)	284			251 ^↑^		
	Both male and female	68 (11.78)	290			273 ^↑^		
Number of male siblings	None	132 (22.87)	265	**0.0015**	0.007/0.33	225	**0.0062**	0.0003/0.99
	One	114 (19.75)	308			255		
	Two or more^↑^	26 (4.5)	330			267		
Number of female siblings	None	132 (22.87)	265	0.29	0.008/0.33	225	0.11	0.003/0.95
	One	97 (16.81)	274			248		
	Two or more	24 (4.16)	321			257		

^^^Alpha diversity were calculated based on observed richness and significance were calculated using the Wilcoxon test (for two groups) and Kruskal-Wallis test (for three or more groups), FDR corrected

^#^Effects were quantified with *R*^2^, and *p* values, as determined by PERMANOVA on weighted UniFrac distances. Significant adjusted *p* values (*p* < 0.05) are shown in bold

^↑^The significant increase in alpha diversity of bacterial and/or fungal microbiome

*Income level is categorize into low (< €50,000/year), medium (€50,000–€110,000/year), and high (> €110,000/year)

The fungal richness was significantly affected by the type of home (house or apartment, median richness 251 and 230, respectively) and the season of sampling (median richness for summer 262, winter 233, spring 266, and autumn 229) (Kruskal-Wallis test, adjusted *p* = 1.3e−05). The samples collected in spring and summer showed a higher fungal diversity. We further investigated the fungal richness into the apartment and house present in the rural and urban areas, and we did not observe any significant differences (Fig. S8a). On the other hand, fungal richness between house and apartment in urban areas was significantly different (house vs apartment in urban areas, median richness 255 vs 228) (Wilcoxon test, *p* = 0.003) (Fig. S8b), whereas the houses and apartments present in rural areas were not.

Moreover, siblings correlated with an increased bacterial (no vs yes, median richness 265 vs 298) (Wilcoxon test, adjusted *p* = 0.03) and fungal richness (no vs yes, median richness 226 vs 255) (Wilcoxon test, adjusted *p* = 0.0023). We further observed that homes that had only male siblings showed significantly higher bacterial richness compared to homes that had no siblings (median richness 311 vs 265) (Wilcoxon test, adjusted *p* = 0.0079). However, homes that had only female siblings did not show any significant difference (median richness 265 vs 284) (Wilcoxon test, adjusted *p* = 0.4). Moreover, fungal richness correlated with male and/or female siblings (median richness for no siblings 225, male only 258, female only 251, both male and female 273) (Kruskal-Wallis test, adjusted *p* = 0.0093). We further observed that number of male siblings correlated with an increase in bacterial (none vs two or more, median richness 265 vs 330) (Wilcoxon test, adjusted *p* = 0.0004) and fungal richness (none vs two or more, median richness 225 vs 267) (Wilcoxon test, adjusted *p* = 0.0047). On the other hand, we did not observe any significant changes in bacterial (none vs two or more, median richness 265 vs 321) (Wilcoxon test, adjusted *p* = 0.1) or fungal richness (none vs two or more, median richness 225 vs 257) (Wilcoxon test, adjusted *p* = 0.073) in relation to increase in female siblings.

The bacterial microbial community (beta diversity) was significantly affected by the type of living environment, pets, the season of dust samples collection, and presence of siblings (PERMANOVA for weighted Unifrac, *p* = 0.001, *R*^2^ = 0.008; *p* = 0.005, *R*^2^ = 0.007; *p* = 0.001, *R*^2^ = 0.01; and *p* = 0.026, *R*^2^ = 0.003, respectively). With the exception of pets and siblings, this was also the case for the fungal microbial community (PERMANOVA for weighted Unifrac, *p* = 0.001, *R*^2^ = 0.014 and *p* = 0.001, *R*^2^ = 0.105, respectively) (Table 1). We next investigated the interaction between all the significant factors and look for the marginal effects. We performed PERMANOVA for weighted Unifrac distance; environmental factors namely season had the largest interaction with bacterial and fungal bed dust microbiome composition (*p* = 0.001, *R*^2^ = 0.021; *p* = 0.001, *R*^2^ = 0.084, respectively) (Table S15).

### Influence of pets on bed dust microbiome

**Fig. 3:**
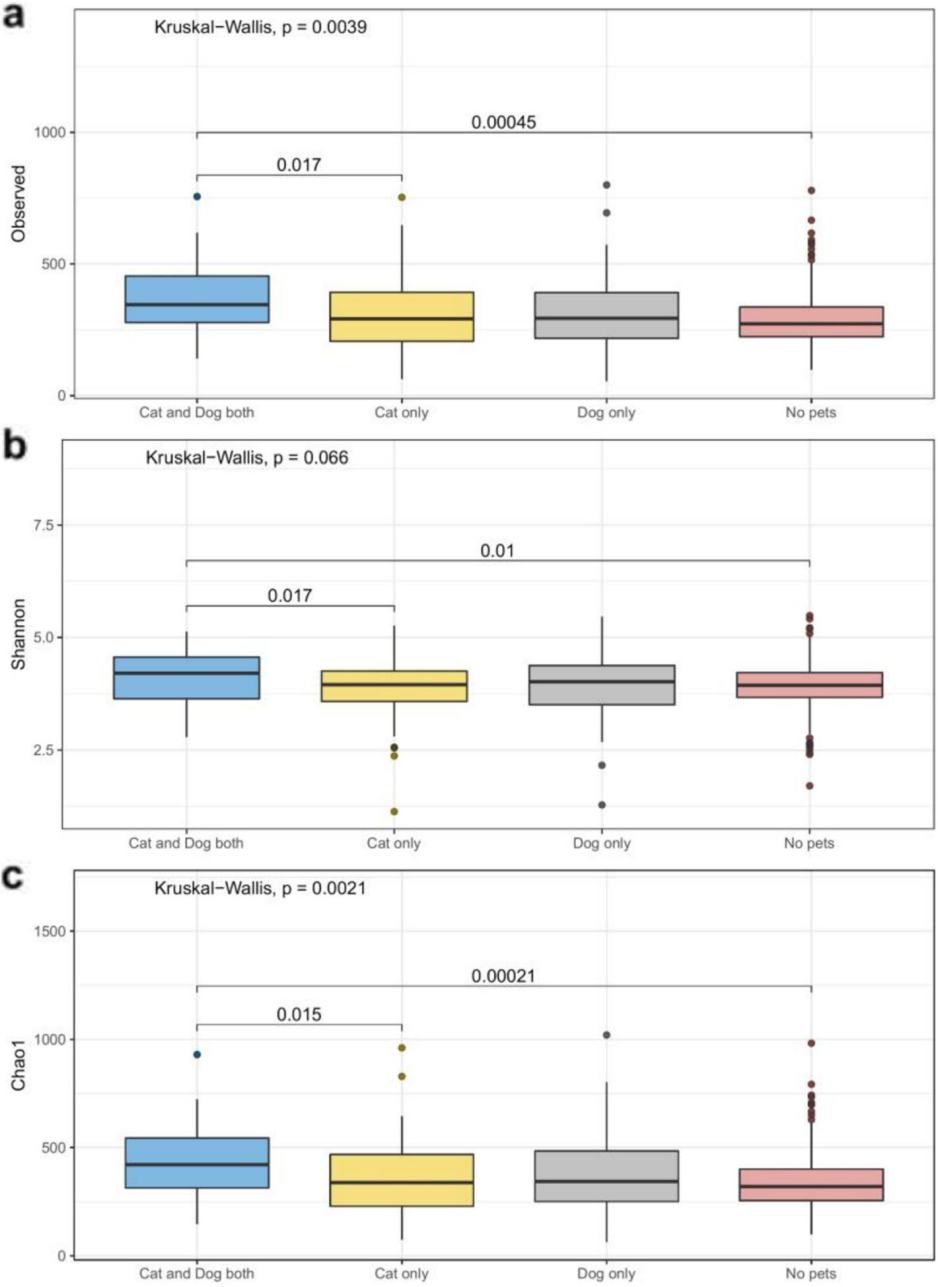
Box plots of the three diversity metrics for bacteria [**a** observed, **b** Shannon diversity, and **c** Chao1 diversity] with homes categorized according to pet ownership. Alpha diversity was tested using the Kruskal-Wallis test, and Benjamini-Hochberg FDR method was used for *p* value correction. After the global test was significant, a Wilcoxon test was performed to determine which group of the independent variable differs from each other group

Various environmental factors influenced the bed dust microbiome composition (Table 1), and we performed in-depth evaluations of the effects of pets. Among the families, 87/577 (15.1%) had cat only, 69/577 (11.9%) had dogs only, and 32/577 (5.5%) had both cat and dog. We observed that the bacterial alpha diversity were not significantly associated with the presence of either a cat or a dog only, but significantly higher in homes with both cat and dog (Kruskal-Wallis test, *p*_observed_ = 0.0039, *p*_shannon_ = 0.066, *p*_chao1_ = 0.0021) (Fig. 3), whereas fungal alpha diversity was not influenced by the pet ownerships (Kruskal-Wallis test, *p*_observed_ = 0.52, *p*_shannon_ = 0.92, *p*_chao1_ = 0.46) (Fig. S9).

**Fig. 4:**
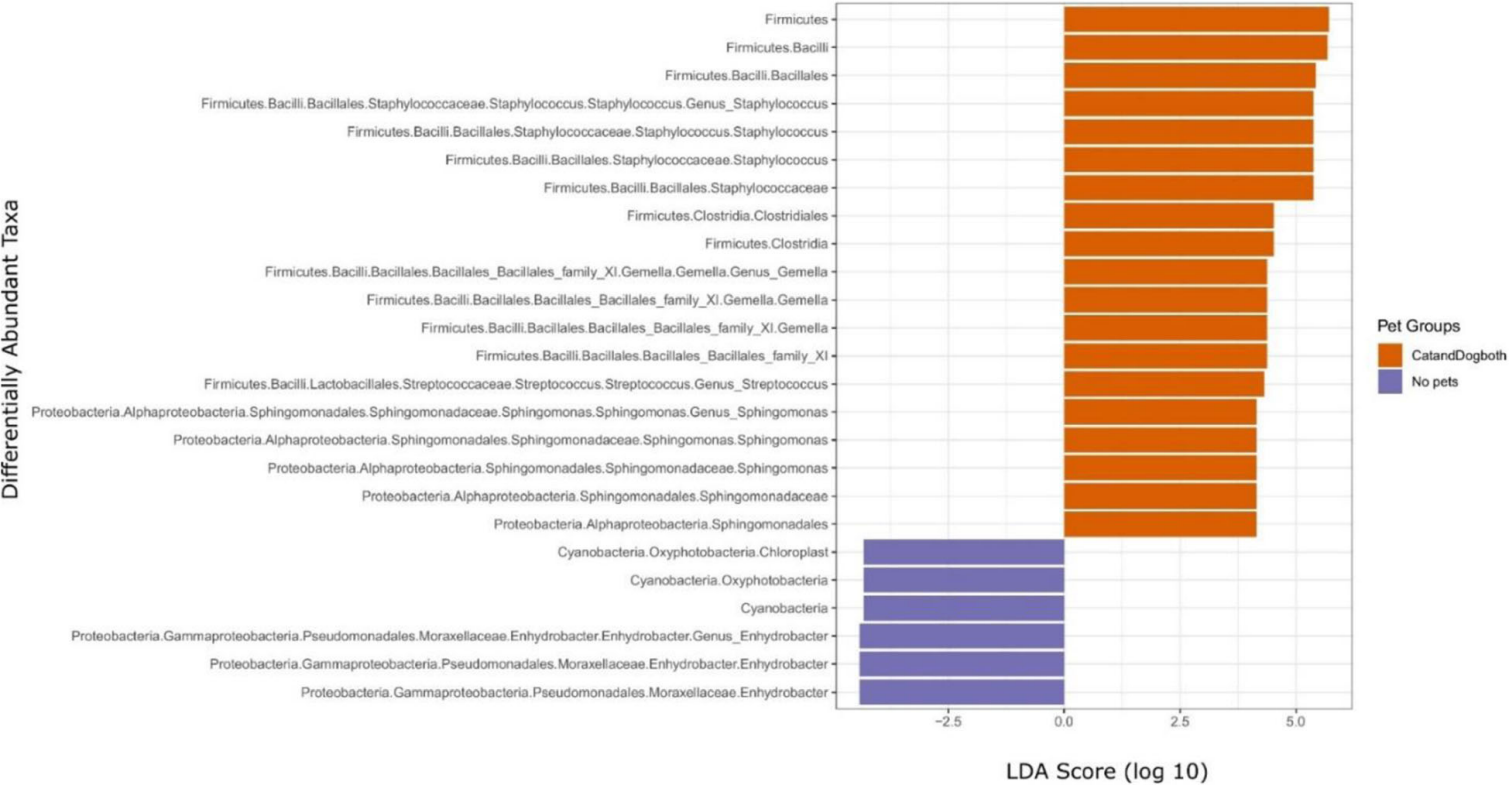
Different abundances of bacterial communities between homes with both cat and dog and no pets. With LEfSe for data analysis and visualization, key ASVs were identified as differentiating between homes with both cat and dog and no pets. The threshold for the logarithmic LDA score was 4 and *p* < 0.05 for the factorial Kruskal-Wallis test among classes

We used the phylogeny-based weighted UniFrac method to assess the relatedness between samples from homes that had dogs and/or cats using principal coordinate analysis (PCoA). We found a small, but significant, effect on the bacterial community composition (*p*_PERMANOVA_ = 0.003, *R*^2^ = 0.007), but no significant effect on the fungal community composition (*p*_PERMANOVA_ = 0.9, *R*^2^ = 0.004) (Fig. S10). Compared to homes without cats or dogs, we found that homes that had both cat and dog had over-representation of 19 taxa in infant beds, belonging to the phyla Firmicutes and Proteobacteria (Fig. 4, Table S7), and under-representation of 6 taxa, belonging to Cyanobacteria and Proteobacteria. Among these phyla, genera such as *Gemella*, *Staphylococcus*, and *Sphingomonas* were significantly over-represented (log_10_(LDA score) > 4, *p* < 0.05), and *Enhydrobacter* genera were significantly under-represented (log_10_(LDA score) > 4, *p* < 0.05).

In homes with either a cat or a dog, the bacterial microbiota appeared to be less influenced than homes with both (Fig. 3). Homes with a dog had over-representation of 21 taxa compared to homes that had no pets, belonging to the phyla Firmicutes, Fusobacteria, Proteobacteria, Cyanobacteria, and Actinobacteria, whereas homes with a cat had over-representation of 3 taxa, belonging to phylum Actinobacteria (Table S8). Genera belonging to *Paeniclostridium*, *Atopobium*, *Tychonema*, and *Acinetobacter* (log_10_(LDA score) > 3.5, *p* < 0.05) were significantly more abundant in the homes that have only dog, whereas *Turicella* (log_10_(LDA score) > 3.5, *p* < 0.05) was significantly more abundant in the homes that have only cat (Table S9).

Furthermore, homes with both cat and dog had over-representation of 38 taxa, belonging to the phyla Ascomycota, Basidiomycota, Chytridiomycota, and Mortierellomycota. Genera belonging to *Neophaeosphaeria*, *Mortierella*, *Preussia*, *Tylospora*, *Spizellomyces*, *Oleoguttula*, and *Monilochaetes* (log_10_(LDA score) > 3.5, *p* < 0.05) were the significant ones that were over-represented in the homes that had both cat and dog (Table S10). Genera belonging to *Setosphaeria*, *Peziza*, *Melanogaster*, *Lodderomyces*, *Preussia*, *Curvularia*, and *Dirkmeia* (log_10_(LDA score) > 3.5, *p* < 0.05) (Table S11) were significantly more abundant in the homes that have only dog, whereas *Oleoguttula*, *Curvularia*, and *Caloplaca* (log_10_(LDA score) > 3.5, *p* < 0.05) were significantly more abundant in the homes that have an only cat (Table S12).

### Influence of living environment (rural or urban) on bed dust microbiome

Bacterial richness and composition of the bed dust were highly influenced by the living environment (rural or urban) (Table 1). We further investigated and identified the taxa at genus level between rural and urban living environments. We found 353 genera in the bed dust from rural environment that were not present in the bed dust from urban environment (Table S13, Fig. S11a). In addition, we performed differentially abundant analysis using Wilcoxon tests to identify the taxa with significantly different abundance between the two groups (Fig. S11b). *Paracoccus*, *Micrococcus*, and *Sphingomonas* were the top three significantly more abundant taxa in the rural environment (adjusted *p* value <0.05). Moreover, genera belonging to order *Rickettsiales* were significantly more abundant in the urban environment (adjusted *p* value <0.05).

### Seasonal effect on other environmental factors

To test for interactions between the season of sampling and the effect of environmental variables in the bed dust, we stratified the significant variables from Table 1 by the season of sampling. The effect of “type of living environment” and “type of home” on microbial diversity was consistent across seasons. However, the homes with both cat and dog only had a higher bacterial diversity in their bed dust when sampled in the fall and winter (Table S14).

### Correlations between the dust and airway microbiota

**Fig. 5:**
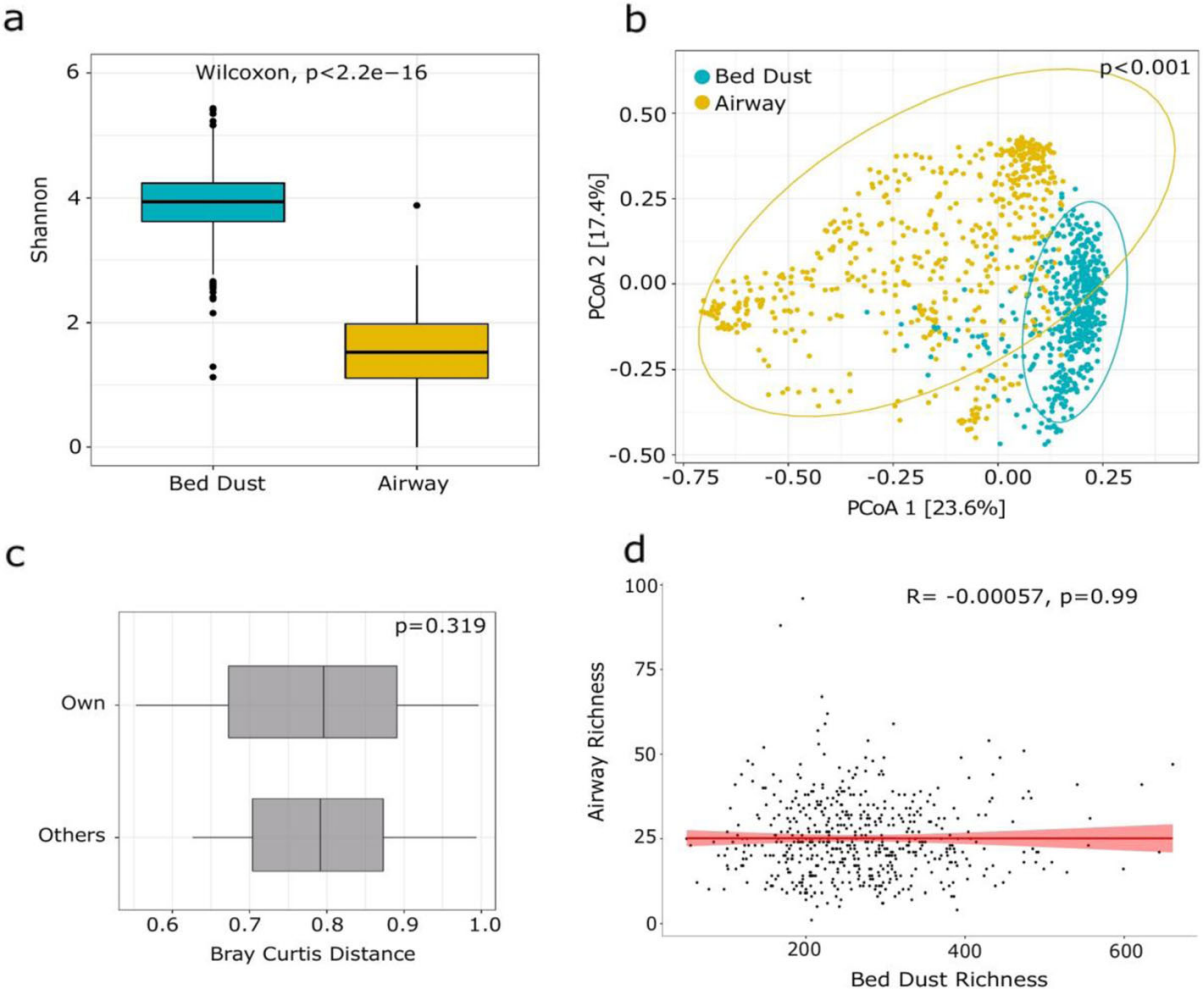
Alpha and beta diversity comparison of airway and bed dust samples. **a** Box plot showing the Shannon diversity. Highly significant differences were observed in the diversity (Wilcoxon test, *p* < 2.2e−16) between airway and bed dust samples. **b** Distances shown in the PCoA plot are based on Bray-Curtis diversity metrics. The bacterial microbiome of each sample is indicated with one dot. **c** Bray-Curtis distance between the dust-airway sample pairs for a specific child compared to the other random sample pair. “Own” represents the distance between specific children bed dust with their own airway samples. “Others” represents the distance between random pairs of children bed dust with random airway samples. **d** Associations between fungal and bacterial alpha diversity (observed richness) for a given sample. The shaded gray region represents 95% confidence intervals. Linear regression analysis: *p* = 0.99, *R* = − 0.00057 for observed richness

Next, we evaluated whether associations existed between the two microbial compartments (beds dust when infants were 6 months old and airways at 3 months), possibly alluding to the importance of the dust microbiota on the infant airway composition. We observed significantly higher bacterial alpha diversity in bed dust compared to infant airways (Wilcoxon test, adjusted *p* < 1e−15) (Fig. 5a). Furthermore, bed dust and airway microbiota separated well by Bray-Curtis distance measures (*P*_PERMANOVA_ < 0.001) (Fig. 5b).

We applied several methods to identify relationships between bed dust and airway microbiota. Based on the Spearman correlations, we did not observe any significant correlations in alpha diversity (*p* = 0.9) (Fig. 5d). Furthermore, we tested for transfer between the dust and airway bacteria using presence-absence of shared genera and odds ratio analysis. Interestingly, we did not identify any significant sharing of genera using this method (Fig. S12). In addition, bed dust and airway samples from the same child were not more similar to each other than randomly paired dust and airway samples using Bray-Curtis distance (Wilcoxon test, adjusted *p* = 0.3) (Fig. 5c).

**Fig. 6:**
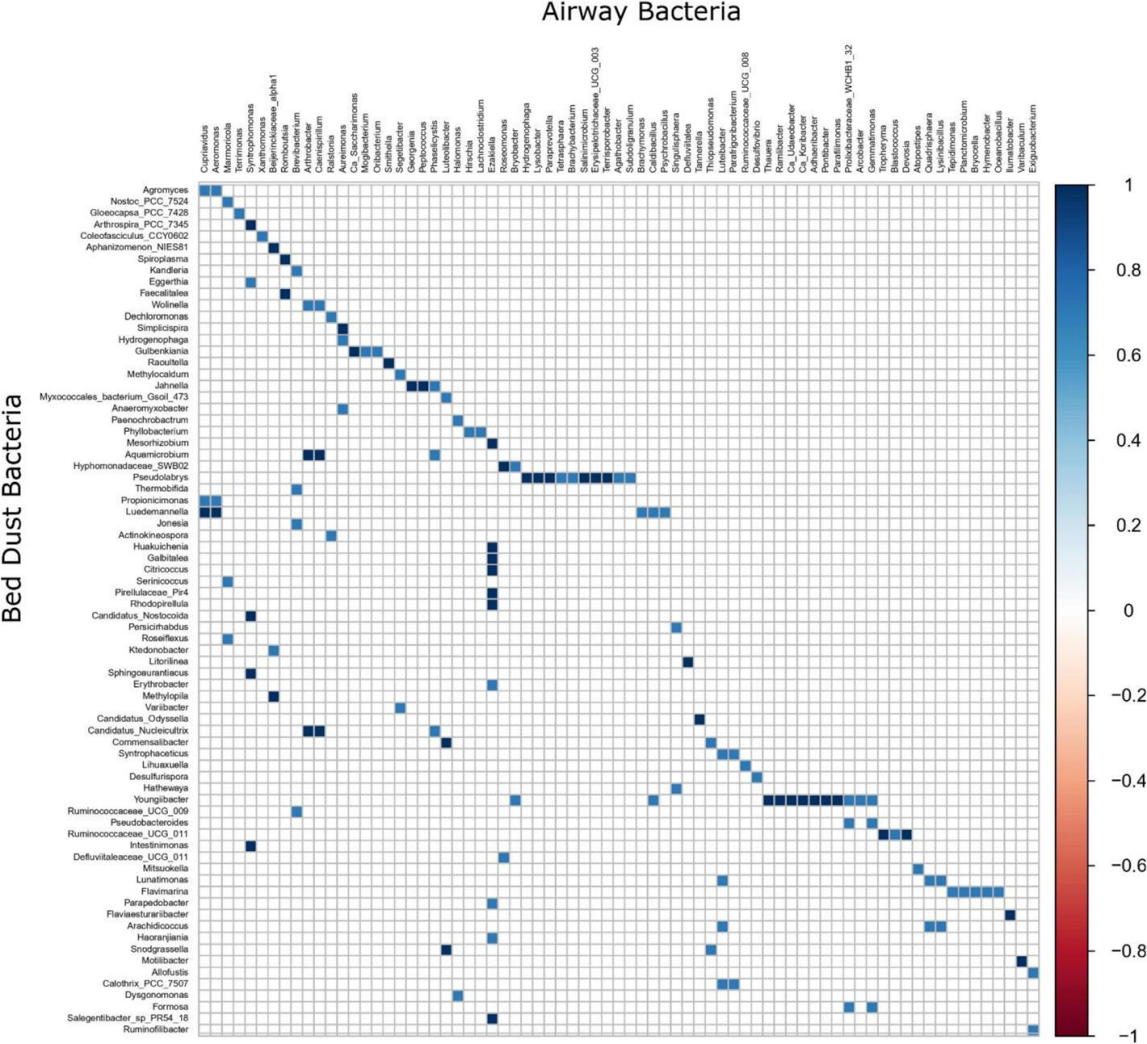
Spearman correlation by genus abundance. Only significant values (*p* < 0.05 after FDR adjustment) are shown. Correlation inferred for bacterial bed dust microbiome with bacterial airway microbiome. Orange and blue colors represent significant negative correlations and positive correlations. Darker color represents stronger correlations

When taking into account the relative abundance and looking for correlations, we found that the fungal community composition in bed dust did not show significant correlations with the airway bacterial community. However, we observed several bacterial genera in the bed dust that correlated significantly with bacterial abundances in the infant airways (*p* < 0.01) (Fig. 6). For example, *Youngiibacter* and *Pseudolabrys* in dust samples had many positive correlations with genera from the airway samples. Moreover, multiple genera in the bed dust samples such as *Arachidicoccus*, *Pseudosphingobacterium*, *Calothrix*, and *Syntrophaceticus* showed positive correlations with *Luteibacter* among airway samples.

## Discussion

In this study, we determined that bacterial and fungal communities in bed dust are related to each other with positive correlations in alpha diversity and that they are both influenced by environmental factors. The presence of pets and type of living environment (rural or urban) are the dominant factors among those studied that most affect microbial communities.

We observed that the fungal microbiota composition of bed dust samples was dominated by fungi from the phyla Ascomycota and Basidiomycota (Fig. S4a). In accordance with previous work, *Aureobasidium* and *Penicillium* genera have commonly been identified in homes [27] at several sites such as floors [28] and kitchen sinks [29]. These shared features in community composition indicate that common taxa present elsewhere in homes are likely to be discovered in beds also.

Moreover, the bed dust samples were dominated by Gram-positive bacteria, including genera known to be associated with human sources such as *Staphylococcus*, *Streptococcus*, and *Corynebacterium* (Fig. S3d). These bacterial genera, which are commonly found on human skin [30], have been documented in other studies of the home microbiomes as well [31, 32]. The human contribution to bacteria within the home is further confirmed in a study [5] showing the abundance patterns of bacterial taxa in samples from homes that closely resemble the microbial profiles of its human residents.

Environmental factors such as pets had a significant influence in shaping the bed dust microbiomes. Studies have shown that bacterial diversity increases significantly by the presence of a dog (but not by a cat) in a household [4, 14]. We observed that bacterial diversity was increased significantly by the presence of both cat and dog in the home (Fig. 3) but not if either one was present alone. Many taxa associated with pet ownership have previously been associated with human health outcomes. For example, *Corynebacterium* and *Staphylococcus* genera were associated with the homes that have both dog and cat. These genera are found mostly on skin and nose and may play an active role in host defense [33]. *Acinetobacter* was associated with homes that have only a dog. These genera, part of the human skin microbiota, may protect against allergic sensitization and inflammation [34].

Some studies have reported that the living environment (rural or urban) has little to no significant effect on bacterial or fungal diversity [35], whereas others have shown significant changes in the microbial diversity [36]. In our study, the effect of urbanization showed a significant effect on both bacterial and fungal diversity on the bed dust microbiome. Approximately 30% of bacterial genera present in the bed dust from rural areas were not found in bed dust samples collected from an urban living environment (Fig. S11). While a wide range of factors can influence the risk of developing asthma, rates of allergic asthma are higher for children living in more urbanized areas than in rural areas [37]. It has been hypothesized that these geographic differences in allergy rates can be attributed to people living in more urbanized areas being exposed to lower levels of microbial diversity [38], and our study supports the hypothesis.

Furthermore, we observed that having siblings in the household correlated with increased bacterial and fungal richness, similar to the data from Weikl et al. (2016) who found that households with more than three occupants had higher bacterial richness [39]. Earlier studies showed that fewer siblings in early life are associated with increased risks of developing asthma and other atopic diseases later in life [40]. Moreover, higher microbial diversity in the environment has been found to be inversely associated with asthma [25]. Together with our results, these suggest that siblings protect against asthma and atopic disease by increasing the bacterial and fungal richness, but further studies are needed to confirm this.

As no study we know of have investigated how the sex or siblings affect the bacterial or fungal richness, we stratified for the sex of siblings. Interestingly, we found that only male siblings significantly increased bacterial and fungal richness and correlated with the number of male siblings. In homes with only female siblings, fungal richness were significant increased, but we did not have the statistical power to determine if the trend towards higher bacterial richness were significant. Additionally, we did not find any significant differences in the richness when comparing homes with either female or male siblings. While not directly comparable to our study population, it has been suggested that men shed more bacteria to their environment [7] and a study by Raju et al. found higher bacterial richness in saliva of boys compared to girls [41].

Interestingly, alpha diversity of the bacterial and fungal microbiome of the bed dust was significantly and positively correlated (Fig. 1c). To further understand the association between the bacterial and fungal microbiome in the bed dust samples, we observed numerous significant correlations at the genus level. In the fungal-fungal correlations, we observed mostly positive correlations with only *Saccharomyces* having negative correlations (to *Spegazzinia*, *Curvularia*, and *Sphaerellopsis*). *Saccharomyces* is a common genus in home dust and usually associated with humans [42]. For bacteria-bacteria correlations, the most abundant genera *Staphylococcus* and *Streptococcus* showed both positive and negative correlations with other genera. *Staphylococcus* and *Streptococcus* are a typical part of the human microbiome and constantly interact with each other [30]. Moreover, we also observed an inter-domain correlation between bacteria and fungi and most of these correlations were positive, pointing at synergistic relationships or that they were transported to the bed dust together from the same source.

Not surprisingly, the bed dust bacterial microbiota was different from the airway bacterial microbiota. While both airway and bed dust samples harbored diverse microbial communities, the diversity of bacteria was higher in bed dust samples (Fig. 5a). The airway samples were dominated by genera from the families Streptococcaceae and Staphylococcaceae, as well as the genus *Moraxella*, which are mainly observed in the upper respiratory tracts of healthy children [43]. In our comparisons of bed dust and airway microbiota, based on OR, we did not observe any significant taxa that were shared between bed dust and infants’ airway. However, based on the microbial relative abundance, we observed several positive correlations between airway bacterial and bed dust bacterial and fungal microbiomes. The biological significance of these positive correlations remains unknown, but as we found limited evidence of transfer, shared exposure reservoirs may be the cause. However, we cannot exclude the possibility that the difference in sampling time between the bed dust and airway samples might have caused this lack of evidence for transfer.

Our study has some limitations. We have used different DNA extraction kits for the airway and dust samples. Different types of extraction kits may have different biases in extraction efficiency, which in turn may affect the bacterial composition results. Moreover, we have used different sequencing primers for dust and airway microbiome (V3–V4 region for the bed dust samples and V4 region for the airway samples), and different PCR primers preferentially amplify different sets of taxa [44, 45]. This may hamper the identification of transfer events on the ASV level. However, this should not affect the correlation analysis between the two compartments. Furthermore, we have collected the children’s airway samples at 3 months of age whereas and the bed dust samples 3 months later. Collecting the samples at the same time point would have provided a stronger premise for association analyses, especially as seasonal differences in the microbiomes were found. However, we also observed that the sampling season did not seem to interact with the effect of other environmental variables, with the exception of the effect of pet ownership that was only found in the fall and winter. During fall and winter months, Danish people are likely to spend less time outdoors, but as pets need to visit there regularly during every season, they can act as vectors of bacteria from the outside. However, the stratification results in groups with quite low numbers, which can result in spurious findings, and should thus be interpreted with caution. Lastly, many of the fungal sequencing reads could not be assigned to specific taxa (10.53% unclassified at the phylum level, 47.25% of reads being unclassified class level); this did limit our analysis and indicates that it might be relevant to revisit our analysis when better fungal reference databases are available.

## Conclusion

In summary, our study finds evidence of interplay between bacterial and fungal diversity in the bed dust of young infants and that both bacterial and fungal composition are affected by environmental variables. We find limited evidence of transfer between the dust and developing airway microbiota. From early life, children are exposed to a multitude of environmental exposures, which may impact a healthy development perhaps through the microbiome of bed dust, which may act as the interface between environment and child.

## Methods

### Study design and sample collection

The study was embedded in the population-based COPSAC_2010_ prospective mother-child cohort of 736 women and their children followed from week 24 of pregnancy [26].

Beds dust was sampled by the parents when the infants were 6 months old. This was done using an external filter kit (DUSTREAM® Collector, Indoor Biotechnologies, or Dust Collecting Device from ALK-Abello) attached to the family’s vacuum cleaner with instructions to vacuum the sheets and pillow for 5 min. Filters were then kept in the freezer for 3 days to kill dust mites and shipped to COPSAC where they were kept at − 20 °C until DNA extraction. The infant airway was sampled using hypopharyngeal aspirates obtained at 3 months of age, using a soft suction catheter passed through the nose and stored at − 80 °C until DNA extraction [23].

### Covariates

Information on educational level, household income, pet ownership, race, type of home, and home address was obtained during the scheduled visits to the research clinic. Living environment (rural/urban) was defined based on the land cover in a 3-km radius based on children’s birth address as previously described (Lehtimäki et. al., under review).

### DNA extraction and amplification

Dust was released from the filter boxes, and 250 mg was used for DNA extraction using the NucleoSpin® 96 Soil DNA Isolation Kit optimized for epMotion® (MO-BIO Laboratories, Inc., Carlsberg, CA, USA) using the epMotion® robotic platform model (Eppendorf) under manufacturer’s protocol. The bed dust samples were profiled with bacterial as well as fungal community using amplicon sequencing, using a two-step protocol. In the first step, we amplified the community specific rRNA target using general primers, and in the second step, we used primers with sequencing adaptors, barcodes, and the target sequence, so each sample could be uniquely identified post-sequencing. For fungi, we targeted the internal transcribed spacer, region 2 (ITS2), with the primers gITS7F (5′- GTGARTCATCGARTCTTTG-3′) and ITS4ngs (5′- TTCCTSCGCTTATTGATATGC-3′). For bacteria, we targeted variable region V3–V4 of the 16S rRNA gene, using forward primer 341f (5′- CCTAYGGGRBGCASCAG-3′) and reverse primer 806r (5-GGACTACHVGGGTWTCTAAT-3). Negative controls were included for the extraction and PCR amplification procedures. All final PCR products were purified using HighPrep^TM^ PCR (MAGBIO, USA), based on paramagnetic beads technology. Then, it was normalized using SequalPrep^TM^ Normalization plate kit (Invitrogen, USA). Further cleaning and concentration were done by using the DNA Clean & Concentrator^TM^-5 Kit (Zymo Research, Irvine, CA, USA). Concentrations were then determined using the Quant-iT™ High-Sensitivity DNA Assay Kit (Life Technologies).

The airway samples from the children at 3 months of age used in this study was a part of COPSAC_2010_ cohort that was already published in Mortensen et al. [20] and Gupta et al. [43]. Genomic DNA was extracted for airway samples using the PowerMag® Soil DNA Isolation Kit optimized for epMotion® (MO-BIO Laboratories, Inc., Carlsberg, CA, USA) using the epMotion® robotic platform model (Eppendorf) under manufacturer’s protocol. The airway microbiota were profiled with the same method, but only for bacteria, targeting variable region V4 of the 16S rRNA gene, using forward primer 515f (5′-GTGCCAGCMGCCGCGGTAA-3′) and 806r (5′-GGACTACHVGGGTWTCTAAT-3′). The rest of the steps were the same as mentioned above for dust samples.

### Sequencing

Paired-end sequencing was performed on the Illumina MiSeq System (Illumina Inc., CA, USA), including 5% PhiX as an internal control. All reagents used were from the MiSeq Reagent Kits v3 (Illumina Inc., CA, USA) for bed dust samples and MiSeq Reagent Kits v2 for airway samples. Automated cluster generation and paired-end sequencing with dual-index reads were performed with 2 × 300 bp for bed dust samples and 2 × 250 bp for airway samples. The sequencing output was generated as demultiplexed fastq-files for downstream analysis.

### Sequence analysis

Primers were removed from the raw paired-end FASTQ files generated via MiSeq using “cutadapt” [46]. Further, reads were analyzed by QIIME2 [47] (qiime2-2018.11) pipeline through dada2 [58] to infer the ASVs present and their relative abundances across the samples. For bed dust samples, using read quality scores for the dataset, forward and reverse reads were truncated at 270 bp and 220 bp, followed by trimming the 5′ end till 8 bp for both forward and reverse reads, respectively; other quality parameters used dada2 default values for both 16S rRNA gene and ITS sequencing. For airway samples, forward and reverse reads were truncated at 180 bp and 160 bp; other parameters remain the same as mentioned above. For 16S rRNA gene sequencing, taxonomy was assigned using a pre-trained Naïve Bayes classifier (Silva database, release 132, 99%ASV) [48], and for ITS sequencing, UNITE database (dynamic-2017-12-01) [49] were used.

### Quality control

To ensure that our analyses were not confounded by spurious results, we first analyzed the alpha diversity of negative control samples (including PCR negative, extraction control) that produced sequencing reads and dust samples (Fig. S1). The DNA extraction and other negative controls had significantly lower observed richness than all dust samples (analysis of variance (ANOVA), *p* < 0.05) for both fungal and bacterial data. Furthermore, profiles were significantly different for both bacterial and fungal microbiome by sample type (Fig. S2a, b). Sequencing contaminants (75 of 79,347 bacterial and 141 of 24,474 fungal ASVs) were identified based on the prevalence of ASVs in the negative control and removed using the decontam package (default parameters); this did not measurably affect the microbiota structure (Fig. S2c, d). We then removed the PCR and sequencing controls before downstream analysis. Moreover, samples that did not have a satisfactory sequencing of both 16S rRNA gene (minimum 3,000 reads per sample) and ITS (minimum 3,000 reads per sample) were removed. For airway samples, sequencing contaminants (14 of 3,692 bacterial ASVs) were identified based on the prevalence of ASVs in the negative control and removed using the decontam package (default parameters). The DNA extraction and other negative controls had significantly lower observed richness than all dust samples (analysis of variance (ANOVA), *p* < 0.05) (Fig. S2e).

### Statistical analysis

Data analysis was conducted in R (R Core Team, 2017). Initial preprocessing of the ASV table was conducted using the phyloseq package (v1.20.0) [50]. Further filtering was done by removing ASVs classified as archaea, chloroplast, or without phylum-level classification, from 16S rRNA gene sequencing data as well as Rhizaria from ITS sequencing data. Sequencing contaminants were identified and removed using the decontam package [51]. To avoid the bias due to sampling depth, the ASVs table was multiple rarefied [43] to 6774 high-quality sequences per bed dust sample for 16S rRNA gene, 9,942 per bed dust sample for ITS, and 1,957 per sample for airway 16S rRNA gene.

All downstream analyses were performed on this rarefied ASVs table unless mentioned. We used three alpha diversity indices, i.e., observed richness, Shannon diversity index, and Chao1 index. Furthermore, beta diversity was calculated using weighted and unweighted UniFrac metric and visualized by principal coordinates analysis (PCoA). Alpha and beta diversity was calculated using phyloseq v1.20.0 and visualized with ggplot2 v2.2.1 [52] in R v3.4.1. Comparison of community richness and diversity was assessed by the Kruskal-Wallis test between all the groups, and comparison between the two groups was done by Wilcoxon test with Benjamini-Hochberg FDR multiple test correction. Significance testing between the groups for beta diversity was assessed using permutational multivariate analysis of variance (PERMANOVA) using the “vegan” package [53]. Marginal effect was calculated using the PERMANOVA analysis (for each significant environmental factor) using the following formula: adonis2(dist ~ Type_of_enviornment + Pet + Type_of_home + Siblings + Season, by =”margin”) for beta diversity.

### Microbial correlation and differentially abundant analysis

Considering the variable nature of 16S compositional data, we estimated the core microbial group of ASVs within the samples with a presence in at least 30% of the study samples. The correlation analyses were performed at the genus level of the bed dust and airway samples. To better understand the dust community structure, characterize intra-community interactions, and identify potentially shared niches, the co-occurrence network analysis was performed and visualized by R. Spearman correlation analysis built into the function “rcorr” from the package “Hmisc” [54] was used to calculate the association at the genus level. *p* values were adjusted for comparisons with the false discovery rate (FDR) algorithm after compositional transformation. The significance of the correlation adjusted *p* value< 0.01 was the threshold to define significant correlations. The correlation matrix of genera was visualized by the function “corrplot” in the package “corrplot” [55]. The linear regression analysis was visualized by the function “ggscatter” in the package “ggpubr” [56]. We analyzed the transfer of bacteria from bed dust to airway microbiome using Fisher’s exact test by comparing the presence/absence of bacteria (at genus level) and calculated the odds ratio for transfer with a one-sided *p* value. Only bacteria (at genus level) showing presence/absence in both the bed dust as well as the airway were included in the analysis. Inference for transfer of single bacteria (at genus level) was evaluated using Benjamini-Hochberg FDR correction. Furthermore, LEfSe [57] was used to identify the microbiological markers associated with a pet by linear discriminant analysis (LDA) effect size of cutoff 3.5. Other parameters were kept default. For rural and urban living environment, we have used the Wilcoxon tests.

## Supplementary information

**Additional file1: Fig. S1.** Rarefaction curves of the (a) Bacteria (16S rRNA gene), and (b) Fungi (ITS) samples. **Fig. S2.** Influence of potential contaminant ASVs. **Fig. S3.** Bacterial microbiome by 16S rRNA gene (panels a–d) at four taxonomic levels (a) phylum (b) class (c) family, and (d) genus. **Fig. S4.** Fungal microbiome by ITS (panels a–d) at four taxonomic levels (a) phylum (b) class (c) family, and (d) genus. **Fig. S5.** Spearman correlation between fungi in bed dust. **Fig. S6.** Spearman correlation between bacteria in bed dust. **Fig. S7.** Box plots of the bacterial richness according to home type and living environment. **Fig. S8.** Box plots of the fungal richness according to home type and living environment. **Fig. S9.** Box plots of fungal alpha-diversity according to pet ownership. **Fig. S10.** PCoA plot of (a) bacterial and (b) fungal community composition based on weighted Unifrac distance. **Fig. S11.** Differentially abundant analysis between rural and urban living environment. **Fig. S12.** The odds for transfer of taxa (at genus level) from dust to airway microbiota of children. **Table S1.** Characteristics of the study population. **Table S2.**Bacterial abundance in bed dust samples at phylum level. **Table S3.** Bacterial abundance in bed dust samples at genus level. **Table S4.** Fungal abundance in bed dust samples at phylum level. **Table S5.** Fungal abundance in bed dust samples at class level. **Table S6.** Fungal abundance in bed dust samples at genus level. **Table S7.** Differentially abundant bacterial taxa in bed dust samples for homes with dog and cat both. **Table S8.** Differentially abundant bacterial taxa in bed dust samples for homes with dog. **Table S9.** Differentially abundant bacterial taxa in bed dust samples for homes with cat. **Table S10.** Differentially abundant fungal taxa in bed dust samples for homes with dog and cat both. **Table S11.** Differentially abundant fungal taxa in bed dust samples for homes with dog. **Table S12.** Differentially abundant fungal taxa in bed dust samples for homes with cat. **Table S13.** Bacteria present in rural and urban environment at genus level. **Table S14.** The effects of season and environmental factors on bed dust alpha and beta-diversity. **Table S15.** The adjusted (marginal) effects of environmental factors on bed dust alpha and beta-diversity. 

## Data Availability

The dataset analyzed during the current study will be available, upon publication, in the Sequence Read Archive (SRA) repository under project id PRJNA605085 for bed dust samples (both bacterial and fungal raw sequencing reads). Airway samples were already published in our previous article and it is available in the Sequence Read Archive (SRA) repository, http://www.ncbi.nlm.nih.gov/bioproject/340273. All other data is available from the corresponding author.
