# Supplementary material for: Environmental shaping of the bacterial and fungal community in infant bed dust and correlations with the airway microbiota
Source: Microbiome. 2020 Aug 7;8:115. doi: 10.1186/s40168-020-00895-w (PMC7414761; doi:10.1186/s40168-020-00895-w)
Supplement: Supplementary file 2 — Additional file1: Fig. S1. Rarefaction curves of the (a) Bacteria (16S rRNA gene), and (b) Fungi (ITS) samples. Fig. S2. Influence of potential contaminant ASVs. Fig. S3. Bacterial microbiome by 16S rRNA gene (panels a–d) at four taxonomic levels (a) phylum (b) class (c) family, and (d) genus. Fig. S4. Fungal microbiome by ITS (panels a–d) at four taxonomic levels (a) phylum (b) class (c) family, and (d) genus. Fig. S5. Spearman correlation between fungi in bed dust. Fig. S6. Spearman correlation between bacteria in bed dust. Fig. S7. Box plots of the bacterial richness according to home type and living environment. Fig. S8. Box plots of the fungal richness according to home type and living environment. Fig. S9. Box plots of fungal alpha-diversity according to pet ownership. Fig. S10. PCoA plot of (a) bacterial and (b) fungal community composition based on weighted Unifrac distance. Fig. S11. Differentially abundant analysis between rural and urban living environment. Fig. S12. The odds for transfer of taxa (at genus level) from dust to airway microbiota of children. Table S1. Characteristics of the study population. Table S2.Bacterial abundance in bed dust samples at phylum level. Table S3. Bacterial abundance in bed dust samples at genus level. Table S4. Fungal abundance in bed dust samples at phylum level. Table S5. Fungal abundance in bed dust samples at class level. Table S6. Fungal abundance in bed dust samples at genus level. Table S7. Differentially abundant bacterial taxa in bed dust samples for homes with dog and cat both. Table S8. Differentially abundant bacterial taxa in bed dust samples for homes with dog. Table S9. Differentially abundant bacterial taxa in bed dust samples for homes with cat. Table S10. Differentially abundant fungal taxa in bed dust samples for homes with dog and cat both. Table S11. Differentially abundant fungal taxa in bed dust samples for homes with dog. Table S12. Differentially abundant fungal taxa in bed dust samples for homes [file 40168_2020_895_MOESM1_ESM.doc]

# **Supplementary figure legends**

**Fig. S1.** **Rarefaction curves of the (a) Bacteria (16S rRNA gene), and (b) Fungi (ITS) samples.** The rarefaction curves were calculated using the richness and Shannon diversity index at increasing sequencing depth. Error bars represent standard deviation.

**Fig. S2. Influence of potential contaminant ASVs.** (a) β diversity by sample type prior to removal of potential contaminant ASVs using the decontam package for 16S rRNA samples. (b) β diversity by sample type prior to removal of potential contaminant ASVs using the decontam package for ITS samples. β diversity was assessed on Bray-Curtis dissimilarity and tested by PERMANOVA. There was a significant difference based on the sample type. (c) Composition of the ASVs before and after contaminant removal at class level (>1% mean relative abundance) for 16S rRNA samples. (d) Composition of the ASVs before and after contaminant removal at class level (>1% mean relative abundance) for ITS samples. (e) β diversity by sample type prior to removal of potential contaminant ASVs using the decontam package for airway samples. β diversity was assessed on Bray-Curtis dissimilarity and tested by PERMANOVA. There was a significant difference based on the sample type.

**Fig. S3.** **Bacterial microbiome by 16S rRNA gene (panels a–d) at four taxonomic levels (a) phylum (b) class (c) family, and (d) genus.** Each of the above-stacked bar plots illustrates the average relative abundance (y-axis) of the microbiota at different taxonomic levels. Taxa with a mean abundance of at least 1% across all samples are represented in colors; those with < 1% abundance are not shown. Each column represents one individual. The taxonomy for bacterial ASVs was assigned from the SILVA database.

**Fig. S4.** **Fungal microbiome by ITS (panels a–d) at four taxonomic levels (a) phylum (b) class (c) family, and (d) genus.** Each of the above-stacked bar plots illustrates the average relative abundance (y-axis) of the microbiota at different taxonomic levels. Taxa with a mean abundance of at least 1% across all samples are represented in colors; those with <1% abundance are not shown. Each column represents one individual. The taxonomy for bacterial ASVs was assigned from the UNITE database.

**Fig. S5.** **Spearman correlation between fungi in bed dust.** Only significant values (p<0.05 after FDR adjustment) are shown. Orange and blue represents significant negative correlations and positive correlations. Darker color represents stronger correlations.

**Fig S6.** **Spearman correlation between bacteria in bed dust.** Only significant values (p<0.05 after FDR adjustment) are shown. Orange and blue represents significant negative correlations and positive correlations. Darker color represents stronger correlations.

**Fig. S7. Box plots of the bacterial richness according to home type and living environment.** (a) differences between apartment and house in the rural and urban areas, (b) differences between rural and urban in the house and apartment.

**Fig. S8. Box plots of the fungal richness according to home type and living environment.** (a) differences between apartment and house in the rural and urban areas, (b) differences between rural and urban in the house and apartment.

**Fig. S9. Box plots of fungal alpha-diversity according to pet ownership.** Box plots of the three diversity metrics (a) Observed, (b) Shannon diversity, and (d) Chao1 diversity.

**Fig. S10.** **PCoA plot of (a) bacterial and (b) fungal community composition based on weighted Unifrac distance.** PCoA plot showing the relationship among samples belongs to a home that owns a pet.

**Fig. S11. Differential abundance analysis between rural and urban living environment.** (a) Venn diagram depicting the bacterial genera constantly present in the rural and urban living environment. (b) Relative abundances in the bed dust samples associate with living environment (rural or urban). Comparison among the 30 most significantly abundant bacterial genera. P-values correspond to Wilcoxon rank-sum tests of the relative abundances, with significant values (p < 0.05) bolded with FDR correction. A pseudocount (+1e−06) was added to all abundances for the log-scale presentation. The black dots indicate median values and the abundances are colored according to the rural (red) (N = 251) or urban (blue) (N = 295) living environment.

**Fig. S12.** **The odds for transfer of taxa (at genus level) from dust to airway microbiota of children.** Top panel shows the odds ratio (x-axis) and the strength (p-value). Of particular interest is the distribution of positive- (odds ratio>1) compared to negative odds (odds ratio<1). Lower panel shows odds ratio (y-axis) versus the population-wide dust abundance (x-axis). Odds larger (or smaller) than 100 fold are truncated to 100 (or 0.01). Colors indicate the top 15 overall most abundant taxonomic families.

# **Supplementary tables legends**

**Table S1.** Characteristics of the study population. *Income level is categorize into Low (<€50,000/year), medium (€50,000–€110,000/year), high (>€110,000/year). **Education level is categorize into Low (primary school, secondary school, or college graduate), medium (tradesman or bachelor degree), high (master’s degree).

**Table S2.** Bacterial abundance in bed dust samples at phylum level.

**Table S3.** Bacterial abundance in bed dust samples at genus level.

**Table S4.** Fungal abundance in bed dust samples at phylum level.

**Table S5.** Fungal abundance in bed dust samples at class level.

**Table S6.** Fungal abundance in bed dust samples at genus level.

**Table S7.** Differentially abundant bacterial taxa in bed dust samples for homes with dog and cat both. †Column labeled “Pet ownership” represents the home that have dog and cat both in which the corresponding taxa (as presented in column labeled “Differentially abundant Taxa”), was found to be significantly differentially abundant by LEfSe. ‡ Differentially abundant taxa is described using the following hierarchy: Phylum|Class|Order|Family|Genus|species.

**Table S8.** Differentially abundant bacterial taxa in bed dust samples for homes with dog. †Column labeled “Pet ownership” represents the home that have dog in which the corresponding taxa (as presented in column labeled “Differentially abundant Taxa”), was found to be significantly differentially abundant by LEfSe. ‡ Differentially abundant taxa is described using the following hierarchy: Phylum|Class|Order|Family|Genus|species.

**Table S9.** Differentially abundant bacterial taxa in bed dust samples for homes with cat. †Column labeled “Pet ownership” represents the home that have cat in which the corresponding taxa (as presented in column labeled “Differentially abundant Taxa”), was found to be significantly differentially abundant by LEfSe. ‡ Differentially abundant taxa is described using the following hierarchy: Phylum|Class|Order|Family|Genus|species.

**Table S10.** Differentially abundant fungal taxa in bed dust samples for homes with dog and cat both. †Column labeled “Pet ownership” represents the home that have dog and cat both in which the corresponding taxa (as presented in column labeled “Differentially abundant Taxa”), was found to be significantly differentially abundant by LEfSe. ‡ Differentially abundant taxa is described using the following hierarchy: Phylum|Class|Order|Family|Genus|species.

**Table S11.** Differentially abundant fungal taxa in bed dust samples for homes with dog. †Column labeled “Pet ownership” represents the home that have dog in which the corresponding taxa (as presented in column labeled “Differentially abundant Taxa”), was found to be significantly differentially abundant by LEfSe. ‡ Differentially abundant taxa is described using the following hierarchy: Phylum|Class|Order|Family|Genus|species.

**Table S12.** Differentially abundant fungal taxa in bed dust samples for homes with cat. †Column labeled “Pet ownership” represents the home that have cat in which the corresponding taxa (as presented in column labeled “Differentially abundant Taxa”), was found to be significantly differentially abundant by LEfSe. ‡ Differentially abundant taxa is described using the following hierarchy: Phylum|Class|Order|Family|Genus|species.

**Table S13.** Bacteria present in rural and urban environment at genus level.

**Table S14.** The effects of season and environmental factors on bed dust alpha and beta-diversity. ^Alpha diversity were calculated based on observed richness and significance were calculated using the Wilcoxon test (for two groups) and Kruskal-Wallis test (for three or more groups), FDR corrected. #Effects were quantified with R2, and p-values, as determined by PERMANOVA on weighted UniFrac distances. Significant adjusted p-values (p<0.05) are shown in bold. Abbreviations - R: Rural, U: Urban, H: House, A: Apartment, C: Cat, D: Dog, B: both cat and dog.

**Table S15.** The adjusted (marginal) effects of environmental factors on bed dust alpha and beta-diversity. ^Alpha diversity were calculated based on observed richness and significance were calculated using the ANOVA. #Effects were quantified with R2, and p-values, as determined by PERMANOVA on weighted UniFrac distances. Significant adjusted p-values (p<0.05) are shown in bold.

**
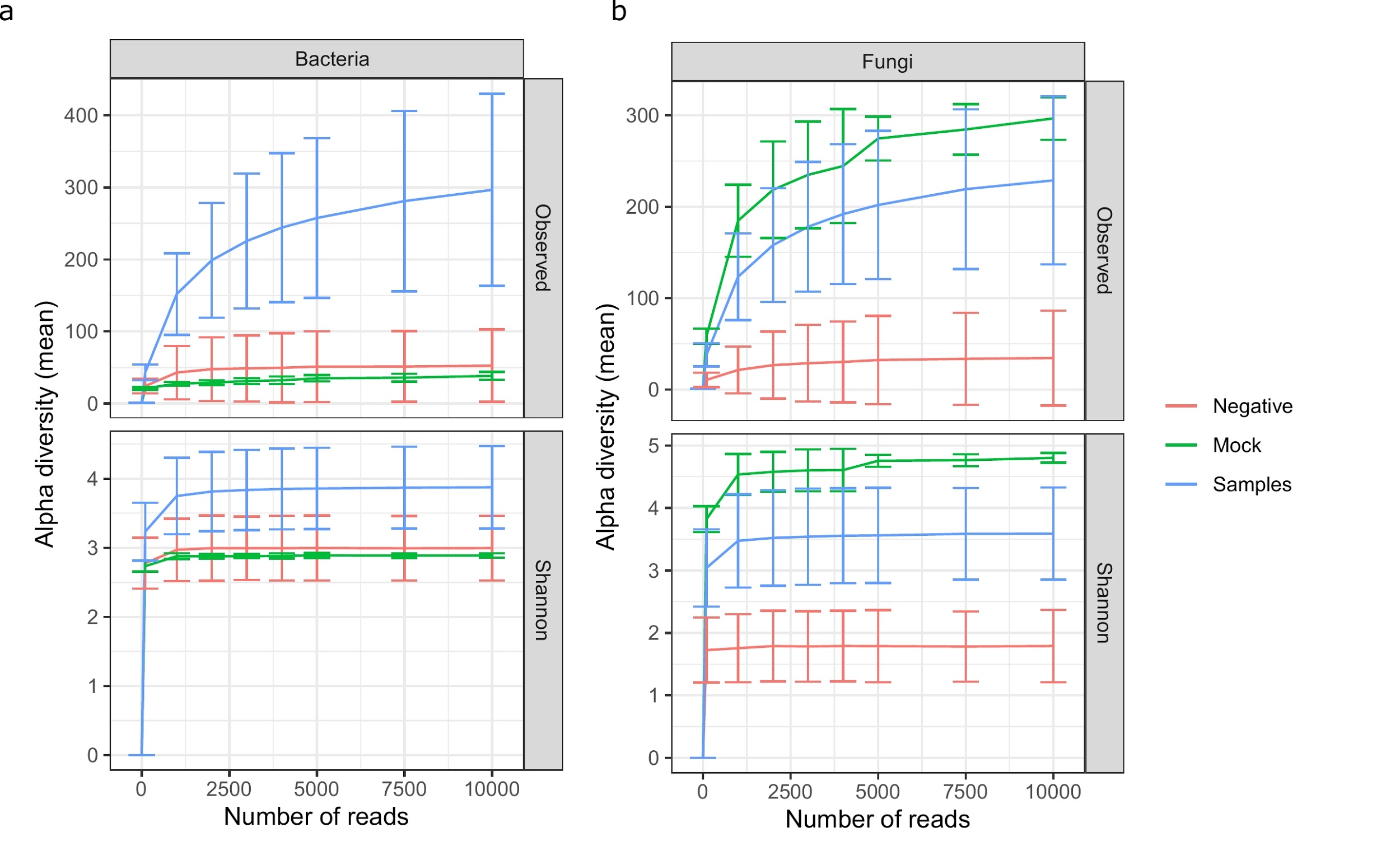
**

**Fig. S1.** **Rarefaction curves of the (a) Bacteria (16S rRNA gene), and (b) Fungi (ITS) samples.** The rarefaction curves were calculated using the richness and Shannon diversity index at increasing sequencing depth. Error bars represent standard deviation.


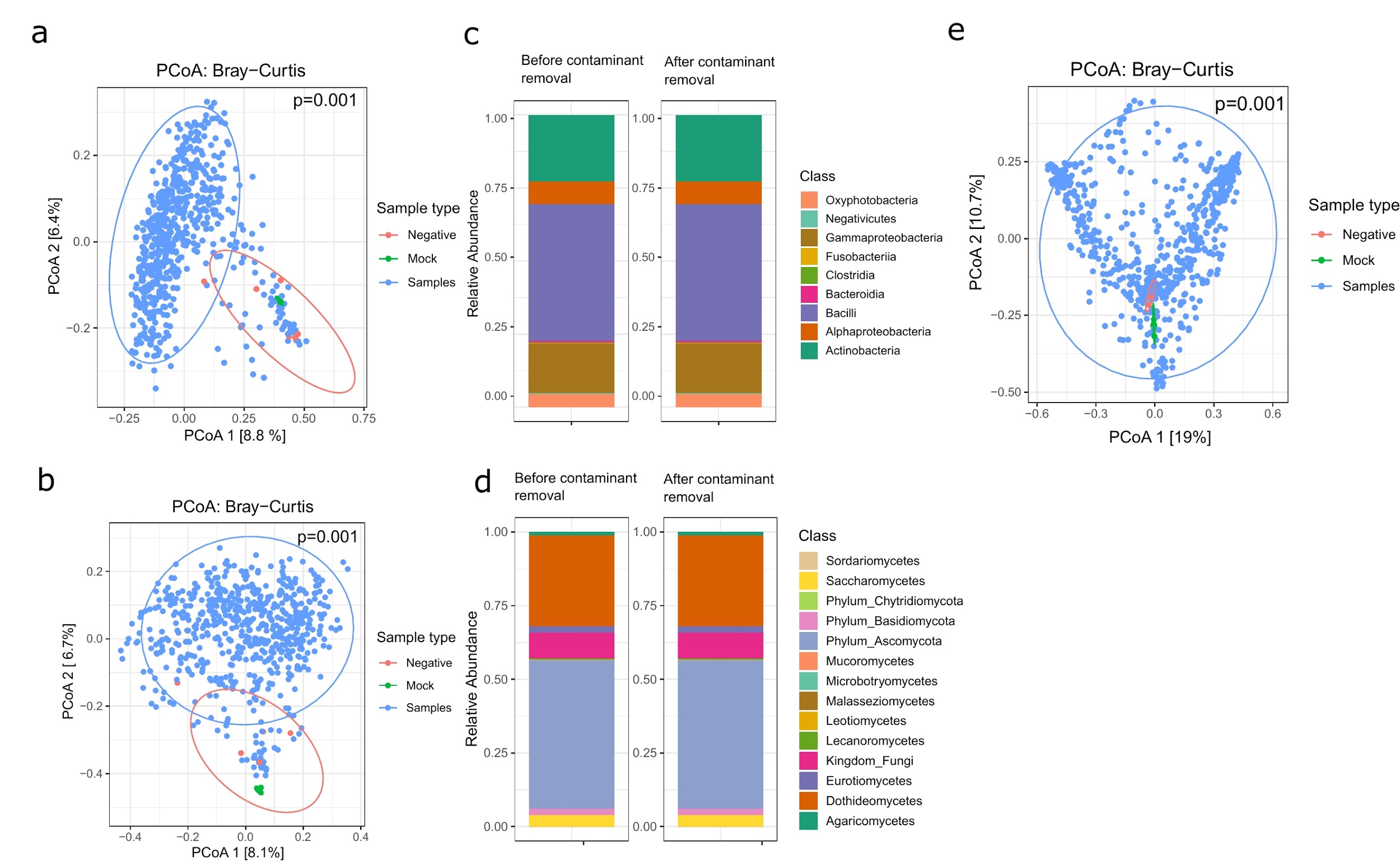


**Fig. S2. Influence of potential contaminant ASVs.** (a) β diversity by sample type prior to removal of potential contaminant ASVs using the decontam package for 16S rRNA samples. (b) β diversity by sample type prior to removal of potential contaminant ASVs using the decontam package for ITS samples. β diversity was assessed on Bray-Curtis dissimilarity and tested by PERMANOVA. There was a significant difference based on the sample type. (c) Composition of the ASVs before and after contaminant removal at class level (>1% mean relative abundance) for 16S rRNA samples. (d) Composition of the ASVs before and after contaminant removal at class level (>1% mean relative abundance) for ITS samples. (e) β diversity by sample type prior to removal of potential contaminant ASVs using the decontam package for airway samples. β diversity was assessed on Bray-Curtis dissimilarity and tested by PERMANOVA. There was a significant difference based on the sample type.


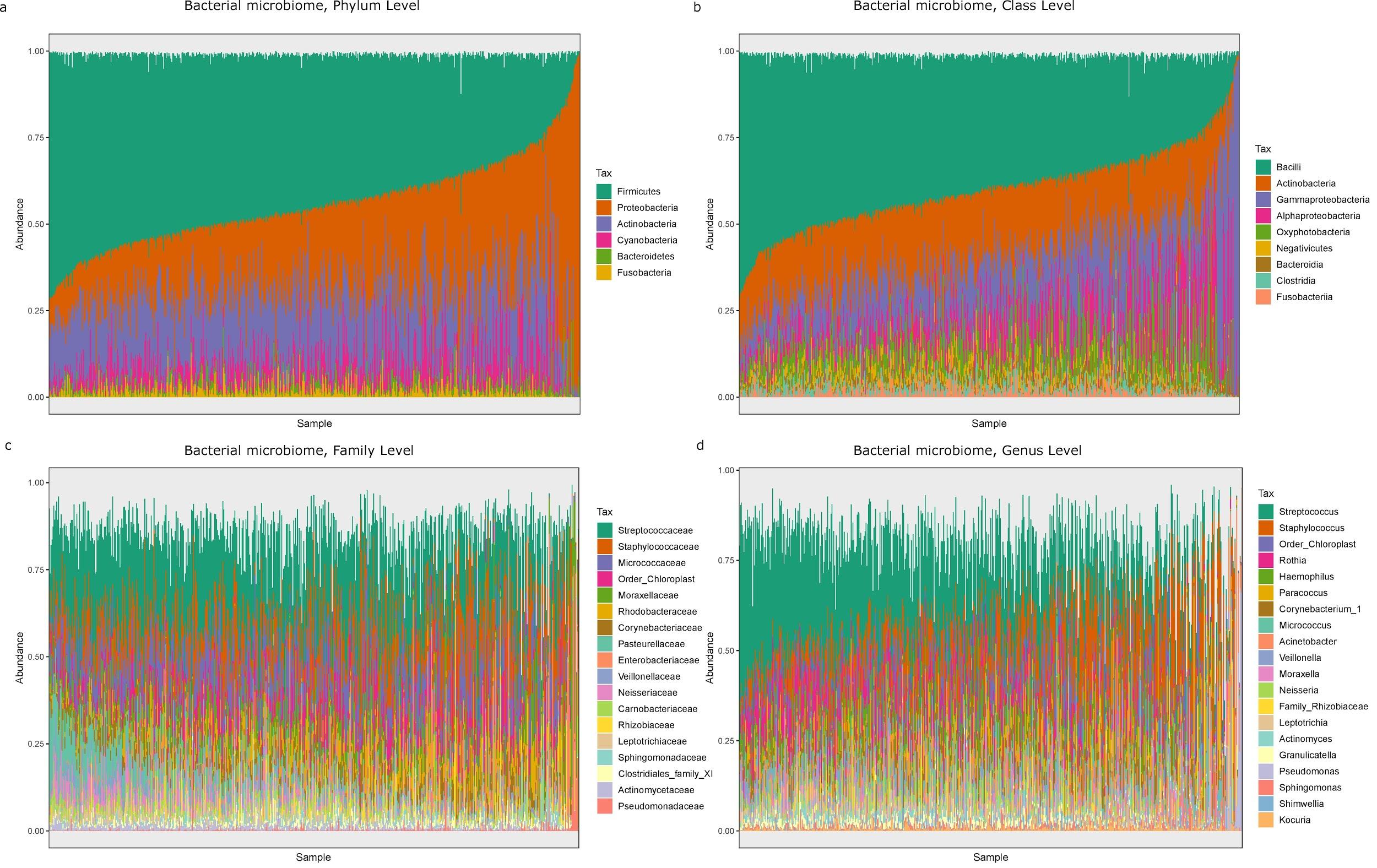


**Fig. S3.** **Bacterial microbiome by 16S rRNA gene (panels a–d) at four taxonomic levels (a) phylum (b) class (c) family, and (d) genus.** Each of the above-stacked bar plots illustrates the average relative abundance (y-axis) of the microbiota at different taxonomic levels. Taxa with a mean abundance of at least 1% across all samples are represented in colors; those with < 1% abundance are not shown. Each column represents one individual. The taxonomy for bacterial ASVs was assigned from the SILVA database.


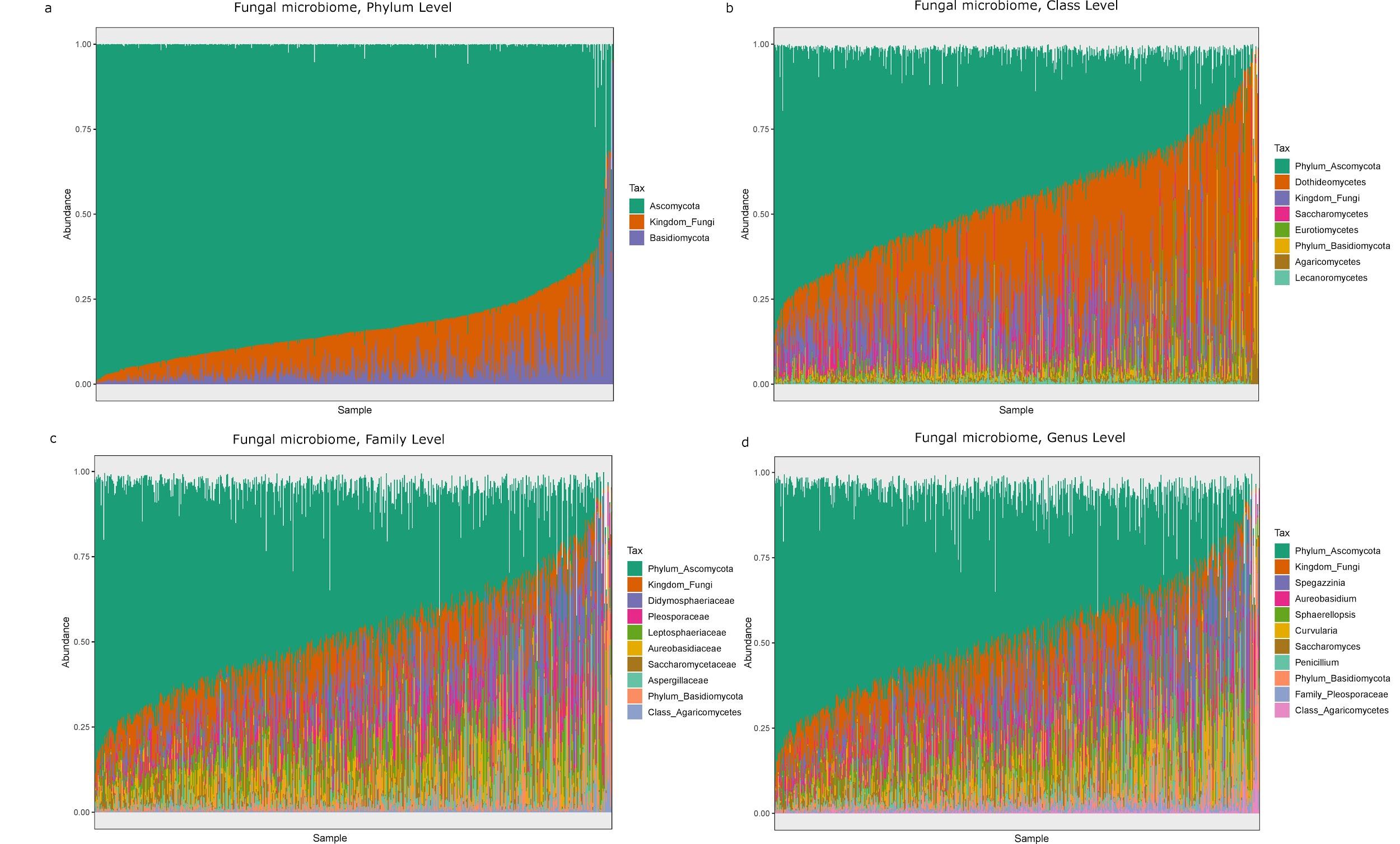


**Fig. S4.** **Fungal microbiome by ITS (panels a–d) at four taxonomic levels (a) phylum (b) class (c) family, and (d) genus.** Each of the above-stacked bar plots illustrates the average relative abundance (y-axis) of the microbiota at different taxonomic levels. Taxa with a mean abundance of at least 1% across all samples are represented in colors; those with <1% abundance are not shown. Each column represents one individual. The taxonomy for bacterial ASVs was assigned from the UNITE database.


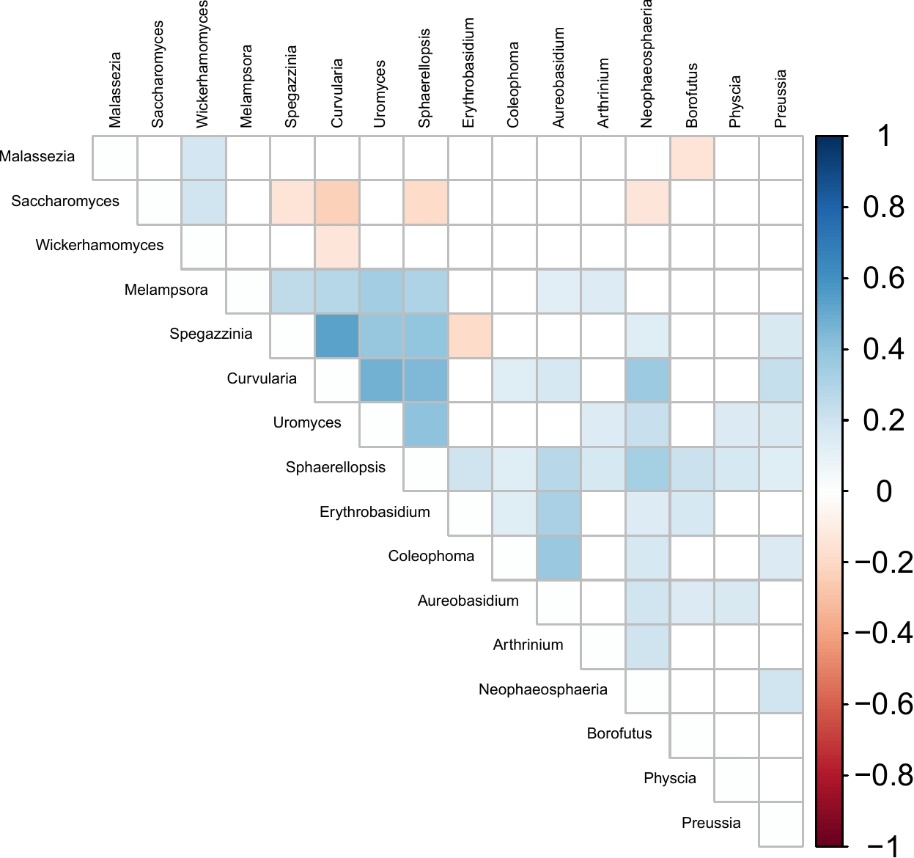


**Fig. S5.** **Spearman correlation between fungi in bed dust.** Only significant values (p<0.05 after FDR adjustment) are shown. Orange and blue represents significant negative correlations and positive correlations. Darker color represents stronger correlations.


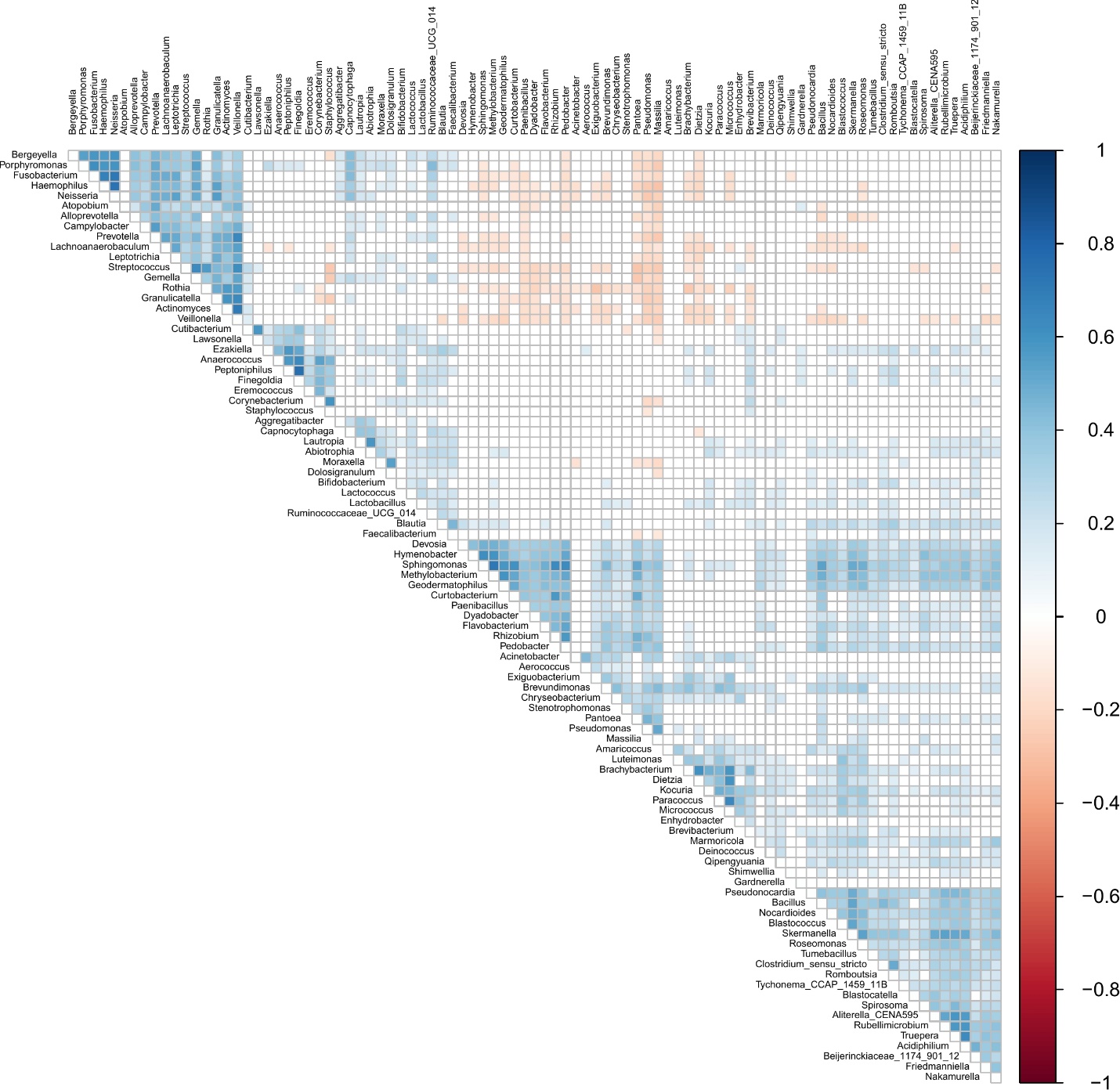


**Fig S6.** **Spearman correlation between bacteria in bed dust.** Only significant values (p<0.05 after FDR adjustment) are shown. Orange and blue represents significant negative correlations and positive correlations. Darker color represents stronger correlations.


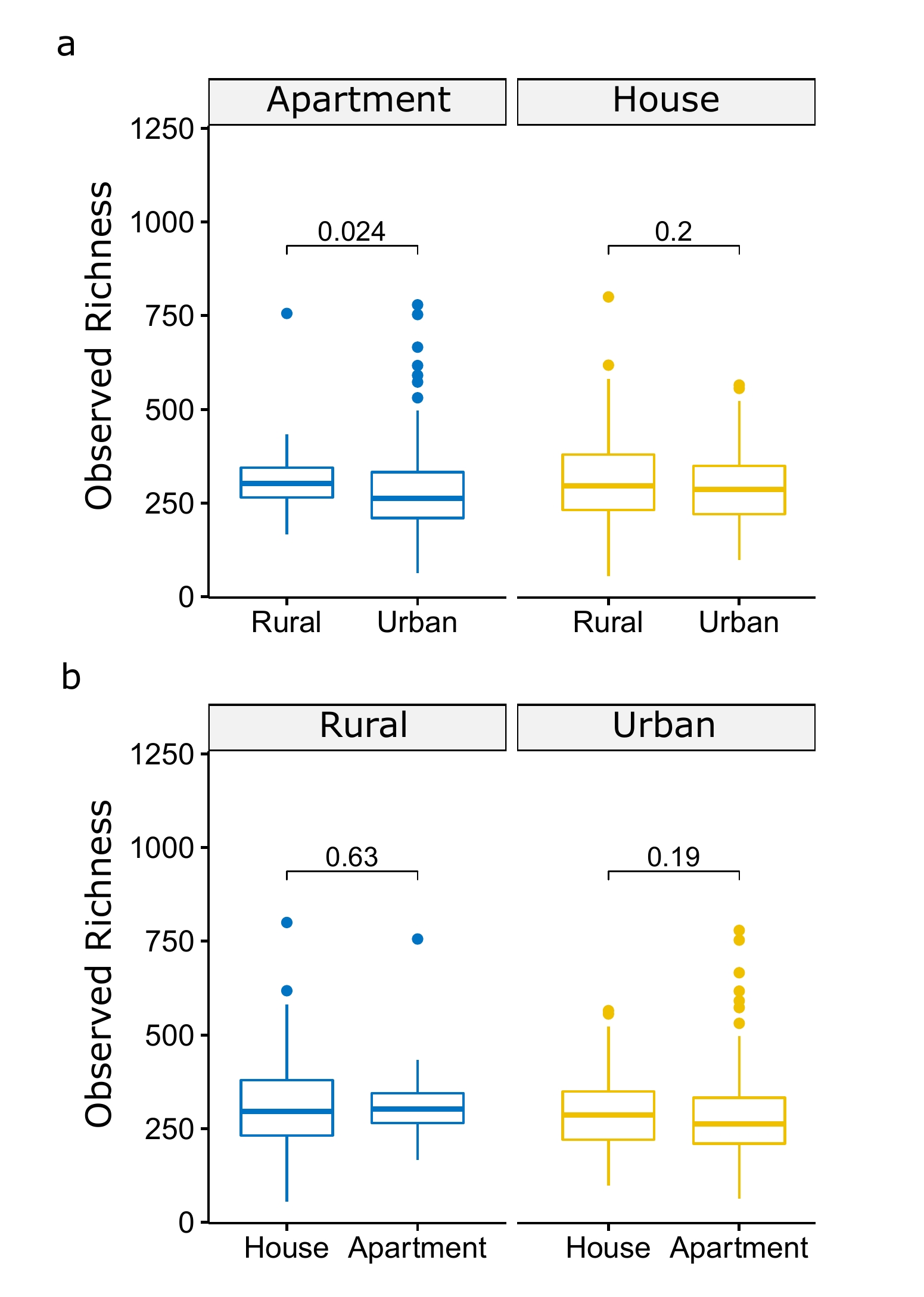


**Fig. S7. Box plots of the bacterial richness according to home type and living environment.** (a) differences between apartment and house in the rural and urban areas, (b) differences between rural and urban in the house and apartment.


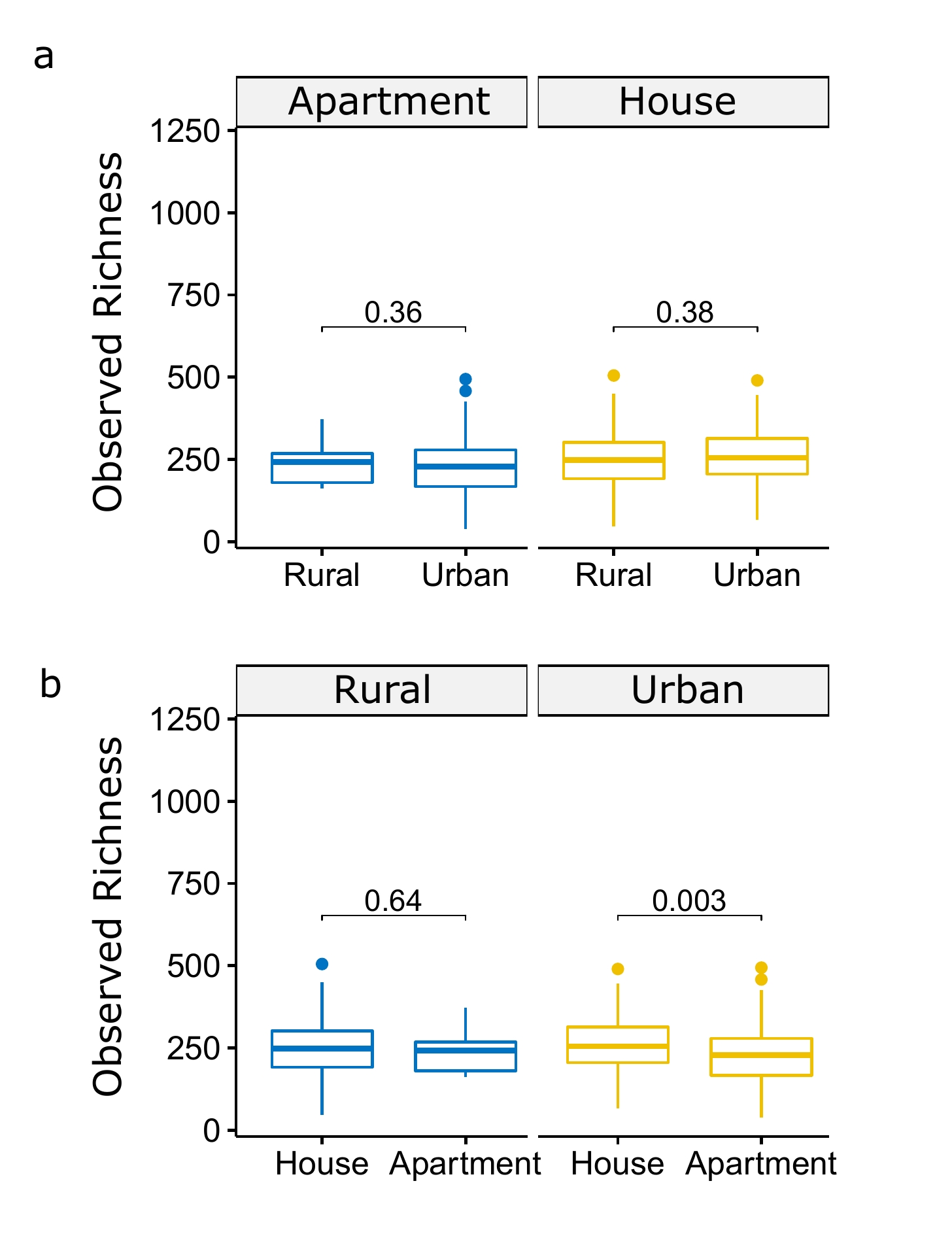


**Fig. S8. Box plots of the fungal richness according to home type and living environment.** (a) differences between apartment and house in the rural and urban areas, (b) differences between rural and urban in the house and apartment.


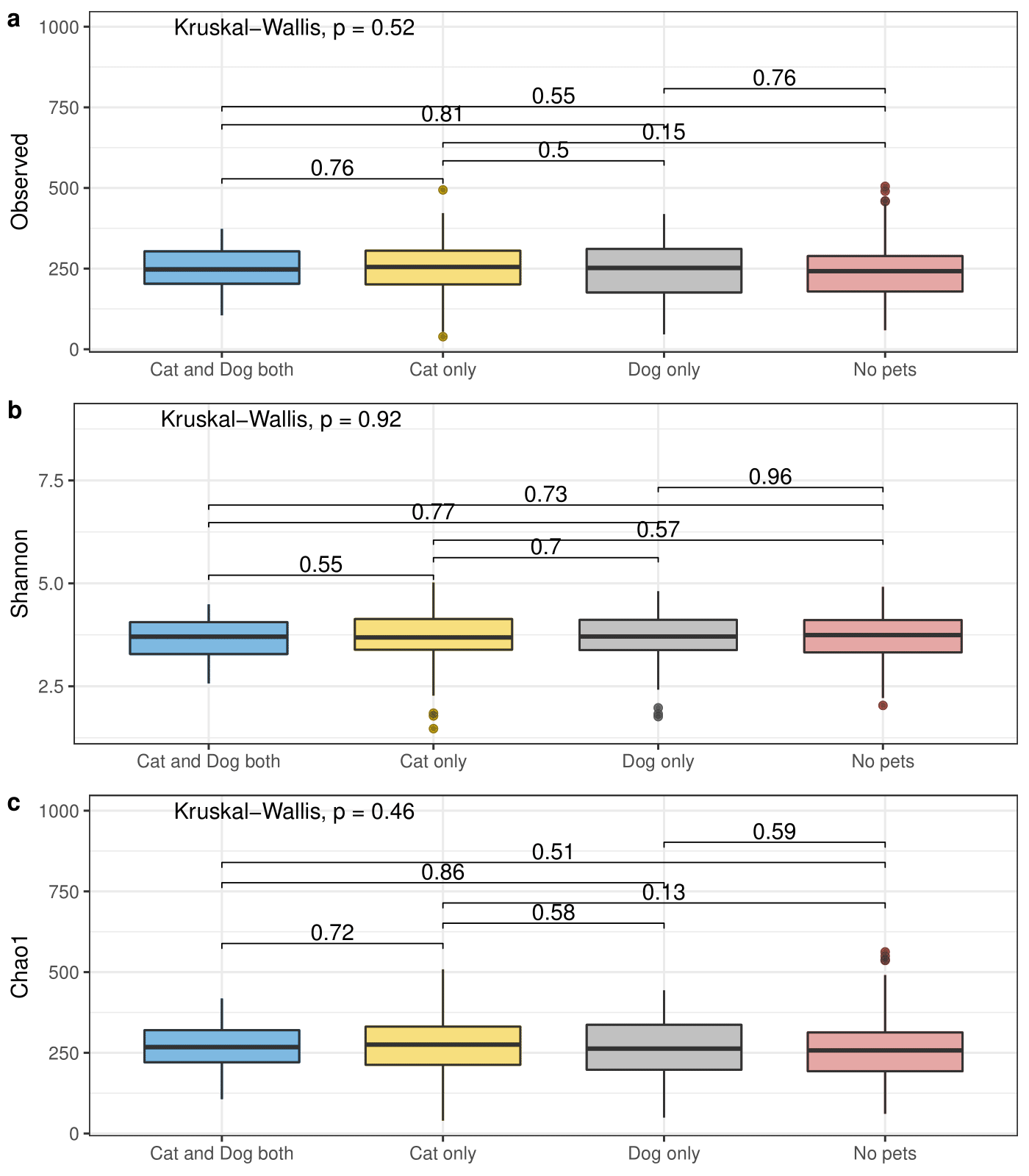


**Fig. S9. Box plots of fungal alpha-diversity according to pet ownership.** Box plots of the three diversity metrics (a) Observed, (b) Shannon diversity, and (d) Chao1 diversity.


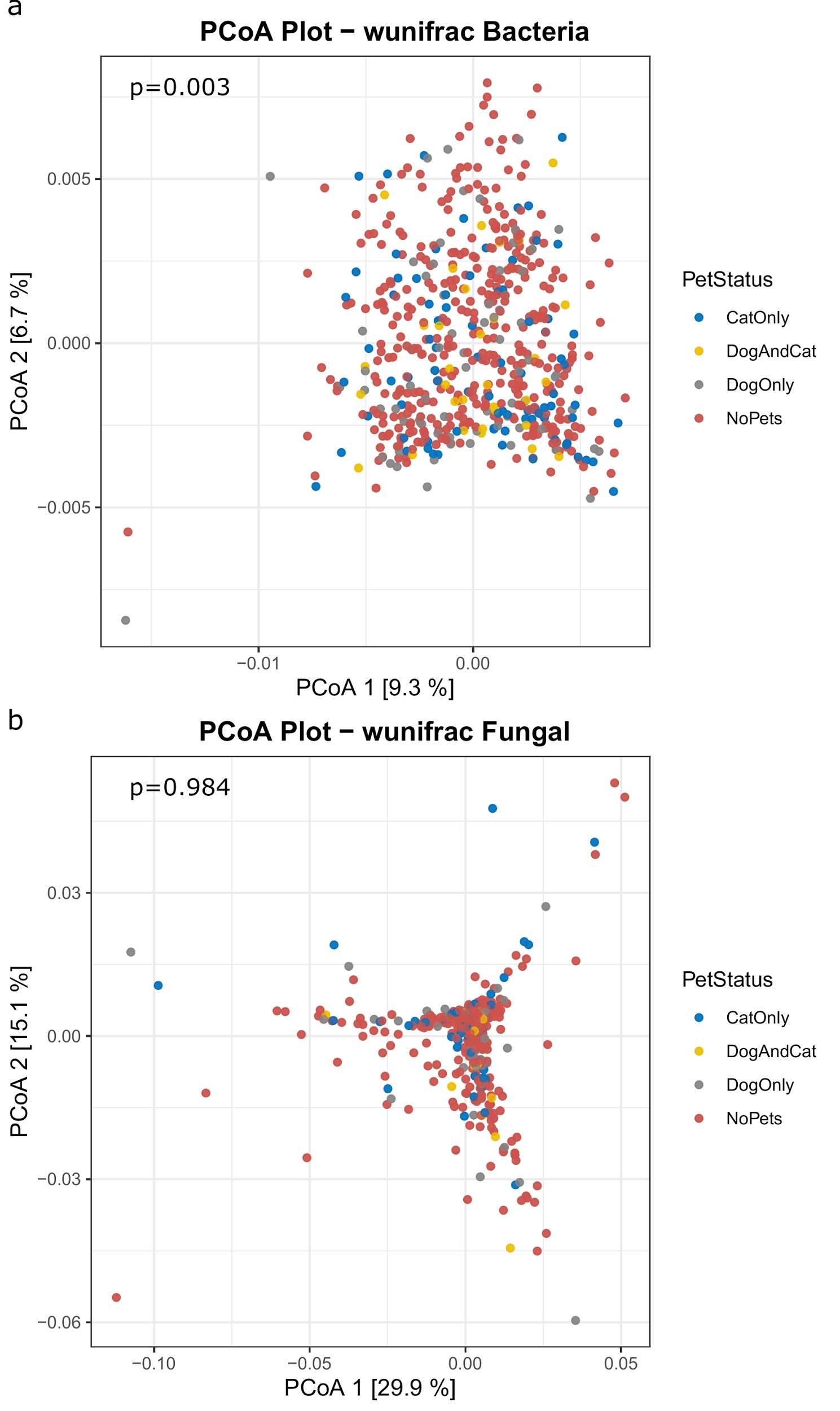


**Fig. S10.** **PCoA plot of (a) bacterial and (b) fungal community composition based on weighted Unifrac distance.** PCoA plot showing the relationship among samples belongs to a home that owns a pet.


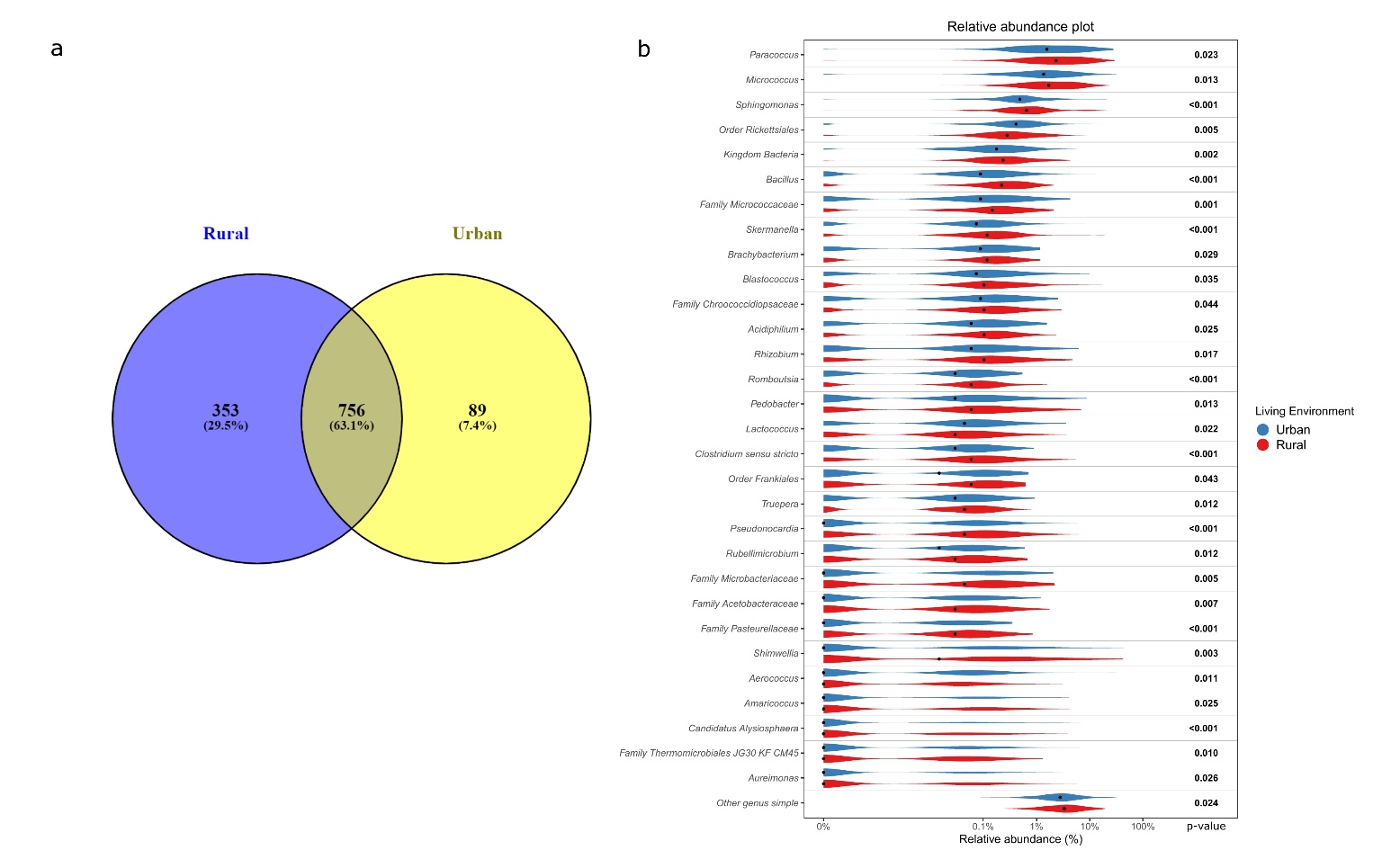


**Fig. S11. Differential abundance analysis between rural and urban living environment.** (a) Venn diagram depicting the bacterial genera constantly present in the rural and urban living environment. (b) Relative abundances in the bed dust samples associate with living environment (rural or urban). Comparison among the 30 most significantly abundant bacterial genera. P-values correspond to Wilcoxon rank-sum tests of the relative abundances, with significant values (p < 0.05) bolded with FDR correction. A pseudocount (+1e−06) was added to all abundances for the log-scale presentation. The black dots indicate median values and the abundances are colored according to the rural (red) (N = 251) or urban (blue) (N = 295) living environment.

**
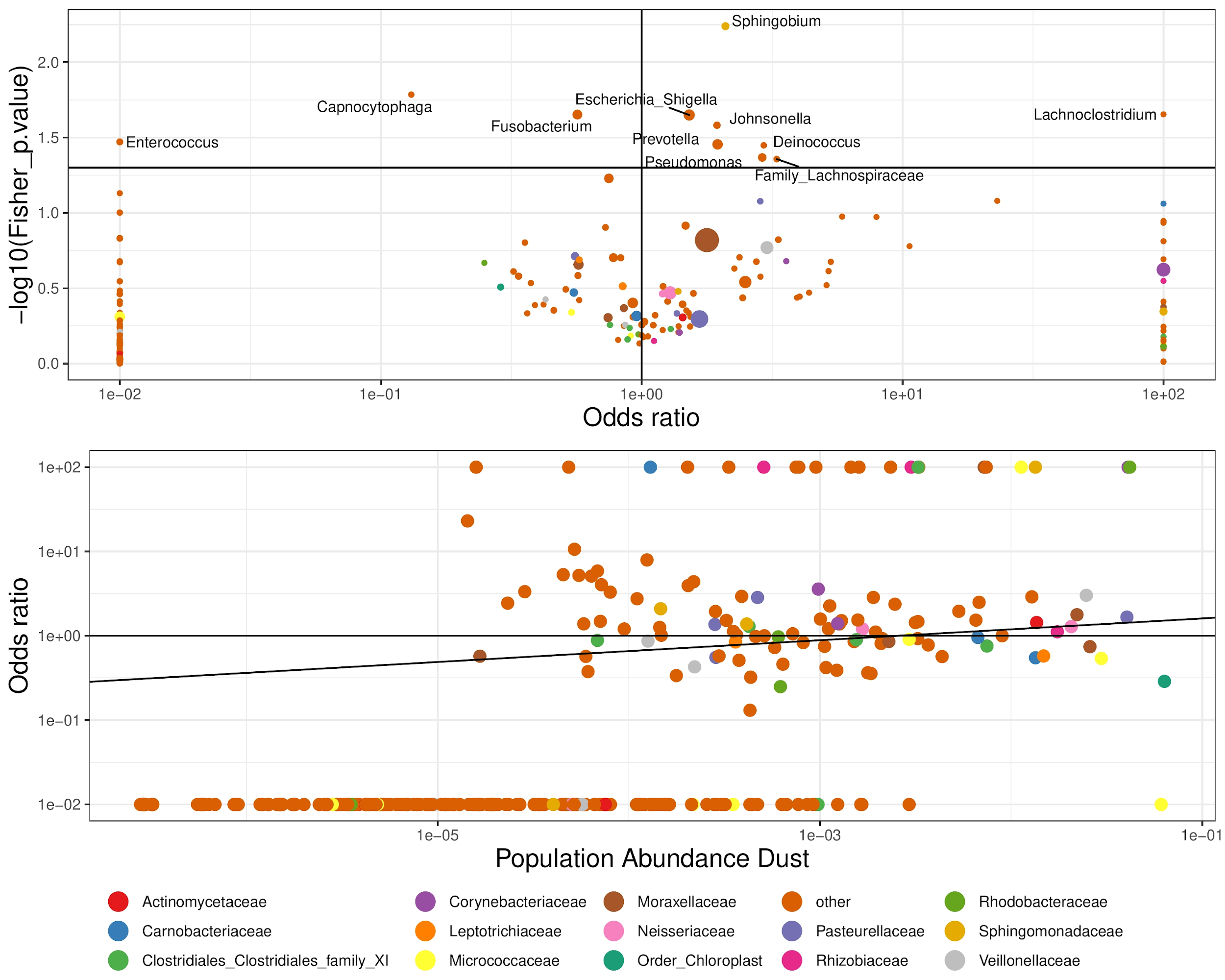
**

**Fig. S12.** **The odds for transfer of taxa (at genus level) from dust to airway microbiota of children.** Top panel shows the odds ratio (x-axis) and the strength (p-value). Of particular interest is the distribution of positive- (odds ratio>1) compared to negative odds (odds ratio<1). Lower panel shows odds ratio (y-axis) versus the population-wide dust abundance (x-axis). Odds larger (or smaller) than 100 fold are truncated to 100 (or 0.01). Colors indicate the top 15 overall most abundant taxonomic families.

**Table S1.** Characteristics of the study population. *Income level is categorize into Low (<€50,000/year), medium (€50,000–€110,000/year), high (>€110,000/year). **Education level is categorize into Low (primary school, secondary school, or college graduate), medium (tradesman or bachelor degree), high (master’s degree).

| Category | Variable | Summary statistic, n (%) |
| --- | --- | --- |
| Sex | Male | 304 (52.05) |
| Income level* | Low | 52 (8.9) |
|  | Medium | 305 (52.23) |
|  | High | 226 (38.7) |
| Type of home | House | 322 (55.14) |
|  | Apartment | 232 (39.73) |
| Type of area | Rural | 255 (43.66) |
|  | Urban | 298 (51.03) |
| Pets | Cat | 87 (14.9) |
|  | Dog | 72 (12.33) |
|  | Both | 32 (5.48) |
| Season of dust sample collection | Winter | 170 (29.11) |
|  | Spring | 159 (27.23) |
|  | Summer | 127 (21.75) |
|  | Autumn | 128 (21.92) |
| Race | Caucasian | 559 (95.72) |
| Number of siblings | None | 132 (22.6) |
|  | One | 213 (36.47) |
|  | Two or more | 120 (20.55) |
| Education level** | Low | 42 (7.2) |
|  | Medium | 371 (63.53) |
|  | High | 171 (29.28) |

**Table S2.** Bacterial abundance in bed dust samples at phylum level.

| **Phylum** | **Abundance (%)** |
| --- | --- |
| Firmicutes | 43.0538 |
| Proteobacteria | 25.6922 |
| Actinobacteria | 19.2679 |
| Cyanobacteria | 6.8913 |
| Bacteroidetes | 2.17 |
| Fusobacteria | 1.8515 |
| Kingdom_Bacteria | 0.3841 |
| Patescibacteria | 0.3163 |
| Chloroflexi | 0.1038 |
| Deinococcus-Thermus | 0.102 |
| Acidobacteria | 0.0733 |
| Epsilonbacteraeota | 0.0547 |
| Verrucomicrobia | 0.0153 |
| WPS-2 | 0.0048 |
| Gemmatimonadetes | 0.0047 |
| Planctomycetes | 0.0028 |
| Armatimonadetes | 0.0026 |
| FBP | 0.0023 |
| Tenericutes | 0.0019 |
| BRC1 | 0.0014 |
| Chlamydiae | 0.001 |
| Nitrospirae | 0.0006 |
| Synergistetes | 0.0006 |
| Spirochaetes | 0.0004 |
| Entotheonellaeota | 0.0002 |
| Fibrobacteres | 0.0002 |
| Dependentiae | 0.0001 |
| Elusimicrobia | 0.0001 |
| Halanaerobiaeota | 0.0001 |
| Kiritimatiellaeota | 0.0001 |
| Latescibacteria | 0 |
| Rokubacteria | 0 |

**Table S3. Bacterial abundance in bed dust samples at genus level.**

| **Genus** | **Abundance (%)** |
| --- | --- |
| Streptococcus | 23.6 |
| Staphylococcus | 12.4349 |
| Rothia | 6.1694 |
| Haemophilus | 4.1531 |
| Paracoccus | 4.1213 |
| Corynebacterium | 4.0865 |
| Micrococcus | 2.927 |
| Acinetobacter | 2.724 |
| Veillonella | 2.4897 |
| Moraxella | 2.2027 |
| Neisseria | 2.0933 |
| Family_Rhizobiaceae | 1.6637 |
| Leptotrichia | 1.4848 |
| Actinomyces | 1.3715 |
| Granulicatella | 1.3513 |
| Pseudomonas | 1.296 |
| Sphingomonas | 1.2832 |
| Shimwellia | 1.1242 |
| Kocuria | 1.1134 |
| Pantoea | 0.9553 |
| Anaerococcus | 0.7439 |
| Order_Lactobacillales | 0.7389 |
| Cutibacterium | 0.7277 |
| Order_Rickettsiales | 0.7142 |
| Enhydrobacter | 0.7139 |
| Gemella | 0.6778 |
| Massilia | 0.6716 |
| Dolosigranulum | 0.6542 |
| Prevotella | 0.5491 |
| Fusobacterium | 0.445 |
| Kingdom_Bacteria | 0.4104 |
| Lactobacillus | 0.3585 |
| Porphyromonas | 0.3375 |
| Finegoldia | 0.3229 |
| Bifidobacterium | 0.317 |
| Blastococcus | 0.309 |
| Pedobacter | 0.3056 |
| Bacillus | 0.3027 |
| Family_Micrococcaceae | 0.2849 |
| Gardnerella | 0.284 |
| Allorhizobium_Neorhizobium_Pararhizobium_Rhizobium | 0.2787 |
| Skermanella | 0.2492 |
| Family_Chroococcidiopsaceae | 0.2354 |
| Chryseobacterium | 0.2346 |
| Psychrobacter | 0.2269 |
| Hymenobacter | 0.2254 |
| Family_Enterobacteriaceae | 0.2115 |
| Exiguobacterium | 0.2017 |
| Methylobacterium | 0.1942 |
| Piscicoccus | 0.1876 |
| Order_Saccharimonadales | 0.186 |
| Brevundimonas | 0.1744 |
| Acidiphilium | 0.1727 |
| Brachybacterium | 0.1708 |
| Family_Neisseriaceae | 0.1666 |
| Family_Microbacteriaceae | 0.1554 |
| Peptoniphilus | 0.1539 |
| Dietzia | 0.1533 |
| Curtobacterium | 0.1528 |
| Order_Actinomycetales | 0.1474 |
| Family_Intrasporangiaceae | 0.1466 |
| Aliterella_CENA595 | 0.1435 |
| Brevibacterium | 0.1435 |
| Flavobacterium | 0.1419 |
| Serratia | 0.1364 |
| Lactococcus | 0.1339 |
| Clostridium_sensu_stricto | 0.1268 |
| Family_Saccharimonadaceae | 0.1241 |
| Lawsonella | 0.1212 |
| Roseomonas | 0.1209 |
| Pseudonocardia | 0.1198 |
| Ruminococcaceae_UCG_014 | 0.1187 |
| Atopobium | 0.1112 |
| Alloprevotella | 0.1075 |
| Aerococcus | 0.1068 |
| Ezakiella | 0.103 |
| Family_Corynebacteriaceae | 0.1004 |
| Romboutsia | 0.0916 |
| Amaricoccus | 0.0884 |
| Order_Frankiales | 0.0883 |
| Anoxybacillus | 0.0828 |
| Bergeyella | 0.0824 |
| Family_Acetobacteraceae | 0.0756 |
| Candidatus_Alysiosphaera | 0.0732 |
| Pseudoxanthomonas | 0.0732 |
| Truepera | 0.071 |
| Nocardioides | 0.0692 |
| Abiotrophia | 0.0665 |
| Janthinobacterium | 0.063 |
| Lautropia | 0.0627 |
| Beijerinckiaceae_1174_901_12 | 0.0623 |
| Luteimonas | 0.0622 |
| Carnobacterium | 0.0589 |
| Rubellimicrobium | 0.0589 |
| Family_Sphingomonadaceae | 0.0583 |
| Family_Thermomicrobiales_JG30_KF_CM45 | 0.0583 |
| Aureimonas | 0.058 |
| Campylobacter | 0.0579 |
| Family_Rhodobacteraceae | 0.0535 |
| Enterobacter | 0.0526 |
| Lachnoanaerobaculum | 0.0515 |
| Tychonema_CCAP_1459_11B | 0.0476 |
| Sarcina | 0.0468 |
| Family_Pasteurellaceae | 0.0462 |
| Kineococcus | 0.0457 |
| Faecalibacterium | 0.0455 |
| Empedobacter | 0.0454 |
| Stenotrophomonas | 0.0442 |
| Dyadobacter | 0.0441 |
| Helcococcus | 0.0436 |
| Capnocytophaga | 0.0433 |
| Nakamurella | 0.0426 |
| Sanguibacter | 0.0416 |
| Geodermatophilus | 0.0413 |
| Devosia | 0.0402 |
| Marmoricola | 0.0401 |
| Turicella | 0.04 |
| Alloscardovia | 0.0398 |
| Blautia | 0.0382 |
| Streptobacillus | 0.038 |
| Erwinia | 0.0377 |
| Novosphingobium | 0.0376 |
| Deinococcus | 0.0374 |
| Microvirga | 0.0363 |
| Friedmanniella | 0.0356 |
| Altererythrobacter | 0.0348 |
| Eremococcus | 0.0342 |
| Escherichia_Shigella | 0.0342 |
| Family_Burkholderiaceae | 0.0333 |
| Macrococcus | 0.0332 |
| Class_Actinobacteria | 0.033 |
| Tumebacillus | 0.033 |
| Gordonia | 0.0327 |
| Arthrobacter | 0.0325 |
| Tabrizicola | 0.0321 |
| Cellulomonas | 0.0319 |
| Qipengyuania | 0.0318 |
| Family_Micromonosporaceae | 0.03 |
| Actinobacillus | 0.0283 |
| Aggregatibacter | 0.0283 |
| Johnsonella | 0.0278 |
| Paenibacillus | 0.0275 |
| Mucilaginibacter | 0.0274 |
| Dialister | 0.0273 |
| Actinoplanes | 0.0272 |
| Terrisporobacter | 0.0262 |
| Turicibacter | 0.0252 |
| Sporosarcina | 0.0246 |
| Variovorax | 0.0246 |
| Spirosoma | 0.0244 |
| Ornithinimicrobium | 0.0236 |
| Sphingobacterium | 0.0235 |
| Facklamia | 0.0222 |
| Modestobacter | 0.0222 |
| Agathobacter | 0.0214 |
| Microbacterium | 0.0212 |
| Blastocatella | 0.0211 |
| Glutamicibacter | 0.0211 |
| Aeromicrobium | 0.0209 |
| Rathayibacter | 0.0207 |
| Dermabacter | 0.0205 |
| Order_Micrococcales | 0.0202 |
| Endobacter | 0.0195 |
| Kytococcus | 0.0187 |
| Rhodococcus | 0.0185 |
| Fusicatenibacter | 0.0184 |
| Phenylobacterium | 0.0181 |
| Actinomycetospora | 0.0178 |
| Family_Chitinophagaceae | 0.0171 |
| Family_Beutenbergiaceae | 0.0169 |
| Nostoc_PCC_73102 | 0.0169 |
| Duganella | 0.0166 |
| Nosocomiicoccus | 0.0162 |
| _Ruminococcus_gnavus_group | 0.016 |
| Shuttleworthia | 0.0159 |
| Adhaeribacter | 0.0155 |
| Enterococcus | 0.0152 |
| Order_Absconditabacteriales_SR1_ | 0.0152 |
| Class_Alphaproteobacteria | 0.0147 |
| Mobilicoccus | 0.0147 |
| Ilumatobacter | 0.0146 |
| Chryseomicrobium | 0.0145 |
| Mycobacterium | 0.0141 |
| Falsirhodobacter | 0.0139 |
| Oribacterium | 0.0136 |
| Scytonema_UTEX_2349 | 0.0132 |
| Sphingobium | 0.0132 |
| Family_Planococcaceae | 0.0131 |
| Dermacoccus | 0.0129 |
| Nesterenkonia | 0.0128 |
| Family_Solirubrobacterales_67_14 | 0.0125 |
| Noviherbaspirillum | 0.0125 |
| Saccharopolyspora | 0.0125 |
| Alloiococcus | 0.0124 |
| Pseudoclavibacter | 0.0124 |
| Murdochiella | 0.0121 |
| Salinicoccus | 0.0121 |
| Peptostreptococcus | 0.012 |
| Megasphaera | 0.0119 |
| Bosea | 0.0118 |
| Family_Peptostreptococcaceae | 0.0118 |
| Quadrisphaera | 0.0118 |
| Subdoligranulum | 0.0118 |
| Jeotgalicoccus | 0.0117 |
| Bryocella | 0.0116 |
| Family_Anaerolineae_SBR1031_A4b | 0.0114 |
| Caulobacter | 0.0113 |
| Family_Dermacoccaceae | 0.0112 |
| Fastidiosipila | 0.0112 |
| Intestinibacter | 0.011 |
| Kineosporia | 0.0109 |
| Family_Blastocatellaceae | 0.0106 |
| Rickettsia | 0.0106 |
| Staphylococcaceae_S31 | 0.0102 |
| Chamaesiphon_PCC_7430 | 0.01 |
| Erythrobacter | 0.0099 |
| Collinsella | 0.0097 |
| Microlunatus | 0.0097 |
| Family_Dermatophilaceae | 0.0096 |
| Family_Ilumatobacteraceae | 0.0094 |
| Lysobacter | 0.0093 |
| Sneathia | 0.0092 |
| Paeniclostridium | 0.0091 |
| Cellulosimicrobium | 0.009 |
| Granulicella | 0.009 |
| Kosakonia | 0.009 |
| Gaiella | 0.0089 |
| Leuconostoc | 0.0089 |
| Clavibacter | 0.0086 |
| Craurococcus | 0.0083 |
| Order_Nostocales | 0.0083 |
| Calothrix_PCC_6303 | 0.0082 |
| Mobiluncus | 0.0081 |
| Ottowia | 0.0081 |
| Pleurocapsa_PCC_7319 | 0.0081 |
| Klebsiella | 0.0077 |
| Solirubrobacter | 0.0077 |
| Family_Lachnospiraceae | 0.0076 |
| Ruminococcus | 0.0075 |
| Order_Bacillales | 0.0074 |
| Varibaculum | 0.0074 |
| Alkanindiges | 0.0071 |
| Brevibacillus | 0.0071 |
| Family_Beijerinckiaceae | 0.007 |
| Parvimonas | 0.007 |
| Pseudoalteromonas | 0.0069 |
| Stomatobaculum | 0.0069 |
| Planococcus | 0.0068 |
| Solibacillus | 0.0068 |
| Family_Caulobacteraceae | 0.0067 |
| Negativicoccus | 0.0067 |
| Class_Chloroflexi_KD4_96 | 0.0066 |
| Ignavigranum | 0.0063 |
| Lysinibacillus | 0.0063 |
| Xanthomonas | 0.0063 |
| Bacteroides | 0.0062 |
| Bryobacter | 0.0061 |
| Solobacterium | 0.0061 |
| Order_Gaiellales | 0.006 |
| Phylum_Proteobacteria | 0.006 |
| Salegentibacter_sp_PR54_18 | 0.0059 |
| Demequina | 0.0058 |
| Order_Microtrichales | 0.0058 |
| Anaerostipes | 0.0057 |
| Eggerthellaceae_DNF00809 | 0.0057 |
| Akkermansia | 0.0056 |
| Haematobacter | 0.0056 |
| Kingella | 0.0056 |
| Selenomonas | 0.0056 |
| Sinobaca | 0.0054 |
| Thermoactinomyces | 0.0054 |
| Weissella | 0.0054 |
| Family_Myxococcales_BIrii41 | 0.0053 |
| Planomicrobium | 0.0053 |
| Rhodocytophaga | 0.0053 |
| Paeniglutamicibacter | 0.0052 |
| Clostridiales_Clostridiales_family_XI_W5053 | 0.0051 |
| Gallicola | 0.0051 |
| Phylum_WPS-2 | 0.0051 |
| Brochothrix | 0.005 |
| Geobacillus | 0.005 |
| Class_Gammaproteobacteria | 0.0049 |
| Family_Solirubrobacteraceae | 0.0049 |
| Geminicoccus | 0.0049 |
| Terriglobus | 0.0049 |
| Cardiobacterium | 0.0048 |
| Granulicatella_sp_BB_11 | 0.0048 |
| Paraburkholderia_tropica | 0.0048 |
| Rheinheimera | 0.0048 |
| Rubrobacter | 0.0048 |
| Clostridiales_Clostridiales_family_XIII_S5_A14a | 0.0047 |
| Family_Archangiaceae | 0.0047 |
| Iamia | 0.0047 |
| Streptomyces | 0.0047 |
| Acidovorax | 0.0046 |
| Class_Acidobacteria_subgrp_6 | 0.0046 |
| Paludibacteraceae_F0058 | 0.0046 |
| Pseudoglutamicibacter | 0.0045 |
| Luteolibacter | 0.0044 |
| Family_Kallotenuales_AKIW781 | 0.0043 |
| Family_Kineosporiaceae | 0.0043 |
| Pontibacter | 0.0043 |
| _Eubacterium_eligens_group | 0.0042 |
| Family_Microscillaceae | 0.0042 |
| Family_Weeksellaceae | 0.0041 |
| Photobacterium | 0.0041 |
| Verticia | 0.0041 |
| Chroococcidiopsis_SAG_2023 | 0.004 |
| Candidatus_Saccharimonas | 0.0039 |
| Family_Pseudomonadaceae | 0.0039 |
| Sphingorhabdus | 0.0039 |
| Eikenella | 0.0038 |
| Herpetosiphon | 0.0038 |
| Rubritepida | 0.0038 |
| Clostridiisalibacter | 0.0037 |
| Rhizobacter | 0.0037 |
| Family_Sandaracinaceae | 0.0036 |
| Rummeliibacillus | 0.0036 |
| Laceyella | 0.0035 |
| _Ruminococcus_torques_group | 0.0034 |
| Aeromonas | 0.0034 |
| Amnibacterium | 0.0034 |
| Class_Bacilli | 0.0034 |
| Defluviicoccus | 0.0034 |
| Simonsiella | 0.0034 |
| Hydrogenispora | 0.0033 |
| _Eubacterium_hallii_group | 0.0032 |
| Dokdonella | 0.0032 |
| Family_Nostocaceae | 0.0032 |
| Family_Roseiflexaceae | 0.0032 |
| Family_Xanthomonadaceae | 0.0032 |
| Saccharibacillus | 0.0032 |
| Aestuariimicrobium | 0.0031 |
| Cellvibrio | 0.0031 |
| Chloroflexus | 0.003 |
| Family_Ardenticatenaceae | 0.003 |
| Ferruginibacter | 0.0029 |
| Garicola | 0.0029 |
| Chryseolinea | 0.0028 |
| Family_Devosiaceae | 0.0028 |
| Psychrobacillus | 0.0028 |
| Sporocytophaga | 0.0028 |
| Actinomycetaceae_F0332 | 0.0027 |
| Comamonas | 0.0027 |
| Order_Micavibrionales | 0.0027 |
| Paludibaculum | 0.0027 |
| Wolbachia | 0.0027 |
| Pseudofulvimonas | 0.0026 |
| Candidatus_Cardinium | 0.0025 |
| Enteractinococcus | 0.0025 |
| Family_Bacillaceae | 0.0025 |
| Nubsella | 0.0025 |
| Phylum_FBP | 0.0025 |
| Actinotignum | 0.0024 |
| Family_Myxococcales_P3OB_42 | 0.0024 |
| Phylum_Firmicutes | 0.0024 |
| Bdellovibrio | 0.0023 |
| Lachnospiraceae_ND3007 | 0.0023 |
| Ochrobactrum | 0.0023 |
| Phaselicystis | 0.0023 |
| Citrobacter | 0.0022 |
| Pelagibacterium | 0.0022 |
| Thermomonas | 0.0022 |
| Cohnella | 0.0021 |
| Cruoricaptor | 0.0021 |
| Family_Pseudonocardiaceae | 0.0021 |
| Gemmatirosa | 0.0021 |
| Halomonas | 0.0021 |
| Izhakiella | 0.0021 |
| Kallotenue | 0.0021 |
| Xylophilus | 0.0021 |
| _Eubacterium_brachy_group | 0.002 |
| Catonella | 0.002 |
| Diaphorobacter | 0.002 |
| Family_Sphingobacteriaceae | 0.002 |
| Ilumatobacteraceae_CL500_29 | 0.002 |
| Rickettsiella | 0.002 |
| Aliicoccus | 0.0019 |
| Burkholderia_Caballeronia_Paraburkholderia | 0.0019 |
| Christensenellaceae_R_7 | 0.0019 |
| Family_Carnobacteriaceae | 0.0019 |
| Family_Lactobacillales_P5D1_392 | 0.0019 |
| Family_marine_metagenome | 0.0019 |
| Family_Propionibacteriaceae | 0.0019 |
| Family_Ruminococcaceae | 0.0019 |
| Lachnospira | 0.0019 |
| Polymorphobacter | 0.0019 |
| Rhizorhapis | 0.0019 |
| Segetibacter | 0.0019 |
| Sphingopyxis | 0.0019 |
| Tessaracoccus | 0.0019 |
| Williamsia | 0.0019 |
| Wilmottia_Ant_Ph58 | 0.0019 |
| Class_Acidimicrobiia | 0.0018 |
| Erysipelatoclostridium | 0.0018 |
| Family_Cytophagales_MWH_CFBk5 | 0.0018 |
| Family_Fimbriimonadaceae | 0.0018 |
| Globicatella | 0.0018 |
| Haliangium | 0.0018 |
| Marinobacter | 0.0018 |
| Ruminiclostridium | 0.0018 |
| Shinella | 0.0018 |
| _Eubacterium_nodatum_group | 0.0017 |
| Cellulosilyticum | 0.0017 |
| Erysipelotrichaceae_UCG_003 | 0.0017 |
| Family_Aerococcaceae | 0.0017 |
| Family_Moraxellaceae | 0.0017 |
| Jatrophihabitans | 0.0017 |
| Virgibacillus | 0.0017 |
| Actinomadura | 0.0016 |
| Algoriphagus | 0.0016 |
| Atopostipes | 0.0016 |
| Bavariicoccus | 0.0016 |
| Chthoniobacter | 0.0016 |
| Chthoniobacteraceae_LD29 | 0.0016 |
| Frondihabitans | 0.0016 |
| Gillisia | 0.0016 |
| Jeotgalibaca | 0.0016 |
| Lachnoclostridium | 0.0016 |
| Leucobacter | 0.0016 |
| Order_Actinobacteria_PeM15 | 0.0016 |
| Pyrinomonadaceae_RB41 | 0.0016 |
| Sphingomonadaceae_SD04E11 | 0.0016 |
| TM7_phylum_sp_canine_oral_taxon_250 | 0.0016 |
| _Eubacterium_coprostanoligenes_group | 0.0015 |
| Aetherobacter | 0.0015 |
| Arcticibacter | 0.0015 |
| Luteibacter | 0.0015 |
| Mogibacterium | 0.0015 |
| Pseudokineococcus | 0.0015 |
| Psychroflexus | 0.0015 |
| Ruminococcaceae_UCG_002 | 0.0015 |
| Salinivibrio | 0.0015 |
| Streptomycetaceae_E1B_B3_114 | 0.0015 |
| Acidothermus | 0.0014 |
| Candidatus_Xiphinematobacter | 0.0014 |
| Chloroflexaceae_FFCH7168 | 0.0014 |
| Class_Gracilibacteria | 0.0014 |
| Dorea | 0.0014 |
| Howardella | 0.0014 |
| Nannocystis | 0.0014 |
| Phylum_BRC1 | 0.0014 |
| Salana | 0.0014 |
| Streptococcus_sp_oral_clone_ASCB12 | 0.0014 |
| Vibrio | 0.0014 |
| Antricoccus | 0.0013 |
| Candidatus_Captivus | 0.0013 |
| Family_Caldilineaceae | 0.0013 |
| Family_Enterococcaceae | 0.0013 |
| Fibrella | 0.0013 |
| Helcobacillus | 0.0013 |
| Myxococcus | 0.0013 |
| Oligoflexus | 0.0013 |
| Order_Actinomarinales | 0.0013 |
| Order_Rhizobiales | 0.0013 |
| Oxyphotobacteria_Incertae_Sedis | 0.0013 |
| Weeksella | 0.0013 |
| Arsenicicoccus | 0.0012 |
| Candidatus_Paracaedibacter | 0.0012 |
| Cnuella | 0.0012 |
| Family_Diplorickettsiaceae | 0.0012 |
| Family_Rickettsiaceae | 0.0012 |
| Gemmobacter | 0.0012 |
| Microtrichaceae_IMCC26207 | 0.0012 |
| Pasteurella | 0.0012 |
| Phascolarctobacterium | 0.0012 |
| Phormidium_CYN64 | 0.0012 |
| Pseudorhodoferax | 0.0012 |
| Roseburia | 0.0012 |
| Actinobaculum | 0.0011 |
| Alishewanella | 0.0011 |
| Auritidibacter | 0.0011 |
| Class_Chloroflexi_Gitt_GS_136 | 0.0011 |
| Conexibacter | 0.0011 |
| Coprococcus | 0.0011 |
| Family_Balneolaceae | 0.0011 |
| Family_Cellvibrionaceae | 0.0011 |
| Family_Myxococcaceae | 0.0011 |
| Family_Thermomicrobiales_AKYG1722 | 0.0011 |
| Lachnospiraceae_NK4A136 | 0.0011 |
| Leptolyngbya_ANT_L52_2 | 0.0011 |
| Micromonospora | 0.0011 |
| Nodosilinea_PCC_7104 | 0.0011 |
| Oceanivirga | 0.0011 |
| Oceanobacillus | 0.0011 |
| Order_Acidimicrobiia_IMCC26256 | 0.0011 |
| Peptococcus | 0.0011 |
| Phylum_Actinobacteria | 0.0011 |
| Planifilum | 0.0011 |
| Pseudopropionibacterium | 0.0011 |
| Rubrivirga | 0.0011 |
| Ruminococcaceae_UCG_013 | 0.0011 |
| Tissierella | 0.0011 |
| Tsukamurella | 0.0011 |
| Vulcaniibacterium | 0.0011 |
| Achromobacter | 0.001 |
| Actinotalea | 0.001 |
| Agrococcus | 0.001 |
| Aliihoeflea | 0.001 |
| Dapisostemonum_CCIBt_3536 | 0.001 |
| Edaphobacter | 0.001 |
| endosymbionts8 | 0.001 |
| Family_Acidobacteriaceae_subgrp_1 | 0.001 |
| Family_Gemmatimonadaceae | 0.001 |
| Family_Longimicrobiaceae | 0.001 |
| Frederiksenia | 0.001 |
| Lachnospiraceae_UCG_007 | 0.001 |
| Olivibacter | 0.001 |
| Parapedobacter | 0.001 |
| Peredibacter | 0.001 |
| Pseudactinotalea | 0.001 |
| Raoultella | 0.001 |
| Salinimicrobium | 0.001 |
| Terrimonas | 0.001 |
| Thauera | 0.001 |
| Alkalibacterium | 0.0009 |
| Asticcacaulis | 0.0009 |
| Candidatus_Uzinura | 0.0009 |
| Family_Saccharimonadales_soil_bacterium_WF55 | 0.0009 |
| Filifactor | 0.0009 |
| Flavisolibacter | 0.0009 |
| Marinilactibacillus | 0.0009 |
| Melittangium | 0.0009 |
| Microcoleus_SAG_1449_1a | 0.0009 |
| Nitrosospira | 0.0009 |
| Order_Armatimonadales | 0.0009 |
| Order_Clostridiales | 0.0009 |
| Psychroglaciecola | 0.0009 |
| Vagococcus | 0.0009 |
| Anaerolineae_SBR1031_A4b_OLB13 | 0.0008 |
| Class_Actinobacteria_MB_A2_108 | 0.0008 |
| Family_Veillonellaceae | 0.0008 |
| Fonticella | 0.0008 |
| Holdemanella | 0.0008 |
| Isoptericola | 0.0008 |
| Kurthia | 0.0008 |
| Leptolyngbya_Es_Yyy1000 | 0.0008 |
| Mycoplasma | 0.0008 |
| Olsenella | 0.0008 |
| Order_Betaproteobacteriales | 0.0008 |
| Order_Corynebacteriales | 0.0008 |
| Order_Myxococcales | 0.0008 |
| Paenisporosarcina | 0.0008 |
| Pajaroellobacter | 0.0008 |
| Peptoclostridium | 0.0008 |
| Planktothrix_NIVA_CYA_15 | 0.0008 |
| Polaromonas | 0.0008 |
| Shewanella | 0.0008 |
| Sorangium | 0.0008 |
| Tolypothrix_PCC_7601 | 0.0008 |
| Trichococcus | 0.0008 |
| _Ruminococcus_gauvreauii_group | 0.0007 |
| Acidipila | 0.0007 |
| Actinokineospora | 0.0007 |
| Azospirillum | 0.0007 |
| Azotobacter | 0.0007 |
| Bdellovibrionaceae_OM27 | 0.0007 |
| Bhargavaea | 0.0007 |
| Blastocatellaceae_JGI_0001001_H03 | 0.0007 |
| Chalicogloea_CCALA_975 | 0.0007 |
| Class_Bacteroidia | 0.0007 |
| Corallococcus | 0.0007 |
| Cytophaga | 0.0007 |
| Delftia | 0.0007 |
| Family_Tepidisphaerales_WD2101_soil_group | 0.0007 |
| Fictibacillus | 0.0007 |
| Jonquetella | 0.0007 |
| Megamonas | 0.0007 |
| Micropruina | 0.0007 |
| Moheibacter | 0.0007 |
| Morganella | 0.0007 |
| Moryella | 0.0007 |
| Nitrospira | 0.0007 |
| Oerskovia | 0.0007 |
| Order_Gracilibacteria_JGI_0000069_P22 | 0.0007 |
| Pilimelia | 0.0007 |
| Polyangium | 0.0007 |
| Propioniciclava | 0.0007 |
| Rhodanobacter | 0.0007 |
| Rhodovarius | 0.0007 |
| Scytonema_UCFS19 | 0.0007 |
| _Eubacterium_xylanophilum_group | 0.0006 |
| Acidocella | 0.0006 |
| Aerosphaera | 0.0006 |
| Aquabacterium | 0.0006 |
| Belnapia | 0.0006 |
| Chiayiivirga | 0.0006 |
| Family_Geodermatophilaceae | 0.0006 |
| Family_Limnochordaceae | 0.0006 |
| Family_Rhizobiales_A0839 | 0.0006 |
| Family_Xanthobacteraceae | 0.0006 |
| Georgenia | 0.0006 |
| Gluconobacter | 0.0006 |
| Huanghella | 0.0006 |
| Marinococcus | 0.0006 |
| Order_Anaerolineae_SBR1031 | 0.0006 |
| Scardovia | 0.0006 |
| Taibaiella | 0.0006 |
| Thermobacillus | 0.0006 |
| Tyzzerella | 0.0006 |
| Alysiella | 0.0005 |
| Aquipuribacter | 0.0005 |
| Arcanobacterium | 0.0005 |
| Arcobacter | 0.0005 |
| Buchnera | 0.0005 |
| Candidatus_Solibacter | 0.0005 |
| Chitinophaga | 0.0005 |
| Chroococcidiopsis_CC1 | 0.0005 |
| Class_Chloroflexi_TK10 | 0.0005 |
| Class_Oxyphotobacteria | 0.0005 |
| Cloacibacterium | 0.0005 |
| Family_Anaerolineaceae | 0.0005 |
| Family_Chloroflexaceae | 0.0005 |
| Family_Cyclobacteriaceae | 0.0005 |
| Family_Cytophagaceae | 0.0005 |
| Family_Demequinaceae | 0.0005 |
| Family_Geminicoccaceae | 0.0005 |
| Family_Nocardioidaceae | 0.0005 |
| Family_Paracaedibacteraceae | 0.0005 |
| Family_Polyangiaceae | 0.0005 |
| Family_Rhizobiales_D05_2 | 0.0005 |
| Family_Rhodothermaceae | 0.0005 |
| Family_Rickettsiales_AB1 | 0.0005 |
| Family_Sporichthyaceae | 0.0005 |
| Family_Thermoactinomycetaceae | 0.0005 |
| Flavihumibacter | 0.0005 |
| Fluviicola | 0.0005 |
| Gemmatimonas | 0.0005 |
| Luteococcus | 0.0005 |
| Methylocella | 0.0005 |
| Methylotenera | 0.0005 |
| Natribacillus | 0.0005 |
| Neomicrococcus | 0.0005 |
| Nocardiopsis | 0.0005 |
| Oligella | 0.0005 |
| Order_Blastocatellia_subgrp_4_Elev_16S_573 | 0.0005 |
| Order_Gracilibacteria_bacterium_canine_oral_taxon_394 | 0.0005 |
| Order_Mollicutes_RF39 | 0.0005 |
| Parviterribacter | 0.0005 |
| Promicromonospora | 0.0005 |
| Pseudosphingobacterium | 0.0005 |
| Rhodoferax | 0.0005 |
| Rhodopseudomonas | 0.0005 |
| Ruminococcaceae_NK4A214 | 0.0005 |
| Sphingomonadaceae_MN_122_2a | 0.0005 |
| Acaricomes | 0.0004 |
| Algoriella | 0.0004 |
| Aliifodinibius | 0.0004 |
| Ammoniphilus | 0.0004 |
| Anaerolinea | 0.0004 |
| Aneurinibacillus | 0.0004 |
| Arenibacter | 0.0004 |
| Aridibacter | 0.0004 |
| Butyricicoccus | 0.0004 |
| Caedibacter | 0.0004 |
| Caldicoprobacter | 0.0004 |
| Candidatus_Chloroploca | 0.0004 |
| Candidatus_Jidaibacter | 0.0004 |
| Candidatus_Udaeobacter | 0.0004 |
| Catenibacterium | 0.0004 |
| Caulobacteraceae_PMMR1 | 0.0004 |
| Cereibacter | 0.0004 |
| Class_Blastocatellia_subgrp_4 | 0.0004 |
| Clostridioides | 0.0004 |
| Cobetia | 0.0004 |
| Dactylosporangium | 0.0004 |
| Desemzia | 0.0004 |
| Desulfosporosinus | 0.0004 |
| Domibacillus | 0.0004 |
| Erysipelothrix | 0.0004 |
| Family_Actinomycetaceae | 0.0004 |
| Family_Candidatus_Saccharibacteria_bacterium_UB2523 | 0.0004 |
| Family_Clostridiaceae_1 | 0.0004 |
| Family_Muribaculaceae | 0.0004 |
| Family_Nocardiaceae | 0.0004 |
| Family_Sphingobacteriales_env_OPS_17 | 0.0004 |
| Family_Spirosomaceae | 0.0004 |
| Family_Staphylococcaceae | 0.0004 |
| Family_Streptomycetaceae | 0.0004 |
| Family_Tepidisphaeraceae | 0.0004 |
| Flaviflexus | 0.0004 |
| Franconibacter | 0.0004 |
| Gilliamella | 0.0004 |
| Jannaschia | 0.0004 |
| Lachnospiraceae_CAG_56 | 0.0004 |
| Leptolyngbya_PCC_6306 | 0.0004 |
| Limnobacter | 0.0004 |
| Methyloversatilis | 0.0004 |
| Order_Bacteroidales | 0.0004 |
| Order_Ktedonobacteria_C0119 | 0.0004 |
| Order_Rhodospirillales | 0.0004 |
| Patulibacter | 0.0004 |
| Phormidesmis_ANT_LACV5_1 | 0.0004 |
| Phycisphaeraceae_SM1A02 | 0.0004 |
| Porphyrobacter | 0.0004 |
| Propionimicrobium | 0.0004 |
| Proteiniclasticum | 0.0004 |
| Pseudarthrobacter | 0.0004 |
| Rahnella | 0.0004 |
| Reyranella | 0.0004 |
| Rufibacter | 0.0004 |
| Sediminivirga | 0.0004 |
| Singulisphaera | 0.0004 |
| Siphonobacter | 0.0004 |
| Sphingomonadaceae_DSSF69 | 0.0004 |
| Spiroplasma | 0.0004 |
| Tepidisphaera | 0.0004 |
| Terribacillus | 0.0004 |
| Thermoanaerobaculaceae_subgrp_10 | 0.0004 |
| Treponema | 0.0004 |
| _Eubacterium_yurii_group | 0.0003 |
| Acidibacter | 0.0003 |
| Amycolatopsis | 0.0003 |
| Anaerocolumna | 0.0003 |
| Ancylobacter | 0.0003 |
| Aquicella | 0.0003 |
| Barrientosiimonas | 0.0003 |
| Chishuiella | 0.0003 |
| Cupriavidus | 0.0003 |
| Dielma | 0.0003 |
| Eggerthella | 0.0003 |
| Epulopiscium | 0.0003 |
| Family_bacteriap25 | 0.0003 |
| Family_Bifidobacteriaceae | 0.0003 |
| Family_Clostridiales_Clostridiales_family_XI | 0.0003 |
| Family_Clostridiales_Clostridiales_family_XIII | 0.0003 |
| Family_Euzebyaceae | 0.0003 |
| Family_Myxococcales_Blfdi19 | 0.0003 |
| Family_Oligoflexales_0319_6G20 | 0.0003 |
| Family_Paenibacillaceae | 0.0003 |
| Family_Prevotellaceae | 0.0003 |
| Family_Saprospiraceae | 0.0003 |
| Family_Simkaniaceae | 0.0003 |
| Family_SR1_bacterium_canine_oral_taxon_380 | 0.0003 |
| Family_Steroidobacteraceae | 0.0003 |
| Flavitalea | 0.0003 |
| Galbitalea | 0.0003 |
| Gelidibacter | 0.0003 |
| Hahella | 0.0003 |
| Herbinix | 0.0003 |
| Kushneria | 0.0003 |
| Lachnospiraceae_FCS020 | 0.0003 |
| Lachnospiraceae_UCG_006 | 0.0003 |
| Lapillicoccus | 0.0003 |
| Larkinella | 0.0003 |
| Legionella | 0.0003 |
| Meiothermus | 0.0003 |
| Muricoccus | 0.0003 |
| Mycetocola | 0.0003 |
| Ohtaekwangia | 0.0003 |
| Order_Cytophagales | 0.0003 |
| Order_Pseudomonadales | 0.0003 |
| Ornithinicoccus | 0.0003 |
| Paenarthrobacter | 0.0003 |
| Parasutterella | 0.0003 |
| Phormidesmis_ANT_L52_6 | 0.0003 |
| Pigmentiphaga | 0.0003 |
| Pisciglobus | 0.0003 |
| Pusillimonas | 0.0003 |
| Raineyella | 0.0003 |
| Rhodobacter | 0.0003 |
| Ruminococcaceae_UCG_005 | 0.0003 |
| Sellimonas | 0.0003 |
| Streptomonospora | 0.0003 |
| Sutterella | 0.0003 |
| Tannerella | 0.0003 |
| Tepidiphilus | 0.0003 |
| Terrimicrobium | 0.0003 |
| Yersinia | 0.0003 |
| _Clostridium_innocuum_group | 0.0002 |
| Acetobacter | 0.0002 |
| Advenella | 0.0002 |
| Aequorivita | 0.0002 |
| Aeribacillus | 0.0002 |
| Alicyclobacillus | 0.0002 |
| Alistipes | 0.0002 |
| Anaerosporobacter | 0.0002 |
| Anaerovibrio | 0.0002 |
| Anaerovorax | 0.0002 |
| Anditalea | 0.0002 |
| Bacteriovorax | 0.0002 |
| Bordetella | 0.0002 |
| Brachymonas | 0.0002 |
| Camelimonas | 0.0002 |
| Candidatus_Hamiltonella | 0.0002 |
| Candidatus_Rhabdochlamydia | 0.0002 |
| Catenuloplanes | 0.0002 |
| Chroococcidiopsis_PCC_7203 | 0.0002 |
| Class_Actinobacteria_FFCH16263 | 0.0002 |
| Class_S0134_terrestrial_group | 0.0002 |
| Class_Thermoleophilia | 0.0002 |
| Clostridiales_Clostridiales_family_XIII_AD3011 | 0.0002 |
| Conchiformibius | 0.0002 |
| Confluentibacter | 0.0002 |
| Corticibacter | 0.0002 |
| Crossiella | 0.0002 |
| Desulfotomaculum | 0.0002 |
| Dyella | 0.0002 |
| Eggerthia | 0.0002 |
| Euzebyella | 0.0002 |
| Family_Alteromonadaceae | 0.0002 |
| Family_Bogoriellaceae | 0.0002 |
| Family_candidate_division_SR1_bacterium_taxon_345 | 0.0002 |
| Family_Cardiobacteriaceae | 0.0002 |
| Family_Dermabacteraceae | 0.0002 |
| Family_Erysipelotrichaceae | 0.0002 |
| Family_Eubacteriaceae | 0.0002 |
| Family_Flavobacteriaceae | 0.0002 |
| Family_Isosphaeraceae | 0.0002 |
| Family_Ktedonobacteraceae | 0.0002 |
| Family_Leptotrichiaceae | 0.0002 |
| Family_Methyloligellaceae | 0.0002 |
| Family_Methylophagaceae | 0.0002 |
| Family_Methylophilaceae | 0.0002 |
| Family_Micrococcales_AKAU3644 | 0.0002 |
| Family_Oligoflexaceae | 0.0002 |
| Family_Opitutaceae | 0.0002 |
| Family_Oxyphotobacteria_Incertae_Sedis_Unknown_Family | 0.0002 |
| Family_Peptococcaceae | 0.0002 |
| Family_Prolixibacteraceae | 0.0002 |
| Family_Rhodanobacteraceae | 0.0002 |
| Family_Verrucomicrobiaceae | 0.0002 |
| Family_Vibrionaceae | 0.0002 |
| Haoranjiania | 0.0002 |
| Hungatella | 0.0002 |
| Hyphomicrobium | 0.0002 |
| Jonesia | 0.0002 |
| Lachnospiraceae_UCG_003 | 0.0002 |
| Lachnospiraceae_UCG_004 | 0.0002 |
| Lacunisphaera | 0.0002 |
| Lewinella | 0.0002 |
| Longimicrobium | 0.0002 |
| Lutispora | 0.0002 |
| Mannheimia | 0.0002 |
| Marinomonas | 0.0002 |
| Mesorhizobium | 0.0002 |
| Microcoleus_PCC_7113 | 0.0002 |
| Mobilitalea | 0.0002 |
| Neochlamydia | 0.0002 |
| Niabella | 0.0002 |
| Nibribacter | 0.0002 |
| Nitrosomonas | 0.0002 |
| Nostoc_PCC_7107 | 0.0002 |
| Order_Anaerolineae_RBG_13_54_9 | 0.0002 |
| Order_Coriobacteriales | 0.0002 |
| Order_Elsterales | 0.0002 |
| Order_Gammaproteobacteria_R7C24 | 0.0002 |
| Order_Solirubrobacterales | 0.0002 |
| Oxyphotobacteria_Incertae_Sedis_Unknown_Family_EcFYyy_200 | 0.0002 |
| Parabacteroides | 0.0002 |
| Parvibaculum | 0.0002 |
| Pelosinus | 0.0002 |
| Persicitalea | 0.0002 |
| Phormidium_SAG_37_90 | 0.0002 |
| Phyllobacterium | 0.0002 |
| Planktothricoides_SR001 | 0.0002 |
| Plantibacter | 0.0002 |
| Procabacter | 0.0002 |
| Pseudorhodobacter | 0.0002 |
| Pseudorhodoplanes | 0.0002 |
| Ramlibacter | 0.0002 |
| Rhodomicrobium | 0.0002 |
| Rhodovastum | 0.0002 |
| Roseateles | 0.0002 |
| Roseiflexus | 0.0002 |
| Salinicola | 0.0002 |
| Salipaludibacillus | 0.0002 |
| Sandaracinobacter | 0.0002 |
| Sedimentibacter | 0.0002 |
| Sediminibacterium | 0.0002 |
| Senegalimassilia | 0.0002 |
| Shimazuella | 0.0002 |
| Simplicispira | 0.0002 |
| Solirubrobacteraceae_JCM_18997 | 0.0002 |
| Sphingomonadaceae_Ellin6055 | 0.0002 |
| Stenotrophobacter | 0.0002 |
| Steroidobacter | 0.0002 |
| Streptosporangium | 0.0002 |
| Subsaxibacter | 0.0002 |
| Tardiphaga | 0.0002 |
| Tepidimicrobium | 0.0002 |
| Tetrasphaera | 0.0002 |
| Thermus | 0.0002 |
| TM7_phylum_sp_canine_oral_taxon_322 | 0.0002 |
| Trueperella | 0.0002 |
| Ureibacillus | 0.0002 |
| Zoogloea | 0.0002 |
| _Anaerorhabdus_furcosa_group | 0.0001 |
| _Eubacterium_ruminantium_group | 0.0001 |
| Acetitomaculum | 0.0001 |
| Acetivibrio | 0.0001 |
| Acetobacterium | 0.0001 |
| Acidipropionibacterium | 0.0001 |
| Acidisphaera | 0.0001 |
| Acidithiobacillaceae_KCM_B_112 | 0.0001 |
| Actinocorallia | 0.0001 |
| Agromyces | 0.0001 |
| Alcanivorax | 0.0001 |
| Aliidiomarina | 0.0001 |
| Alkaliphilus | 0.0001 |
| Allofustis | 0.0001 |
| Alteromonas | 0.0001 |
| Aminobacter | 0.0001 |
| Anaerobacillus | 0.0001 |
| Anaerobacterium | 0.0001 |
| Anaerobiospirillum | 0.0001 |
| Anaeromyxobacter | 0.0001 |
| Anaerosinus | 0.0001 |
| Ancylothrix_8PC | 0.0001 |
| Apibacter | 0.0001 |
| Aquamicrobium | 0.0001 |
| Aquisphaera | 0.0001 |
| Arachidicoccus | 0.0001 |
| Arenimonas | 0.0001 |
| Atopobiaceae_UCG_002 | 0.0001 |
| Barnesiella | 0.0001 |
| Bauldia | 0.0001 |
| Bogoriella | 0.0001 |
| Bradyrhizobium | 0.0001 |
| Caldalkalibacillus | 0.0001 |
| Candidatus_Amoebophilus | 0.0001 |
| Candidatus_Ancillula | 0.0001 |
| Candidatus_Endoecteinascidia | 0.0001 |
| Candidatus_Entotheonella | 0.0001 |
| Candidatus_Finniella | 0.0001 |
| Candidatus_Megaira | 0.0001 |
| Candidatus_Microthrix | 0.0001 |
| Candidatus_Nostocoida | 0.0001 |
| Candidatus_Odyssella | 0.0001 |
| Candidatus_Saccharibacteria_bacterium_RAAC3_TM7_1 | 0.0001 |
| Candidimonas | 0.0001 |
| Caproiciproducens | 0.0001 |
| Cavicella | 0.0001 |
| Cedecea | 0.0001 |
| Celeribacter | 0.0001 |
| Centipeda | 0.0001 |
| Cesiribacter | 0.0001 |
| Cetobacterium | 0.0001 |
| Chelativorans | 0.0001 |
| Chelatococcus | 0.0001 |
| Chondromyces | 0.0001 |
| Chromohalobacter | 0.0001 |
| Chungangia | 0.0001 |
| Citricoccus | 0.0001 |
| Class_Acidobacteria_subgrp_17 | 0.0001 |
| Class_Chloroflexi_JG30_KF_CM66 | 0.0001 |
| Class_Deltaproteobacteria | 0.0001 |
| Class_Elusimicrobia_Lineage_IIb | 0.0001 |
| Class_Parcubacteria | 0.0001 |
| Class_Sericytochromatia | 0.0001 |
| Coleofasciculus_CCY0602 | 0.0001 |
| Commensalibacter | 0.0001 |
| Crinalium_SAG_22_89 | 0.0001 |
| Cryptobacterium | 0.0001 |
| Cryptosporangium | 0.0001 |
| Cuspidothrix_LMECYA_163 | 0.0001 |
| Cystobacter | 0.0001 |
| Dechloromonas | 0.0001 |
| Defluviitaleaceae_UCG_011 | 0.0001 |
| Desulfitibacter | 0.0001 |
| Desulfovibrio | 0.0001 |
| Desulfurispora | 0.0001 |
| Dinghuibacter | 0.0001 |
| Diplorickettsia | 0.0001 |
| Dolosicoccus | 0.0001 |
| Eisenbergiella | 0.0001 |
| Ensifer | 0.0001 |
| Enterorhabdus | 0.0001 |
| Erysipelotrichaceae_UCG_004 | 0.0001 |
| Erysipelotrichaceae_ZOR0006 | 0.0001 |
| Faecalibaculum | 0.0001 |
| Faecalicoccus | 0.0001 |
| Faecalitalea | 0.0001 |
| Family_Amb_16S_1323 | 0.0001 |
| Family_Atopobiaceae | 0.0001 |
| Family_Azospirillaceae | 0.0001 |
| Family_Betaproteobacteriales_SC_I_84 | 0.0001 |
| Family_Bradymonadaceae | 0.0001 |
| Family_Brevibacteriaceae | 0.0001 |
| Family_Candidatus_Azambacteria_bacterium_GW2011_GWC2_45_7b | 0.0001 |
| Family_Cellulomonadaceae | 0.0001 |
| Family_Chlamydiales_cvE6 | 0.0001 |
| Family_Chthoniobacteraceae | 0.0001 |
| Family_Clostridiales_vadinBB60_group | 0.0001 |
| Family_Coleofasciculaceae | 0.0001 |
| Family_Eggerthellaceae | 0.0001 |
| Family_Entotheonellaceae | 0.0001 |
| Family_Fibrobacteraceae | 0.0001 |
| Family_Fibrobacterales_possible_family_01 | 0.0001 |
| Family_Halieaceae | 0.0001 |
| Family_Hydrogenophilaceae | 0.0001 |
| Family_Hymenobacteraceae | 0.0001 |
| Family_Lactobacillaceae | 0.0001 |
| Family_Leptolyngbyaceae | 0.0001 |
| Family_Marinifilaceae | 0.0001 |
| Family_Methylacidiphilaceae | 0.0001 |
| Family_Methylopilaceae | 0.0001 |
| Family_Micavibrionaceae | 0.0001 |
| Family_Microtrichaceae | 0.0001 |
| Family_Midichloriaceae | 0.0001 |
| Family_Mycoplasmataceae | 0.0001 |
| Family_Parachlamydiaceae | 0.0001 |
| Family_Pedosphaeraceae | 0.0001 |
| Family_Pirellulaceae | 0.0001 |
| Family_Rhizobiales_Incertae_Sedis | 0.0001 |
| Family_Rhizobiales_KF_JG30_B3 | 0.0001 |
| Family_Rhodocyclaceae | 0.0001 |
| Family_Rickettsiales_SM2D12 | 0.0001 |
| Family_Saccharum_hybrid_cultivar | 0.0001 |
| Family_Solibacteraceae_subgrp_3 | 0.0001 |
| Family_Syntrophomonadaceae | 0.0001 |
| Family_Tannerellaceae | 0.0001 |
| Family_Vermiphilaceae | 0.0001 |
| Family_Verrucomicrobiales_DEV007 | 0.0001 |
| Faucicola | 0.0001 |
| Flaviaesturariibacter | 0.0001 |
| Flavimarina | 0.0001 |
| Flavonifractor | 0.0001 |
| Flexibacter | 0.0001 |
| Fusibacter | 0.0001 |
| Galbibacter | 0.0001 |
| Garciella | 0.0001 |
| Gleocapsa | 0.0001 |
| Gracilibacter | 0.0001 |
| Gramella | 0.0001 |
| Gryllotalpicola | 0.0001 |
| Gulbenkiania | 0.0001 |
| Halieaceae_OM60_NOR5 | 0.0001 |
| Halobacillus | 0.0001 |
| Halocella | 0.0001 |
| Haloplasma | 0.0001 |
| Hansschlegelia | 0.0001 |
| Hirschia | 0.0001 |
| Hydrocarboniphaga | 0.0001 |
| Hydrogenophaga | 0.0001 |
| Idiomarina | 0.0001 |
| Inquilinus | 0.0001 |
| Intestinimonas | 0.0001 |
| Jahnella | 0.0001 |
| Komagataeibacter | 0.0001 |
| Krasilnikovia | 0.0001 |
| Kribbella | 0.0001 |
| Ktedonobacter | 0.0001 |
| Labrys | 0.0001 |
| Lachnospiraceae_AC2044 | 0.0001 |
| Lachnospiraceae_ASF356 | 0.0001 |
| Lachnospiraceae_NC2004 | 0.0001 |
| Lachnotalea | 0.0001 |
| LB3_76 | 0.0001 |
| Lechevalieria | 0.0001 |
| Leptolinea | 0.0001 |
| Leptolyngbya_ANT_L67_1 | 0.0001 |
| Leptolyngbyaceae_MIZ36 | 0.0001 |
| Lihuaxuella | 0.0001 |
| Limnochorda | 0.0001 |
| Listeria | 0.0001 |
| Litorilinea | 0.0001 |
| Luedemannella | 0.0001 |
| Lunatimonas | 0.0001 |
| Maribacter | 0.0001 |
| Marinactinospora | 0.0001 |
| Marinilutecoccus | 0.0001 |
| Marvinbryantia | 0.0001 |
| Mastigocladopsis_PCC_10914 | 0.0001 |
| Methylopila | 0.0001 |
| Methylorosula | 0.0001 |
| Micavibrio | 0.0001 |
| Microtrichaceae_Sva0996_marine_group | 0.0001 |
| Millisia | 0.0001 |
| Moritella | 0.0001 |
| Myroides | 0.0001 |
| Myxococcales_bacterium_Gsoil_473 | 0.0001 |
| Neorickettsia | 0.0001 |
| Nitratireductor | 0.0001 |
| Nitrolancea | 0.0001 |
| Nitrosomonadaceae_MND1 | 0.0001 |
| Niveispirillum | 0.0001 |
| Nocardia | 0.0001 |
| Nonomuraea | 0.0001 |
| Nostoc_PCC_7524 | 0.0001 |
| Nostoc_SAG_60_79 | 0.0001 |
| Novibacillus | 0.0001 |
| Olleya | 0.0001 |
| Opitutus | 0.0001 |
| Order_Anaerolineae_SJA_15 | 0.0001 |
| Order_bacterium_Ellin6529 | 0.0001 |
| Order_Blastocatellia_subgrp_4_DS_100 | 0.0001 |
| Order_Candidatus_Kaiserbacteria | 0.0001 |
| Order_Chlamydiales | 0.0001 |
| Order_Dehalococcoidia_S085 | 0.0001 |
| Order_Deltaproteobacteria_NB1_j | 0.0001 |
| Order_Deltaproteobacteria_SAR324_clade_Marine_group_B_ | 0.0001 |
| Order_Gammaproteobacteria_B2M28 | 0.0001 |
| Order_Gammaproteobacteria_CCD24 | 0.0001 |
| Order_Hyaloperonospora_arabidopsidis | 0.0001 |
| Order_Ignavibacteria_SJA_28 | 0.0001 |
| Order_Kiritimatiellae_WCHB1_41 | 0.0001 |
| Order_Limnochordales | 0.0001 |
| Order_Thalassobaculales | 0.0001 |
| Order_Xanthomonadales | 0.0001 |
| Ornithobacterium | 0.0001 |
| Oscillatoria_SAG_1459_8 | 0.0001 |
| Oscillibacter | 0.0001 |
| Paludisphaera | 0.0001 |
| Pannonibacter | 0.0001 |
| Paraclostridium | 0.0001 |
| Parafilimonas | 0.0001 |
| Parasegetibacter | 0.0001 |
| Pediococcus | 0.0001 |
| Pedomicrobium | 0.0001 |
| Persicirhabdus | 0.0001 |
| Phreatobacter | 0.0001 |
| Phycicoccus | 0.0001 |
| Phylum_Armatimonadetes | 0.0001 |
| Pirellulaceae_Pir4 | 0.0001 |
| Planctomicrobium | 0.0001 |
| Propionibacterium | 0.0001 |
| Propionicimonas | 0.0001 |
| Proteiniborus | 0.0001 |
| Providencia | 0.0001 |
| Pseudoflavitalea | 0.0001 |
| Pseudogracilibacillus | 0.0001 |
| Rhodopila | 0.0001 |
| Rikenellaceae_RC9 | 0.0001 |
| Roseivirga | 0.0001 |
| Roseovarius | 0.0001 |
| Rudanella | 0.0001 |
| Ruminococcaceae_CAG_352 | 0.0001 |
| Ruminococcaceae_UCG_004 | 0.0001 |
| Ruminococcaceae_UCG_010 | 0.0001 |
| Salegentibacter | 0.0001 |
| Schlegelella | 0.0001 |
| Serinicoccus | 0.0001 |
| Siccibacter | 0.0001 |
| Silvanigrella | 0.0001 |
| Snodgrassella | 0.0001 |
| Soonwooa | 0.0001 |
| Spelaeicoccus | 0.0001 |
| Spongiibacteraceae_BD1_7 | 0.0001 |
| Sporobacter | 0.0001 |
| Sporolactobacillus | 0.0001 |
| Sporomusa | 0.0001 |
| Stappia | 0.0001 |
| Stigmatella | 0.0001 |
| Stigonema_SAG_48_90 | 0.0001 |
| Succiniclasticum | 0.0001 |
| Symbiobacterium | 0.0001 |
| Symplocastrum_CPER_KK1 | 0.0001 |
| Syntrophaceticus | 0.0001 |
| Tepidimonas | 0.0001 |
| Tetragenococcus | 0.0001 |
| Thermacetogenium | 0.0001 |
| Thermaerobacter | 0.0001 |
| Thermobifida | 0.0001 |
| Thermobispora | 0.0001 |
| Thermoflavimicrobium | 0.0001 |
| TM7_phylum_sp_canine_oral_taxon_308 | 0.0001 |
| Tomitella | 0.0001 |
| Tuberibacillus | 0.0001 |
| Tunicatimonas | 0.0001 |
| Ulvibacter | 0.0001 |
| unc_Caldilinea | 0.0001 |
| Ureaplasma | 0.0001 |
| Variibacter | 0.0001 |
| Virgisporangium | 0.0001 |
| Viridibacillus | 0.0001 |
| Vogesella | 0.0001 |
| Wolinella | 0.0001 |
| Xanthobacter | 0.0001 |
| Yaniella | 0.0001 |

**Table S4. Fungal abundance in bed dust samples at phylum level.**

| **Phylum** | **Abundance (%)** |
| --- | --- |
| Ascomycota | 82.4709 |
| Kingdom_Fungi | 10.5311 |
| Basidiomycota | 6.5984 |
| Mucoromycota | 0.3209 |
| Chytridiomycota | 0.0693 |
| Mortierellomycota | 0.0094 |
| Entomophthoromycota | 0.0001 |

**Table S5. Fungal abundance in bed dust samples at class level.**

| **Class** |  | **Abundance (%)** |
| --- | --- | --- |
| Phylum_Ascomycota |  | 43.9821 |
| Dothideomycetes |  | 27.5675 |
| Kingdom_Fungi |  | 10.5311 |
| Saccharomycetes |  | 5.25 |
| Eurotiomycetes |  | 3.552 |
| Phylum_Basidiomycota |  | 3.2063 |
| Agaricomycetes |  | 2.0208 |
| Lecanoromycetes |  | 1.247 |
| Malasseziomycetes |  | 0.6761 |
| Sordariomycetes |  | 0.5327 |
| Leotiomycetes |  | 0.3249 |
| Mucoromycetes |  | 0.3209 |
| Microbotryomycetes |  | 0.2602 |
| Cystobasidiomycetes |  | 0.2294 |
| Pucciniomycetes |  | 0.1464 |
| Phylum_Chytridiomycota |  | 0.0613 |
| Ustilaginomycetes |  | 0.0471 |
| Pezizomycetes |  | 0.014 |
| Exobasidiomycetes |  | 0.0115 |
| Mortierellomycetes |  | 0.0094 |
| Rhizophlyctidomycetes |  | 0.0056 |
| Spizellomycetes |  | 0.0024 |
| Orbiliomycetes |  | 0.0008 |
| Tremellomycetes |  | 0.0006 |
| Basidiobolomycetes |  | 0.0001 |

**Table S6.** Fungal abundance in bed dust samples at genera level.

| **Genus** | **Abundance (%)** |
| --- | --- |
| Phylum_Ascomycota | 43.9821 |
| Kingdom_Fungi | 10.5311 |
| Spegazzinia | 9.6071 |
| Aureobasidium | 5.3379 |
| Sphaerellopsis | 4.9933 |
| Curvularia | 4.8287 |
| Saccharomyces | 4.5007 |
| Penicillium | 3.552 |
| Phylum_Basidiomycota | 3.2063 |
| Family_Pleosporaceae | 1.6057 |
| Class_Agaricomycetes | 1.2952 |
| Class_Lecanoromycetes | 0.8065 |
| Wickerhamomyces | 0.6966 |
| Malassezia | 0.6761 |
| Neophaeosphaeria | 0.6594 |
| Order_Agaricales | 0.6444 |
| Order_Pleosporales | 0.332 |
| Mucor | 0.3209 |
| Class_Sordariomycetes | 0.306 |
| Coleophoma | 0.2912 |
| Family_Sporidiobolaceae | 0.2416 |
| Erythrobasidium | 0.2294 |
| Physcia | 0.1418 |
| Family_Leptosphaeriaceae | 0.1304 |
| Family_Physciaceae | 0.1006 |
| Order_Lecanorales | 0.0793 |
| Family_Parmeliaceae | 0.0749 |
| Order_Sordariales | 0.0743 |
| Phylum_Chytridiomycota | 0.0613 |
| Melampsora | 0.0555 |
| Uromyces | 0.0496 |
| Family_Ustilaginaceae | 0.0468 |
| Order_Xylariales | 0.0436 |
| Preussia | 0.0381 |
| Family_Teloschistaceae | 0.0373 |
| Order_Pucciniales | 0.0361 |
| Termitomyces | 0.0264 |
| Oidiodendron | 0.0253 |
| Kazachstania | 0.0244 |
| Cytospora | 0.0237 |
| Trichoderma | 0.0225 |
| Arthrinium | 0.02 |
| Borofutus | 0.017 |
| Order_Saccharomycetales | 0.0159 |
| Arxiella | 0.0152 |
| Class_Microbotryomycetes | 0.0136 |
| Order_Auriculariales | 0.0111 |
| Class_Dothideomycetes | 0.0101 |
| Mortierella | 0.0094 |
| Family_Xylariaceae | 0.0091 |
| Lodderomyces | 0.0088 |
| Ruhlandiella | 0.0084 |
| Order_Capnodiales | 0.0071 |
| Order_Helotiales | 0.0071 |
| Pseudomicrostroma | 0.0071 |
| Pilatoporus | 0.0063 |
| Borealophlyctis | 0.0056 |
| Oleoguttula | 0.0053 |
| Trichophaea | 0.0052 |
| Sporobolomyces | 0.005 |
| Bambusaria | 0.0047 |
| Family_Ceratobasidiaceae | 0.0043 |
| Family_Magnaporthaceae | 0.004 |
| Thanatephorus | 0.0037 |
| Melampsoridium | 0.0034 |
| Candida | 0.0029 |
| Metarhizium | 0.0029 |
| Aspicilia | 0.0027 |
| Hyphodontia | 0.0027 |
| Order_Microstromatales | 0.0027 |
| Phlogicylindrium | 0.0027 |
| Order_Diaporthales | 0.0025 |
| Spizellomyces | 0.0021 |
| Family_Mycosphaerellaceae | 0.002 |
| Monilochaetes | 0.002 |
| Artomyces | 0.0019 |
| Exobasidium | 0.0017 |
| Eutypella | 0.0016 |
| Massaria | 0.0014 |
| Clavulina | 0.0012 |
| Family_Pucciniastraceae | 0.0011 |
| Family_Sclerotiniaceae | 0.0011 |
| Pleurotus | 0.0011 |
| Sarea | 0.0011 |
| Staninwardia | 0.0011 |
| Cyathus | 0.001 |
| Corynespora | 0.0009 |
| Arthrobotrys | 0.0008 |
| Polycauliona | 0.0008 |
| Xylaria | 0.0008 |
| Caloplaca | 0.0007 |
| Gyrodon | 0.0007 |
| Stenella | 0.0007 |
| Daldinia | 0.0006 |
| Family_Thelephoraceae | 0.0006 |
| Heterocephalacria | 0.0006 |
| Hymenopellis | 0.0006 |
| Setosphaeria | 0.0006 |
| Cortinarius | 0.0005 |
| Entoloma | 0.0005 |
| Family_Helicobasidiaceae | 0.0005 |
| Family_Ramalinaceae | 0.0005 |
| Family_Saccharomycetaceae | 0.0005 |
| Pseudoramichloridium | 0.0005 |
| Melanogaster | 0.0003 |
| Peziza | 0.0003 |
| Phellinus | 0.0003 |
| Poaceascoma | 0.0003 |
| Bussabanomyces | 0.0002 |
| Class_Leotiomycetes | 0.0002 |
| Dirkmeia | 0.0002 |
| Family_Spizellomycetaceae | 0.0002 |
| Family_Xylariales_fam_Incertae_sedis | 0.0002 |
| Fulgidea | 0.0002 |
| Hypoxylon | 0.0002 |
| Lentinus | 0.0002 |
| Order_Hypocreales | 0.0002 |
| Order_Russulales | 0.0002 |
| Thekopsora | 0.0002 |
| Tylospora | 0.0002 |
| Annulohypoxylon | 0.0001 |
| Anungitea | 0.0001 |
| Basidiobolus | 0.0001 |
| Derxomyces | 0.0001 |
| Family_Cortinariaceae | 0.0001 |
| Family_Morchellaceae | 0.0001 |
| Family_Umbilicariaceae | 0.0001 |
| Hansfordia | 0.0001 |
| Hypogymnia | 0.0001 |
| Inocybe | 0.0001 |
| Kochiomyces | 0.0001 |
| Neofabraea | 0.0001 |
| Order_Boletales | 0.0001 |
| Parmelia | 0.0001 |
| Pyricularia | 0.0001 |
| Ramalina | 0.0001 |
| Ramaria | 0.0001 |
| Amphinema | 0 |
| Bryoria | 0 |
| Cetrelia | 0 |
| Chroogomphus | 0 |
| Cronartium | 0 |
| Family_Acarosporaceae | 0 |
| Family_Atheliaceae | 0 |
| Family_Megasporaceae | 0 |
| Imleria | 0 |
| Lactarius | 0 |
| Lophodermium | 0 |
| Pogonoloma | 0 |
| Toninia | 0 |
| Umbilicaria | 0 |

**Table S7.** Differentially abundant bacterial taxa in bed dust samples for homes with dog and cat both. †Column labeled “Pet ownership” represents the home that have dog and cat both in which the corresponding taxa (as presented in column labeled “Differentially abundant Taxa”), was found to be significantly differentially abundant by LEfSe. ‡ Differentially abundant taxa is described using the following hierarchy: Phylum|Class|Order|Family|Genus|species.

| **Taxa‡** | **Pet ownership†** | **LDA_Value** | **pvalue** |
| --- | --- | --- | --- |
| Firmicutes|Clostridia | Cat and Dog both | 4.688521671 | 0.000319586 |
| Firmicutes|Clostridia|Clostridiales | Cat and Dog both | 4.688521671 | 0.000319586 |
| Bacteroidetes|Bacteroidia | Cat and Dog both | 4.195648296 | 0.02561768 |
| Bacteroidetes | Cat and Dog both | 4.193632123 | 0.026638765 |
| Firmicutes|Clostridia|Clostridiales|Lachnospiraceae|Lachnoanaerobaculum|Lachnoanaerobaculum|Genus_Lachnoanaerobaculum | Cat and Dog both | 4.128818879 | 0.024368767 |
| Firmicutes|Clostridia|Clostridiales|Lachnospiraceae|Lachnoanaerobaculum|Lachnoanaerobaculum | Cat and Dog both | 4.121663312 | 0.024368767 |
| Firmicutes|Clostridia|Clostridiales|Lachnospiraceae|Lachnoanaerobaculum | Cat and Dog both | 4.121361381 | 0.024368767 |
| Bacteroidetes|Bacteroidia|Sphingobacteriales | Cat and Dog both | 4.057171605 | 0.004526178 |
| Bacteroidetes|Bacteroidia|Sphingobacteriales|Sphingobacteriaceae | Cat and Dog both | 4.051922334 | 0.008884013 |
| Firmicutes|Clostridia|Clostridiales|Peptostreptococcaceae|Terrisporobacter|Terrisporobacter|Genus_Terrisporobacter | Cat and Dog both | 4.043176728 | 0.02319299 |
| Firmicutes|Clostridia|Clostridiales|Lachnospiraceae | Cat and Dog both | 4.02008382 | 0.014092826 |
| Firmicutes|Clostridia|Clostridiales|Peptostreptococcaceae|Terrisporobacter | Cat and Dog both | 4.019070933 | 0.02319299 |
| Bacteroidetes|Bacteroidia|Sphingobacteriales|Sphingobacteriaceae|Pedobacter|Pedobacter | Cat and Dog both | 4.013850526 | 0.015068055 |
| Firmicutes|Clostridia|Clostridiales|Peptostreptococcaceae|Terrisporobacter|Terrisporobacter | Cat and Dog both | 4.002454452 | 0.02319299 |
| Bacteroidetes|Bacteroidia|Flavobacteriales|Weeksellaceae|Bergeyella | Cat and Dog both | 3.998829903 | 0.016974146 |
| Bacteroidetes|Bacteroidia|Sphingobacteriales|Sphingobacteriaceae|Pedobacter | Cat and Dog both | 3.993856714 | 0.015068055 |
| Bacteroidetes|Bacteroidia|Flavobacteriales|Weeksellaceae|Bergeyella|Bergeyella | Cat and Dog both | 3.983208488 | 0.016974146 |
| Bacteroidetes|Bacteroidia|Sphingobacteriales|Sphingobacteriaceae|Pedobacter|Pedobacter|Genus_Pedobacter | Cat and Dog both | 3.958628636 | 0.004855026 |
| Actinobacteria|Acidimicrobiia | Cat and Dog both | 3.949538033 | 0.00000163 |
| Actinobacteria|Acidimicrobiia|Microtrichales | Cat and Dog both | 3.949085911 | 0.000000748 |
| Firmicutes|Erysipelotrichia|Erysipelotrichales|Erysipelotrichaceae|Turicibacter | Cat and Dog both | 3.943624883 | 0.000195682 |
| Firmicutes|Erysipelotrichia|Erysipelotrichales|Erysipelotrichaceae|Turicibacter | Cat and Dog both | 3.935006171 | 0.000195682 |
| Firmicutes|Erysipelotrichia|Erysipelotrichales|Erysipelotrichaceae|Turicibacter | Cat and Dog both | 3.933028674 | 0.000195682 |
| Firmicutes|Erysipelotrichia|Erysipelotrichales | Cat and Dog both | 3.923147654 | 0.00145805 |
| Firmicutes|Erysipelotrichia|Erysipelotrichales|Erysipelotrichaceae | Cat and Dog both | 3.922210763 | 0.00145805 |
| Firmicutes|Erysipelotrichia | Cat and Dog both | 3.916021631 | 0.00145805 |
| Bacteroidetes|Bacteroidia|Flavobacteriales|Weeksellaceae | Cat and Dog both | 3.911975512 | 0.034286454 |
| Firmicutes|Bacilli|Bacillales|Staphylococcaceae|Staphylococcus|Staphylococcus|Staphylococcus_equorum | Cat and Dog both | 3.903551678 | 0.042350514 |
| Actinobacteria|Acidimicrobiia|Microtrichales|Ilumatobacteraceae | Cat and Dog both | 3.887377448 | 0.000000416 |
| Actinobacteria|Actinobacteria|Corynebacteriales|Corynebacteriaceae|Corynebacterium|Corynebacterium_amycolatum | Cat and Dog both | 3.875325029 | 0.000489236 |
| Actinobacteria|Acidimicrobiia|Microtrichales|Ilumatobacteraceae|Ilumatobacter | Cat and Dog both | 3.836410787 | 0.00000358 |
| Actinobacteria|Acidimicrobiia|Microtrichales|Ilumatobacteraceae|Ilumatobacter | Cat and Dog both | 3.834725427 | 0.00000358 |
| Actinobacteria|Acidimicrobiia|Microtrichales|Ilumatobacteraceae|Ilumatobacter | Cat and Dog both | 3.832501173 | 0.00000358 |
| Cyanobacteria|Oxyphotobacteria|Nostocales | Cat and Dog both | 3.820664436 | 0.008219886 |
| Cyanobacteria|Oxyphotobacteria|Nostocales|Phormidiaceae | Cat and Dog both | 3.798728957 | 0.005120068 |
| Cyanobacteria|Oxyphotobacteria|Nostocales|Phormidiaceae|Tychonema_CCAP_1459_11B | Cat and Dog both | 3.791198527 | 0.005120068 |
| Cyanobacteria|Oxyphotobacteria|Nostocales|Phormidiaceae|Tychonema_CCAP_1459_11B|Tychonema_CCAP_1459_11B | Cat and Dog both | 3.790972613 | 0.005120068 |
| Firmicutes|Clostridia|Clostridiales|Clostridiaceae | Cat and Dog both | 3.781664559 | 0.000000119 |
| Cyanobacteria|Oxyphotobacteria|Nostocales|Phormidiaceae|Tychonema_CCAP_1459_11B|Tychonema_CCAP_1459_11B|Genus_Tychonema_CCAP_1459_11B | Cat and Dog both | 3.781066143 | 0.005120068 |
| Firmicutes|Bacilli|Bacillales|Bacillaceae | Cat and Dog both | 3.778449371 | 0.001236611 |
| Firmicutes|Bacilli|Bacillales|Bacillaceae|Sinobaca|Sinobaca | Cat and Dog both | 3.638365115 | 0.001757456 |
| Firmicutes|Bacilli|Bacillales|Bacillaceae|Sinobaca | Cat and Dog both | 3.632590135 | 0.001757456 |
| Firmicutes|Clostridia|Clostridiales|Clostridiaceae|Clostridium_sensu_stricto | Cat and Dog both | 3.630739171 | 0.0000716 |
| Firmicutes|Clostridia|Clostridiales|Clostridiaceae|Clostridium_sensu_stricto | Cat and Dog both | 3.61804905 | 0.0000716 |
| Firmicutes|Bacilli|Bacillales|Planococcaceae | Cat and Dog both | 3.611780605 | 0.004152413 |
| Firmicutes|Bacilli|Bacillales|Bacillaceae|Sinobaca|Sinobaca|Genus_Sinobaca | Cat and Dog both | 3.604828555 | 0.001757456 |
| Firmicutes|Clostridia|Clostridiales|Ruminococcaceae|Fastidiosipila|Fastidiosipila | Cat and Dog both | 3.577296566 | 0.020477963 |
| Firmicutes|Clostridia|Clostridiales|Ruminococcaceae|Fastidiosipila | Cat and Dog both | 3.548563618 | 0.020477963 |
| Firmicutes|Clostridia|Clostridiales|Clostridiaceae|Clostridium_sensu_stricto | Cat and Dog both | 3.531901276 | 0.000000000153 |
| Firmicutes|Clostridia|Clostridiales|Clostridiaceae|Clostridium_sensu_stricto | Cat and Dog both | 3.526079421 | 0.000000000153 |
| Firmicutes|Clostridia|Clostridiales|Clostridiaceae|Clostridium_sensu_stricto | Cat and Dog both | 3.523396483 | 0.000000000153 |
| Firmicutes|Clostridia|Clostridiales|Clostridiaceae|Clostridium_sensu_stricto|Clostridium_perfringens | Cat and Dog both | 3.50831152 | 0.0000639 |
| Cyanobacteria|Oxyphotobacteria | No pets | 4.727615132 | 0.018215131 |
| Actinobacteria|Actinobacteria | No pets | 4.60755514 | 0.040459178 |
| Actinobacteria|Actinobacteria|Micrococcales | No pets | 4.578229125 | 0.01588018 |
| Actinobacteria|Actinobacteria|Micrococcales|Micrococcaceae | No pets | 4.495930572 | 0.015152492 |
| Actinobacteria|Actinobacteria|Micrococcales|Micrococcaceae|Kocuria | No pets | 4.409819534 | 0.017566053 |
| Actinobacteria|Actinobacteria|Micrococcales|Micrococcaceae|Kocuria|Kocuria | No pets | 4.389223698 | 0.017566053 |
| Firmicutes|Bacilli|Lactobacillales | No pets | 4.380454633 | 0.031985375 |
| Proteobacteria|Gammaproteobacteria|Pseudomonadales|Pseudomonadaceae | No pets | 4.245375285 | 0.037861526 |
| Proteobacteria|Gammaproteobacteria|Pseudomonadales|Pseudomonadaceae|Pseudomonas|Pseudomonas | No pets | 4.231147927 | 0.026901727 |
| Proteobacteria|Gammaproteobacteria|Pseudomonadales|Pseudomonadaceae|Pseudomonas | No pets | 4.222336848 | 0.026901727 |
| Firmicutes|Bacilli|Lactobacillales|Streptococcaceae | No pets | 3.798764292 | 0.020344579 |
| Proteobacteria|Gammaproteobacteria|Pseudomonadales|Moraxellaceae|Acinetobacter | No pets | 3.769166672 | 0.041874852 |
| Proteobacteria|Gammaproteobacteria|Pseudomonadales|Moraxellaceae|Acinetobacter|Acinetobacter | No pets | 3.767130008 | 0.041874852 |
| Fusobacteria|Fusobacteriia|Fusobacteriales|Leptotrichiaceae|Streptobacillus | No pets | 3.67697825 | 0.033347899 |
| Fusobacteria|Fusobacteriia|Fusobacteriales|Leptotrichiaceae|Streptobacillus|Streptobacillus | No pets | 3.669463102 | 0.033347899 |
| Fusobacteria|Fusobacteriia|Fusobacteriales|Leptotrichiaceae|Streptobacillus|Streptobacillus|Genus_Streptobacillus | No pets | 3.668154707 | 0.033347899 |

**Table S8.** Differentially abundant bacterial taxa in bed dust samples for homes with dog. †Column labeled “Pet ownership” represents the home that have dog in which the corresponding taxa (as presented in column labeled “Differentially abundant Taxa”), was found to be significantly differentially abundant by LEfSe. ‡ Differentially abundant taxa is described using the following hierarchy: Phylum|Class|Order|Family|Genus|species.

| **Taxa‡** | **Pet ownership†** | **LDA_Value** | **pvalue** |
| --- | --- | --- | --- |
| Actinobacteria|Acidimicrobiia | Dog only | 3.594289995 | 0.009696226 |
| Actinobacteria|Acidimicrobiia|Microtrichales | Dog only | 3.59888375 | 0.005958639 |
| Firmicutes|Clostridia|Clostridiales|Peptostreptococcaceae|Paeniclostridium | Dog only | 3.558061288 | 0.000567912 |
| Firmicutes|Clostridia|Clostridiales|Peptostreptococcaceae|Paeniclostridium | Dog only | 3.549902091 | 0.000567912 |
| Firmicutes|Clostridia|Clostridiales|Peptostreptococcaceae|Paeniclostridium | Dog only | 3.54653297 | 0.000567912 |
| Actinobacteria|Coriobacteriia | Dog only | 3.870532463 | 0.01114614 |
| Actinobacteria|Coriobacteriia|Coriobacteriales | Dog only | 3.91341703 | 0.01114614 |
| Actinobacteria|Coriobacteriia|Coriobacteriales|Atopobiaceae | Dog only | 3.663329576 | 0.009497387 |
| Actinobacteria|Coriobacteriia|Coriobacteriales|Atopobiaceae|Atopobium | Dog only | 3.655902395 | 0.007920433 |
| Actinobacteria|Coriobacteriia|Coriobacteriales|Atopobiaceae|Atopobium | Dog only | 3.66222444 | 0.007920433 |
| Actinobacteria|Coriobacteriia|Coriobacteriales|Atopobiaceae|Atopobium|Atopobium_vaginae | Dog only | 3.59776641 | 0.013984842 |
| Cyanobacteria|Oxyphotobacteria|Nostocales | Dog only | 3.866752955 | 0.009073666 |
| Cyanobacteria|Oxyphotobacteria|Nostocales|Phormidiaceae | Dog only | 3.751051232 | 0.013865594 |
| Cyanobacteria|Oxyphotobacteria|Nostocales|Phormidiaceae|Tychonema | Dog only | 3.737533822 | 0.013865594 |
| Cyanobacteria|Oxyphotobacteria|Nostocales|Phormidiaceae|Tychonema | Dog only | 3.75377887 | 0.013865594 |
| Cyanobacteria|Oxyphotobacteria|Nostocales|Phormidiaceae|Tychonema | Dog only | 3.739282645 | 0.013865594 |
| Firmicutes|Bacilli|Bacillales|Bacillaceae | Dog only | 3.695748042 | 0.021756601 |
| Fusobacteria | Dog only | 4.138062709 | 0.019258692 |
| Proteobacteria|Gammaproteobacteria|Pseudomonadales|Moraxellaceae|Acinetobacter | Dog only | 3.784002947 | 0.012279491 |
| Fusobacteria|Fusobacteriia | Dog only | 4.103032223 | 0.019258692 |
| Fusobacteria|Fusobacteriia|Fusobacteriales | Dog only | 4.099741856 | 0.019258692 |
| Actinobacteria|Actinobacteria|Corynebacteriales | No pets | 4.359962195 | 0.0000423 |
| Actinobacteria|Actinobacteria|Corynebacteriales|Corynebacteriaceae | No pets | 4.328252516 | 0.0000284 |
| Actinobacteria|Actinobacteria|Corynebacteriales|Corynebacteriaceae|Corynebacterium | No pets | 4.320687434 | 0.0000301 |
| Actinobacteria|Actinobacteria|Corynebacteriales|Corynebacteriaceae|Corynebacterium | No pets | 4.318757367 | 0.0000301 |
| Actinobacteria|Actinobacteria|Corynebacteriales|Corynebacteriaceae|Corynebacterium | No pets | 4.093373169 | 0.000136669 |
| Actinobacteria|Actinobacteria | No pets | 4.637371351 | 0.003464573 |
| Actinobacteria|Actinobacteria|Micrococcales|Micrococcaceae|Kocuria|Kocuria|Kocuria_marina | No pets | 4.135257728 | 0.005446549 |
| Actinobacteria|Actinobacteria|Class_Actinobacteria | No pets | 3.584582827 | 0.021228721 |
| Actinobacteria|Actinobacteria|Class_Actinobacteria | No pets | 3.590764509 | 0.021228721 |
| Actinobacteria|Actinobacteria|Class_Actinobacteria | No pets | 3.581543679 | 0.021228721 |
| Actinobacteria|Actinobacteria|Class_Actinobacteria | No pets | 3.585939613 | 0.021228721 |
| Actinobacteria|Actinobacteria|Class_Actinobacteria | No pets | 3.577696319 | 0.021228721 |
| Actinobacteria|Actinobacteria|Corynebacteriales|Corynebacteriaceae|Corynebacterium|Corynebacterium_casei | No pets | 3.592140738 | 0.029440363 |
| Firmicutes|Bacilli|Lactobacillales|Lactobacillaceae|Lactobacillus|Lactobacillus|Lactobacillus_gasseri | No pets | 3.631168071 | 0.013144465 |

**Table S9** Differentially abundant bacterial taxa in bed dust samples for homes with cat. †Column labeled “Pet ownership” represents the home that have cat in which the corresponding taxa (as presented in column labeled “Differentially abundant Taxa”), was found to be significantly differentially abundant by LEfSe. ‡ Differentially abundant taxa is described using the following hierarchy: Phylum|Class|Order|Family|Genus|species.

| **Taxa‡** | **Pet ownership†** | **LDA_Value** | **pvalue** |
| --- | --- | --- | --- |
| Actinobacteria|Actinobacteria|Corynebacteriales|Corynebacteriaceae|Turicella | Cat only | 3.687600228 | 0.00071901 |
| Actinobacteria|Actinobacteria|Corynebacteriales|Corynebacteriaceae|Turicella | Cat only | 3.682728396 | 0.00071901 |
| Actinobacteria|Actinobacteria|Corynebacteriales|Corynebacteriaceae|Turicella | Cat only | 3.673702628 | 0.00071901 |
| Cyanobacteria | No pets | 4.005392958 | 0.048908893 |
| Cyanobacteria|Oxyphotobacteria | No pets | 4.005392958 | 0.048908893 |
| Cyanobacteria|Oxyphotobacteria | No pets | 4.057428264 | 0.003782154 |

**Table S10. Differentially abundant fungal taxa in bed dust samples for homes with dog and cat both. †Column labeled “Pet ownership” represents the home that have dog and cat both in which the corresponding taxa (as presented in column labeled “Differentially abundant Taxa”), was found to be significantly differentially abundant by LEfSe. ‡ Differentially abundant taxa is described using the following hierarchy: Phylum|Class|Order|Family|Genus|species.**

| **Taxa‡** | **Pet ownership†** | **LDA_Value** | **pvalue** |
| --- | --- | --- | --- |
| Ascomycota|Dothideomycetes | Cat and Dog both | 4.838023838 | 0.01605383051 |
| Ascomycota|Dothideomycetes|Capnodiales|Teratosphaeriaceae | Cat and Dog both | 3.727717567 | 0.02388811493 |
| Ascomycota|Dothideomycetes|Capnodiales|Teratosphaeriaceae|Oleoguttula | Cat and Dog both | 3.711731768 | 0.02388811493 |
| Ascomycota|Dothideomycetes|Capnodiales|Teratosphaeriaceae|Oleoguttula|Oleoguttula_mirabilis | Cat and Dog both | 3.718029694 | 0.02388811493 |
| Ascomycota|Dothideomycetes|Class_Dothideomycetes | Cat and Dog both | 4.167521715 | 0.003209715217 |
| Ascomycota|Dothideomycetes|Class_Dothideomycetes|Class_Dothideomycetes | Cat and Dog both | 4.167542861 | 0.003209715217 |
| Ascomycota|Dothideomycetes|Class_Dothideomycetes|Class_Dothideomycetes|Class_Dothideomycetes | Cat and Dog both | 4.222216689 | 0.003209715217 |
| Ascomycota|Dothideomycetes|Class_Dothideomycetes|Class_Dothideomycetes|Class_Dothideomycetes|Class_Dothideomycetes | Cat and Dog both | 4.188591286 | 0.003209715217 |
| Ascomycota|Dothideomycetes|Pleosporales | Cat and Dog both | 4.821524751 | 0.02706597735 |
| Ascomycota|Dothideomycetes|Pleosporales|Leptosphaeriaceae | Cat and Dog both | 4.77212392 | 0.01094995455 |
| Ascomycota|Dothideomycetes|Pleosporales|Leptosphaeriaceae|Neophaeosphaeria | Cat and Dog both | 4.687002314 | 0.00002109685745 |
| Ascomycota|Dothideomycetes|Pleosporales|Leptosphaeriaceae|Neophaeosphaeria|Genus_Neophaeosphaeria | Cat and Dog both | 4.696152123 | 0.00002109685745 |
| Ascomycota|Dothideomycetes|Pleosporales|Sporormiaceae | Cat and Dog both | 4.18241649 | 0.0004892364346 |
| Ascomycota|Dothideomycetes|Pleosporales|Sporormiaceae|Preussia | Cat and Dog both | 4.164204452 | 0.0004892364346 |
| Ascomycota|Dothideomycetes|Pleosporales|Sporormiaceae|Preussia|Genus_Preussia | Cat and Dog both | 4.128487856 | 0.0004892364346 |
| Ascomycota|Sordariomycetes|Glomerellales | Cat and Dog both | 3.782566292 | 0.02388811493 |
| Ascomycota|Sordariomycetes|Glomerellales|Australiascaceae | Cat and Dog both | 3.7496855 | 0.02388811493 |
| Ascomycota|Sordariomycetes|Glomerellales|Australiascaceae|Monilochaetes|Monilochaetes_infuscans | Cat and Dog both | 3.655274614 | 0.02388811493 |
| Basidiomycota|Agaricomycetes|Atheliales | Cat and Dog both | 4.214223696 | 0.02388811493 |
| Basidiomycota|Agaricomycetes|Atheliales|Atheliaceae | Cat and Dog both | 4.157072921 | 0.02388811493 |
| Basidiomycota|Agaricomycetes|Atheliales|Atheliaceae|Tylospora | Cat and Dog both | 4.173623685 | 0.02388811493 |
| Basidiomycota|Agaricomycetes|Atheliales|Atheliaceae|Tylospora|Tylospora_fibrillosa | Cat and Dog both | 4.172115595 | 0.02388811493 |
| Chytridiomycota|Spizellomycetes|Spizellomycetales|Spizellomycetaceae|Spizellomyces | Cat and Dog both | 3.846990639 | 0.02310980132 |
| Chytridiomycota|Spizellomycetes|Spizellomycetales|Spizellomycetaceae|Spizellomyces|Spizellomyces_lactosolyticus | Cat and Dog both | 3.855976074 | 0.02310980132 |
| Kingdom_Fungi | Cat and Dog both | 3.731851084 | 0.02388811493 |
| Kingdom_Fungi | Cat and Dog both | 3.788921038 | 0.02388811493 |
| Kingdom_Fungi | Cat and Dog both | 3.605142701 | 0.02388811493 |
| Kingdom_Fungi | Cat and Dog both | 3.608830861 | 0.02388811493 |
| Kingdom_Fungi | Cat and Dog both | 3.749051563 | 0.02388811493 |
| Kingdom_Fungi | Cat and Dog both | 3.582836108 | 0.02388811493 |
| Kingdom_Fungi | Cat and Dog both | 3.830004957 | 0.02388811493 |
| Mortierellomycota | Cat and Dog both | 4.30574188 | 0.001782726779 |
| Mortierellomycota | Cat and Dog both | 4.316522702 | 0.001782726779 |
| Mortierellomycota|Mortierellomycetes | Cat and Dog both | 4.322749531 | 0.001782726779 |
| Mortierellomycota|Mortierellomycetes|Mortierellales | Cat and Dog both | 4.281985821 | 0.001782726779 |
| Mortierellomycota|Mortierellomycetes|Mortierellales|Mortierellaceae | Cat and Dog both | 4.317933367 | 0.001782726779 |
| Mortierellomycota|Mortierellomycetes|Mortierellales|Mortierellaceae|Mortierella | Cat and Dog both | 4.312624942 | 0.001782726779 |
| Mortierellomycota|Mortierellomycetes|Mortierellales|Mortierellaceae|Mortierella|Mortierella_reticulata | Cat and Dog both | 4.331395116 | 0.0005791568296 |

**Table S11. Differentially abundant fungal taxa in bed dust samples for homes with dog. †Column labeled “Pet ownership” represents the home that have dog in which the corresponding taxa (as presented in column labeled “Differentially abundant Taxa”), was found to be significantly differentially abundant by LEfSe. ‡ Differentially abundant taxa is described using the following hierarchy: Phylum|Class|Order|Family|Genus|species.**

| **Taxa‡** | **Pet ownership†** | **LDA_Value** | **pvalue** |
| --- | --- | --- | --- |
| Ascomycota|Dothideomycetes|Pleosporales|Pleosporaceae | Dog only | 3.948461972 | 0.02761306819 |
| Ascomycota|Dothideomycetes|Pleosporales|Pleosporaceae|Curvularia | Dog only | 3.684031824 | 0.01757844129 |
| Ascomycota|Dothideomycetes|Pleosporales|Pleosporaceae|Curvularia|Curvularia_ravenelii | Dog only | 3.681419641 | 0.01757844129 |
| Ascomycota|Dothideomycetes|Pleosporales|Pleosporaceae|Setosphaeria | Dog only | 3.641786113 | 0.01757844129 |
| Ascomycota|Dothideomycetes|Pleosporales|Pleosporaceae|Setosphaeria|Setosphaeria_monoceras | Dog only | 3.631156221 | 0.01757844129 |
| Ascomycota|Dothideomycetes|Pleosporales|Sporormiaceae | Dog only | 3.950163108 | 0.01757844129 |
| Ascomycota|Dothideomycetes|Pleosporales|Sporormiaceae|Preussia | Dog only | 3.856789327 | 0.01757844129 |
| Ascomycota|Dothideomycetes|Pleosporales|Sporormiaceae|Preussia|Genus_Preussia | Dog only | 3.895955539 | 0.01757844129 |
| Ascomycota|Pezizomycetes|Pezizales|Pezizaceae|Peziza | Dog only | 3.818563088 | 0.01757844129 |
| Ascomycota|Pezizomycetes|Pezizales|Pezizaceae|Peziza|Peziza_vesiculosa | Dog only | 3.830481511 | 0.01757844129 |
| Ascomycota|Saccharomycetes|Saccharomycetales|Debaryomycetaceae | Dog only | 4.00258977 | 0.0122794912 |
| Ascomycota|Saccharomycetes|Saccharomycetales|Debaryomycetaceae|Lodderomyces | Dog only | 4.00431893 | 0.0122794912 |
| Ascomycota|Saccharomycetes|Saccharomycetales|Debaryomycetaceae|Lodderomyces|Lodderomyces_elongisporus | Dog only | 3.966560082 | 0.0122794912 |
| Basidiomycota|Agaricomycetes|Boletales|Melanogastraceae | Dog only | 4.106736789 | 0.0122794912 |
| Basidiomycota|Agaricomycetes|Boletales|Melanogastraceae|Melanogaster | Dog only | 4.075976765 | 0.0122794912 |
| Basidiomycota|Agaricomycetes|Boletales|Melanogastraceae|Melanogaster|Melanogaster_rivularis | Dog only | 4.119351778 | 0.0122794912 |
| Basidiomycota|Ustilaginomycetes|Ustilaginales|Ustilaginaceae|Dirkmeia | Dog only | 3.831495167 | 0.01757844129 |
| Basidiomycota|Ustilaginomycetes|Ustilaginales|Ustilaginaceae|Dirkmeia|Dirkmeia_churashimaensis | Dog only | 3.750360167 | 0.01757844129 |
| Ascomycota|Sordariomycetes|Magnaporthales | No pets | 4.177503573 | 0.04321223653 |
| Ascomycota|Sordariomycetes|Magnaporthales|Magnaporthaceae | No pets | 4.185873217 | 0.04321223653 |
| Ascomycota|Sordariomycetes|Magnaporthales|Magnaporthaceae|Arxiella | No pets | 4.150122805 | 0.04849849268 |
| Ascomycota|Sordariomycetes|Magnaporthales|Magnaporthaceae|Arxiella|Arxiella_dolichandrae | No pets | 4.155868609 | 0.04849849268 |

**Table S12.** Differentially abundant fungal taxa in bed dust samples for homes with cat. †Column labeled “Pet ownership” represents the home that have cat in which the corresponding taxa (as presented in column labeled “Differentially abundant Taxa”), was found to be significantly differentially abundant by LEfSe. ‡ Differentially abundant taxa is described using the following hierarchy: Phylum|Class|Order|Family|Genus|species.

| **Taxa‡** | **Pet ownership†** | **LDA_Value** | **pvalue** |
| --- | --- | --- | --- |
| Ascomycota|Dothideomycetes|Capnodiales|Teratosphaeriaceae | Cat only | 4.1449 | 0.0033 |
| Ascomycota|Dothideomycetes|Capnodiales|Teratosphaeriaceae|Oleoguttula | Cat only | 4.1285 | 0.0033 |
| Ascomycota|Dothideomycetes|Capnodiales|Teratosphaeriaceae|Oleoguttula|Oleoguttula_mirabilis | Cat only | 4.1524 | 0.0033 |
| Ascomycota|Dothideomycetes|Pleosporales|Pleosporaceae|Curvularia | Cat only | 3.8399 | 0.0345 |
| Ascomycota|Dothideomycetes|Pleosporales|Pleosporaceae|Curvularia|Curvularia_ravenelii | Cat only | 3.8533 | 0.0345 |
| Ascomycota|Dothideomycetes|Pleosporales|Pleosporaceae|Setosphaeria | Cat only | 3.1776 | 0.0345 |
| Ascomycota|Dothideomycetes|Pleosporales|Pleosporaceae|Setosphaeria|Setosphaeria_monoceras | Cat only | 3.1145 | 0.0345 |
| Ascomycota|Lecanoromycetes|Lecanorales|Order_Lecanorales | Cat only | 4.0340 | 0.0091 |
| Ascomycota|Lecanoromycetes|Lecanorales|Order_Lecanorales|Order_Lecanorales | Cat only | 4.0330 | 0.0091 |
| Ascomycota|Lecanoromycetes|Lecanorales|Order_Lecanorales|Order_Lecanorales|Order_Lecanorales | Cat only | 4.0059 | 0.0091 |
| Ascomycota|Lecanoromycetes|Teloschistales|Teloschistaceae|Caloplaca | Cat only | 3.6424 | 0.0159 |
| Ascomycota|Lecanoromycetes|Teloschistales|Teloschistaceae|Caloplaca|Caloplaca_subalpina | Cat only | 3.6271 | 0.0159 |
| Ascomycota|Pezizomycetes|Pezizales|Pezizaceae|Peziza|Peziza_vesiculosa | Cat only | 3.1138 | 0.0345 |
| Basidiomycota|Agaricomycetes|Agaricales|Lyophyllaceae | Cat only | 3.0429 | 0.0302 |
| Basidiomycota|Agaricomycetes|Cantharellales|Clavulinaceae | Cat only | 3.2224 | 0.0028 |
| Basidiomycota|Agaricomycetes|Cantharellales|Clavulinaceae|Clavulina | Cat only | 3.2455 | 0.0028 |
| Basidiomycota|Agaricomycetes|Cantharellales|Clavulinaceae|Clavulina|Genus_Clavulina | Cat only | 3.2867 | 0.0028 |
| Basidiomycota|Ustilaginomycetes|Ustilaginales|Ustilaginaceae|Dirkmeia | Cat only | 3.0857 | 0.0345 |

**Table S13.** **.** Bacteria present in rural and urban environment at genus level.

| **Genera present in rural environment** | **Genera present in urban environment** |
| --- | --- |
| Anaerorhabdus_furcosa_group | Eubacterium_brachy_group |
| Clostridium_innocuum_group | Eubacterium_coprostanoligenes_group |
| Eubacterium_brachy_group | Eubacterium_eligens_group |
| Eubacterium_coprostanoligenes_group | Eubacterium_hallii_grou |
| Eubacterium_eligens_group | Eubacterium_nodatum_group |
| Eubacterium_hallii_group | Eubacterium_xylanophilum_group |
| Eubacterium_nodatum_group | Eubacterium_yurii_group |
| Eubacterium_ruminantium_group | Ruminococcus_gauvreauii_group |
| Eubacterium_xylanophilum_group | Ruminococcus_gnavus_group |
| Eubacterium_yurii_group | Ruminococcus_torques_group |
| Ruminococcus_gauvreauii_group | Abiotrophia |
| Ruminococcus_gnavus_group | Acaricomes |
| Ruminococcus_torques_group | Acetobacterium |
| Abiotrophia | Achromobacter |
| Acetitomaculum | Acidibacter |
| Acetivibrio | Acidiphilium |
| Acetobacter | Acidipila |
| Achromobacter | Acidocella |
| Acidibacter | Acidothermus |
| Acidiphilium | Acidovorax |
| Acidipila | Acinetobacter |
| Acidipropionibacterium | Actinobacillus |
| Acidisoma | Actinobaculum |
| Acidisphaera | Actinokineospora |
| Acidithiobacillaceae | Actinomyces |
| Acidocella | Actinomycetaceae |
| Acidothermus | Actinomycetospora |
| Acidovorax | Actinoplanes |
| Acinetobacter | Actinotalea |
| Actinobacillus | Actinotignum |
| Actinobaculum | Adhaeribacter |
| Actinocorallia | Aeribacillus |
| Actinomadura | Aerococcus |
| Actinomyces | Aeromicrobium |
| Actinomycetaceae | Aeromonas |
| Actinomycetospora | Aerosphaera |
| Actinoplanes | Aestuariimicrobium |
| Actinotalea | Aetherobacter |
| Actinotignum | Agathobacter |
| Adhaeribacter | Aggregatibacter |
| Advenella | Agrococcus |
| Aequorivita | Akkermansia |
| Aerococcus | Algoriphagus |
| Aeromicrobium | Aliicoccus |
| Aeromonas | Aliihoeflea |
| Aestuariimicrobium | Alishewanella |
| Aetherobacter | Alistipes |
| Agaricicola | Aliterella |
| Agathobacter | Alkalibacterium |
| Aggregatibacter | Alkanindiges |
| Agrococcus | Alloiococcus |
| Agromyces | Alloprevotella |
| Akkermansia | Rhizobium |
| Alcanivorax | Alloscardovia |
| Algoriella | Altererythrobacter |
| Algoriphagus | Alteromonas |
| Alicyclobacillus | Alysiella |
| Aliicoccus | Amaricoccus |
| Aliidiomarina | Ammoniphilus |
| Aliifodinibius | Amnibacterium |
| Aliihoeflea | Amycolatopsis |
| Alishewanella | Anaerobiospirillum |
| Alistipes | Anaerococcus |
| Aliterella | Anaerocolumna |
| Alkalibacterium | Anaerolinea |
| Alkaliphilus | Anaerolineae |
| Alkanindiges | Anaerosporobacter |
| Allofustis | Anaerostipes |
| Alloiococcus | Anaerovibrio |
| Alloprevotella | Aneurinibacillus |
| Rhizobium | Anoxybacillus |
| Alloscardovia | Antricoccus |
| Altererythrobacter | Aquicella |
| Alteromonas | Aquipuribacter |
| Alysiella | Arcticibacter |
| Amaricoccus | Arenibacter |
| Ammoniphilus | Arsenicicoccus |
| Amnibacterium | Arthrobacter |
| Amycolatopsis | Asticcacaulis |
| Anaerobacillus | Atopobium |
| Anaerobacterium | Atopostipes |
| Anaerobiospirillum | Aureimonas |
| Anaerococcus | Auritidibacter |
| Anaerocolumna | Azospirillum |
| Anaerolinea | Azotobacter |
| Anaerolineae | Bacillus |
| Anaerosinus | Bacteriovorax |
| Anaerostipes | Bacteroides |
| Anaerovibrio | Bavariicoccus |
| Anaerovorax | Bdellovibrio |
| Ancylobacter | Bdellovibrionaceae |
| Ancylothrix | Beijerinckiaceae |
| Anditalea | Belnapia |
| Aneurinibacillus | Bergeyella |
| Anoxybacillus | Bhargavaea |
| Antricoccus | Bifidobacterium |
| Aphanizomenon | Blastocatella |
| Apibacter | Blastocatellaceae |
| Aquabacterium | Blastococcus |
| Aquamicrobium | Blautia |
| Aquicella | Bordetella |
| Aquipuribacter | Bosea |
| Aquisphaera | Brachybacterium |
| Arcanobacterium | Brachymonas |
| Arcobacter | Bradyrhizobium |
| Arcticibacter | Brevibacillus |
| Arenibacter | Brevibacterium |
| Arenimonas | Brevundimonas |
| Aridibacter | Brochothrix |
| Arsenicicoccus | Bryobacter |
| Arthrobacter | Bryocella |
| Arthrospira | Buchnera |
| Asticcacaulis | Burkholderia_Caballeronia_Paraburkholderia |
| Atopobiaceae | Butyricicoccus |
| Atopobium | Caedibacter |
| Atopostipes | Caldicoprobacter |
| Aurantimonas | Calothrix |
| Aureimonas | Camelimonas |
| Azospirillum | Campylobacter |
| Bacillus | Candidatus_Alysiosphaera |
| Bacteriovorax | Candidatus_Amoebophilus |
| Bacteroides | Candidatus_Captivus |
| Bacteroidia_bacterium | Candidatus_Cardinium |
| Baia | Candidatus_Hamiltonella |
| Barrientosiimonas | Candidatus_Paracaedibacter |
| Bauldia | Candidatus_Rhabdochlamydia |
| Bavariicoccus | Candidatus_Saccharimonas |
| Bdellovibrio | Candidatus_Solibacter |
| Bdellovibrionaceae | Candidatus_Udaeobacter |
| Beijerinckiaceae | Candidatus_Xiphinematobacter |
| Belnapia | Capnocytophaga |
| Bergeyella | Cardiobacterium |
| Bhargavaea | Carnobacterium |
| Bifidobacterium | Catenibacterium |
| Bizionia | Catonella |
| Blastocatella | Caulobacter |
| Blastocatellaceae | Caulobacteraceae |
| Blastococcus | Cedecea |
| Blautia | Cellulomonas |
| Bogoriella | Cellulosilyticum |
| Bosea | Cellulosimicrobium |
| Brachybacterium | Cellvibrio |
| Brevibacillus | Centipeda |
| Brevibacterium | Cereibacter |
| Brevundimonas | Chalicogloea |
| Brochothrix | Chamaesiphon |
| Bryobacter | Chelativorans |
| Bryocella | Chiayiivirga |
| Buchnera | Chishuiella |
| Bulleidia | Chitinophaga |
| Burkholderia_Caballeronia_Paraburkholderia | Chloroflexaceae |
| Butyricicoccus | Chloroflexus |
| Caldicoprobacter | Christensenellaceae |
| Calothrix | Chroococcidiopsis |
| Calothrix | Chroococcidiopsis |
| Camelimonas | Chroococcidiopsis |
| Campylobacter | Chryseobacterium |
| Candidatus_Alysiosphaera | Chryseolinea |
| Candidatus_Amoebophilus | Chryseomicrobium |
| Candidatus_Ancillula | Chthoniobacter |
| Candidatus_Captivus | Chthoniobacteraceae |
| Candidatus_Cardinium | Chungangia |
| Candidatus_Chloroploca | Citrobacter |
| Candidatus_Endoecteinascidia | Class_Acidimicrobiia |
| Candidatus_Entotheonella | Class_Acidobacteria |
| Candidatus_Finniella | Class_Actinobacteria |
| Candidatus_Fritschea | Class_Actinobacteria |
| Candidatus_Jidaibacter | Class_Alphaproteobacteria |
| Candidatus_Megaira | Class_Bacilli |
| Candidatus_Microthrix | Class_Bacteroidia |
| Candidatus_Nostocoida | Class_Blastocatellia |
| Candidatus_Nucleicultrix | Class_Chloroflexi |
| Candidatus_Odyssella | Class_Chloroflexi |
| Candidatus_Paracaedibacter | Class_Chloroflexi |
| Candidatus_Regiella | Class_Gammaproteobacteria |
| Candidatus_Rhabdochlamydia | Class_Gracilibacteria |
| Candidatus_Saccharibacteria | Class_Oxyphotobacteria |
| Candidatus_Saccharimonas | Class_Thermoleophilia |
| Candidatus_Solibacter | Clavibacter |
| Candidatus_Trichorickettsia | Cloacibacterium |
| Candidatus_Udaeobacter | Clostridiales_Clostridiales |
| Candidatus_Uzinura | Clostridiales_Clostridiales |
| Candidatus_Xiphinematobacter | Clostridiisalibacter |
| Candidimonas | Clostridioides |
| Capnocytophaga | Clostridium_sensu_stricto |
| Caproiciproducens | Cnuella |
| Cardiobacterium | Cobetia |
| Carnobacterium | Cohnella |
| Catenibacterium | Collinsella |
| Catenuloplanes | Comamonas |
| Catonella | Conexibacter |
| Caulobacter | Coprococcus |
| Caulobacteraceae | Corallococcus |
| Cellulomonas | Corynebacterium |
| Cellulosilyticum | Craurococcus |
| Cellulosimicrobium | Cruoricaptor |
| Cellvibrio | Cryptobacterium |
| Cereibacter | Cupriavidus |
| Cesiribacter | Curtobacterium |
| Cetobacterium | Cuspidothrix |
| Chalicogloea | Cutibacterium |
| Chamaesiphon | Cystobacter |
| Chelatococcus | Cytophaga |
| Chiayiivirga | Dactylosporangium |
| Chishuiella | Dapisostemonum |
| Chloroflexaceae | Defluviicoccus |
| Chloroflexus | Deinococcus |
| Chloronema | Delftia |
| Christensenellaceae | Demequina |
| Chroococcidiopsis | Dermabacter |
| Chroococcidiopsis | Dermacoccus |
| Chroococcidiopsis | Desemzia |
| Chryseobacterium | Desulfosporosinus |
| Chryseolinea | Desulfurispora |
| Chryseomicrobium | Devosia |
| Chthoniobacter | Dialister |
| Chthoniobacteraceae | Diaphorobacter |
| Citrobacter | Dietzia |
| Class_Acidimicrobiia | Dokdonella |
| Class_Acidimicrobiia | Dolosigranulum |
| Class_Acidimicrobiia | Domibacillus |
| Class_Acidimicrobiia | Dorea |
| Class_Actinobacteria | Duganella |
| Class_Actinobacteria | Dyadobacter |
| Class_Actinobacteria | Dyella |
| Class_Alphaproteobacteria | Edaphobacter |
| Class_Bacilli | Eggerthella |
| Class_Bacteroidia | Eggerthellaceae |
| Class_Blastocatellia | Eikenella |
| Class_Chloroflexi | Empedobacter |
| Class_Chloroflexi | Endobacter |
| Class_Chloroflexi | endosymbionts8 |
| Class_Chloroflexi | Enhydrobacter |
| Class_Chloroflexi | Enteractinococcus |
| Class_Clostridia | Enterobacter |
| Class_Deltaproteobacteria | Enterococcus |
| Class_Elusimicrobia | Epulopiscium |
| Class_Gammaproteobacteria | Eremococcus |
| Class_Gracilibacteria | Erwinia |
| Class_Oxyphotobacteria | Erysipelatoclostridium |
| Class_Patescibacteria | Erysipelothrix |
| Class_S0134_terrestrial_group | Erysipelotrichaceae |
| Class_Sericytochromatia | Erysipelotrichaceae |
| Class_Thermoleophilia | Erythrobacter |
| Clavibacter | Escherichia_Shigella |
| Cloacibacterium | Exiguobacterium |
| Clostridiales | Ezakiella |
| Clostridiales | Facklamia |
| Clostridiales | Faecalibacterium |
| Clostridiisalibacter | Falsirhodobacter |
| Clostridioides | Family_Acetobacteraceae |
| Clostridium_sensu_stricto | Family_Acidobacteriaceae |
| Cnuella | Family_Actinomycetaceae |
| Cobetia | Family_Aerococcaceae |
| Cohnella | Family_Alteromonadaceae |
| Collinsella | Family_Ammopiptanthus_mongolicus |
| Comamonas | Family_Anaerolineaceae |
| Conchiformibius | Family_Anaerolineae |
| Conexibacter | Family_Archangiaceae |
| Confluentibacter | Family_Ardenticatenaceae |
| Coprococcus | Family_Athetis_lepigone |
| Corallococcus | Family_Auxenochlorella |
| Corticibacter | Family_Bacillaceae |
| Corticibacterium | Family_Bacillaria_paxillifer |
| Corynebacterium | Family_bacteriap25 |
| Craurococcus | Family_Beijerinckiaceae |
| Crenothrix | Family_Beutenbergiaceae |
| Crinalium | Family_Bifidobacteriaceae |
| Crossiella | Family_Blastocatellaceae |
| Cruoricaptor | Family_Bogoriellaceae |
| Cryptosporangium | Family_Brevibacteriaceae |
| Curtobacterium | Family_Bryum_argenteum |
| Cutibacterium | Family_Burkholderiaceae |
| Cytophaga | Family_Butomus_umbellatus |
| Dactylosporangium | Family_Caldilineaceae |
| Dapisostemonum | Family_candidate |
| Defluviicoccus | Family_Candidatus_Saccharibacteria |
| Defluviitaleaceae | Family_Carnobacteriaceae |
| Deinococcus | Family_Caulobacteraceae |
| Delftia | Family_Cellvibrionaceae |
| Demequina | Family_Cercis_gigantea |
| Dermabacter | Family_Chitinophagaceae |
| Dermacoccus | Family_Chloroflexaceae |
| Desemzia | Family_Chroococcidiopsaceae |
| Desulfitibacter | Family_Clostridiaceae |
| Desulfosporosinus | Family_Clostridiales |
| Desulfotomaculum | Family_Coccomyxa_simplex |
| Desulfovibrio | Family_Corynebacteriaceae |
| Dethiobacter | Family_Cyclobacteriaceae |
| Devosia | Family_Cytophagaceae |
| Dialister | Family_Cytophagales |
| Diaphorobacter | Family_Demequinaceae |
| Dielma | Family_Dermacoccaceae |
| Dietzia | Family_Dermatophilaceae |
| Diplorickettsia | Family_Desmochloris_halophila |
| Dokdonella | Family_Devosiaceae |
| Dolosigranulum | Family_Diplorickettsiaceae |
| Domibacillus | Family_Edaphochlorella_mirabilis |
| Dorea | Family_Enterobacteriaceae |
| Duganella | Family_Enterococcaceae |
| Dyadobacter | Family_Eosphagnum |
| Dyella | Family_Ettlia_pseudoalveolaris |
| Edaphobacter | Family_Eubacteriaceae |
| Eggerthella | Family_Fimbriimonadaceae |
| Eggerthellaceae | Family_Flavobacteriaceae |
| Eggerthia | Family_Folsomia_candida |
| Eikenella | Family_Geminicoccaceae |
| Eisenbergiella | Family_Gemmatimonadaceae |
| Elioraea | Family_Geodermatophilaceae |
| Empedobacter | Family_Ginkgo_biloba |
| Emticicia | Family_Helianthus_annuus |
| Endobacter | Family_Heveochlorella_hainangensis |
| endosymbionts8 | Family_Hydra_vulgaris |
| Enhydrobacter | Family_Ilumatobacteraceae |
| Enteractinococcus | Family_Intrasporangiaceae |
| Enterobacter | Family_Kallotenuales |
| Enterococcus | Family_Kineosporiaceae |
| Enterorhabdus | Family_Kuruna_debilis |
| Epulopiscium | Family_Lachnospiraceae |
| Eremococcus | Family_Lactobacillales |
| Erwinia | Family_Lathyrus_tingitanus |
| Erysipelatoclostridium | Family_Leptotrichiaceae |
| Erysipelothrix | Family_Limnochordaceae |
| Erysipelotrichaceae | Family_Linum_usitatissimum |
| Erysipelotrichaceae | Family_Longimicrobiaceae |
| Erythrobacter | Family_marine_metagenome |
| Escherichia_Shigella | Family_Medicago_truncatula |
| Euzebyella | Family_Methyloligellaceae |
| Exiguobacterium | Family_Methylophilaceae |
| Ezakiella | Family_Microbacteriaceae |
| Facklamia | Family_Micrococcaceae |
| Faecalibacterium | Family_Micromonosporaceae |
| Faecalitalea | Family_Microscillaceae |
| Falsirhodobacter | Family_Midichloriaceae |
| Family_Acetobacteraceae | Family_Moraxellaceae |
| Family_Acidobacteriaceae | Family_Muribaculaceae |
| Family_actinobacterium | Family_Myxococcaceae |
| Family_Actinomycetaceae | Family_Myxococcales |
| Family_Aerococcaceae | Family_Myxococcales |
| Family_Alteromonadaceae | Family_Myxococcales |
| Family_Anaerolineaceae | Family_Neisseriaceae |
| Family_Anaerolineae | Family_Neocystis_brevis |
| Family_Arachis_hypogaea | Family_Nephrolepis_biserrata |
| Family_Archangiaceae | Family_Nocardiaceae |
| Family_Ardenticatenaceae | Family_Nocardioidaceae |
| Family_Athetis_lepigone | Family_Nostocaceae |
| Family_Atopobiaceae | Family_Oligoflexales |
| Family_Azolla_filiculoides | Family_Opitutaceae |
| Family_Azospirillaceae | Family_Paenibacillaceae |
| Family_Babeliales | Family_Paracaedibacteraceae |
| Family_Bacillaceae | Family_Paradoxia_multiseta |
| Family_Bacillaria_paxillifer | Family_Pardosa_pseudoannulata |
| Family_bacteriap25 | Family_Pasteurellaceae |
| Family_Balneolaceae | Family_Peptostreptococcaceae |
| Family_Beijerinckiaceae | Family_Phaseolus_acutifolius |
| Family_Betaproteobacteriales | Family_Phytophthora_lateralis |
| Family_Beutenbergiaceae | Family_Picea_glauca |
| Family_Bifidobacteriaceae | Family_Planctonema_lauterbornii |
| Family_Blastocatellaceae | Family_Planococcaceae |
| Family_Bogoriellaceae | Family_Polyangiaceae |
| Family_Bracteacoccus_giganteus | Family_Prasiola_crispa |
| Family_Bradymonadaceae | Family_Prevotellaceae |
| Family_Bryum_argenteum | Family_Prolixibacteraceae |
| Family_Burkholderiaceae | Family_Propionibacteriaceae |
| Family_Butomus_umbellatus | Family_Pseudochlorella_signiensis |
| Family_Caldilineaceae | Family_Pseudomonadaceae |
| Family_Camellia_sinensis | Family_Pseudomuriella_schumacherensis |
| Family_candidate | Family_Pseudonocardiaceae |
| Family_Candidatus_Saccharibacteria | Family_Pseudotaxus_chienii |
| Family_Capsicum_annuum | Family_Rhizobiaceae |
| Family_Cardiobacteriaceae | Family_Rhizobiales |
| Family_Carnobacteriaceae | Family_Rhizobiales |
| Family_Carteria | Family_Rhodanobacteraceae |
| Family_Caulobacteraceae | Family_Rhodobacteraceae |
| Family_Cellulomonadaceae | Family_Rhodothermaceae |
| Family_Cellvibrionaceae | Family_Rickettsiaceae |
| Family_Chitinophagaceae | Family_Rickettsiales |
| Family_Chlamydiales_ | Family_Roseiflexaceae |
| Family_Chloroflexaceae | Family_Ruminococcaceae |
| Family_Chloromonas_perforata | Family_Saccharimonadaceae |
| Family_Chloromonas_radiata | Family_Saccharimonadales |
| Family_Chlorosarcina_brevispinosa | Family_Saccharum_hybrid_cultivar |
| Family_Christensenellaceae | Family_Sandaracinaceae |
| Family_Chromochloris_zofingiensis | Family_Saprospiraceae |
| Family_Chroococcidiopsaceae | Family_Senna_alexandrina |
| Family_Chthoniobacteraceae | Family_Solirubrobacteraceae |
| Family_Clostridiaceae | Family_Solirubrobacterales |
| Family_Clostridiales | Family_Sphingobacteriaceae |
| Family_Clostridiales | Family_Sphingobacteriales |
| Family_Clostridiales | Family_Sphingomonadaceae |
| Family_Coccomyxa | Family_Spirosomaceae |
| Family_Coccomyxa | Family_Sporichthyaceae |
| Family_Coelastrella | Family_SR1 |
| Family_Coleofasciculaceae | Family_Staphylococcaceae |
| Family_Corynebacteriaceae | Family_Steroidobacteraceae |
| Family_Cyanobacteriaceae | Family_Streptomycetaceae |
| Family_Cyclobacteriaceae | Family_Tannerellaceae |
| Family_Cytophagaceae | Family_Tepidisphaeraceae |
| Family_Cytophagales | Family_Tepidisphaerales |
| Family_Demequinaceae | Family_Tetradesmus_obliquus |
| Family_Dermabacteraceae | Family_Thermoactinomycetaceae |
| Family_Dermacoccaceae | Family_Thermomicrobiales |
| Family_Dermatophilaceae | Family_Thermomicrobiales |
| Family_Desmochloris_halophila | Family_Triticum_polonicum |
| Family_Devosiaceae | Family_Veillonellaceae |
| Family_Diplorickettsiaceae | Family_Verrucomicrobiales |
| Family_Edaphochlorella_mirabilis | Family_Watanabea_reniformis |
| Family_Elliptochloris_bilobata | Family_Weeksellaceae |
| Family_Enterobacteriaceae | Family_Xanthobacteraceae |
| Family_Enterococcaceae | Family_Xanthomonadaceae |
| Family_Entotheonellaceae | Family_Xylochloris_irregularis |
| Family_Eosphagnum | Fastidiosipila |
| Family_Erysipelotrichaceae | Faucicola |
| Family_Ettlia_pseudoalveolaris | Ferruginibacter |
| Family_Euzebyaceae | Fibrella |
| Family_FFCH9454 | Fictibacillus |
| Family_Fibrobacteraceae | Filifactor |
| Family_Fibrobacterales | Finegoldia |
| Family_Fimbriimonadaceae | Flavihumibacter |
| Family_Flavobacteriaceae | Flavisolibacter |
| Family_Geminicoccaceae | Flavobacterium |
| Family_Gemmataceae | Fonticella |
| Family_Gemmatimonadaceae | Franconibacter |
| Family_Geodermatophilaceae | Frederiksenia |
| Family_Geranium_phaeum | Friedmanniella |
| Family_Helianthus_annuus | Frondihabitans |
| Family_Heliobacteriaceae | Fusicatenibacter |
| Family_Heveochlorella_hainangensis | Fusobacterium |
| Family_Hydra_vulgaris | Gaiella |
| Family_Hydrogenophilaceae | Galbitalea |
| Family_Hymenobacteraceae | Gallicola |
| Family_Ilumatobacteraceae | Gardnerella |
| Family_Intrasporangiaceae | Gelidibacter |
| Family_Isosphaeraceae | Gemella |
| Family_Kallotenuales | Geminicoccus |
| Family_Kineosporiaceae | Gemmatimonas |
| Family_Ktedonobacteraceae | Gemmatirosa |
| Family_Kuruna_debilis | Gemmobacter |
| Family_Lachnospiraceae | Geobacillus |
| Family_Lactobacillaceae | Geodermatophilus |
| Family_Lactobacillales | Georgenia |
| Family_Lathyrus_tingitanus | Gilliamella |
| Family_Lens_culinaris_lentil | Gillisia |
| Family_Leptolyngbyaceae | Globicatella |
| Family_Leptotrichiaceae | Gluconobacter |
| Family_Limnochordaceae | Glutamicibacter |
| Family_Linum_usitatissimum_flax | Gordonia |
| Family_Longimicrobiaceae | Gracilibacter |
| Family_marine_metagenome | Gramella |
| Family_Marinifilaceae | Granulicatella |
| Family_Marinilabiliaceae | Granulicatella |
| Family_Medicago_truncatula | Granulicella |
| Family_Methylacidiphilaceae | Haematobacter |
| Family_Methyloligellaceae | Haemophilus |
| Family_Methylomonaceae | Hahella |
| Family_Methylophagaceae | Haliangium |
| Family_Methylophilaceae | Halomonas |
| Family_Methylopilaceae | Haloplasma |
| Family_Micavibrionaceae | Haoranjiania |
| Family_Microbacteriaceae | Helcobacillus |
| Family_Micrococcaceae | Helcococcus |
| Family_Micrococcales | Herbinix |
| Family_Micromonosporaceae | Herpetosiphon |
| Family_Micropepsaceae | Hirschia |
| Family_Microscillaceae | Holdemanella |
| Family_Microtrichaceae | Howardella |
| Family_Monodopsis | Huanghella |
| Family_Moraxellaceae | Hungatella |
| Family_Muribaculaceae | Hydrogenispora |
| Family_Myxococcaceae | Hymenobacter |
| Family_Myxococcales | Iamia |
| Family_Myxococcales | Ignavigranum |
| Family_Myxococcales | Ilumatobacter |
| Family_Neisseriaceae | Ilumatobacteraceae |
| Family_Neocystis_brevis | Intestinibacter |
| Family_Nephrolepis_biserrata | Isoptericola |
| Family_Nocardiaceae | Izhakiella |
| Family_Nocardioidaceae | Janthinobacterium |
| Family_Nostocaceae | Jatrophihabitans |
| Family_Nostocales_Incertae_Sedis | Jeotgalibaca |
| Family_Oligoflexaceae | Jeotgalicoccus |
| Family_Oligoflexales | Johnsonella |
| Family_Opitutaceae | Jonquetella |
| Family_Oxyphotobacteria_Incertae_Sedis | Kallotenue |
| Family_Paenibacillaceae | Kineococcus |
| Family_Paludibacteraceae | Kineosporia |
| Family_Paracaedibacteraceae | Kingdom_Bacteria |
| Family_Parachlamydiaceae | Kingella |
| Family_Paradoxia_multiseta | Klebsiella |
| Family_Pardosa_pseudoannulata | Kocuria |
| Family_Pasteurellaceae | Komagataeibacter |
| Family_Pedosphaeraceae | Kosakonia |
| Family_Peptococcaceae | Kurthia |
| Family_Peptostreptococcaceae | Kushneria |
| Family_Phaseolus_acutifolius | Kytococcus |
| Family_Phormidiaceae | Laceyella |
| Family_Phytophthora_lateralis | Lachnoanaerobaculum |
| Family_Picea_glauca | Lachnoclostridium |
| Family_Pirellulaceae | Lachnospira |
| Family_Planctonema_lauterbornii | Lachnospiraceae |
| Family_Planococcaceae | Lachnospiraceae |
| Family_Planoglabratella_opercularis | Lachnospiraceae |
| Family_Polyangiaceae | Lachnospiraceae |
| Family_Prevotellaceae | Lachnospiraceae |
| Family_Propionibacteriaceae | Lachnospiraceae |
| Family_Pseudochlorella_signiensis | Lachnospiraceae |
| Family_Pseudomonadaceae | Lachnospiraceae |
| Family_Pseudomuriella_schumacherensis | Lactobacillus |
| Family_Pseudonocardiaceae | Lactococcus |
| Family_Pseudotaxus_chienii | Lacunisphaera |
| Family_Ptilocladiopsis_horrida | Larkinella |
| Family_Rhizobiaceae | Lautropia |
| Family_Rhizobiales | Lawsonella |
| Family_Rhizobiales | Legionella |
| Family_Rhizobiales_Incertae_Sedis | Leptolyngbya |
| Family_Rhodanobacteraceae | Leptolyngbya |
| Family_Rhodobacteraceae | Leptolyngbya |
| Family_Rhodocyclaceae | Leptolyngbyaceae |
| Family_Rhodothermaceae | Leptotrichia |
| Family_Rickettsiaceae | Leucobacter |
| Family_Rickettsiales | Leuconostoc |
| Family_Rickettsiales | Lewinella |
| Family_Roseiflexaceae | Lihuaxuella |
| Family_Ruminococcaceae | Limnobacter |
| Family_Saccharimonadaceae | Longimicrobium |
| Family_Saccharimonadales | Lunatimonas |
| Family_Sandaracinaceae | Luteibacter |
| Family_Saprospiraceae | Luteimonas |
| Family_Senna_alexandrina | Luteococcus |
| Family_Simkaniaceae | Luteolibacter |
| Family_Sneathiellaceae | Lysinibacillus |
| Family_Solibacteraceae | Lysobacter |
| Family_Solirubrobacteraceae | Macrococcus |
| Family_Solirubrobacterales | Mannheimia |
| Family_Sphingobacteriaceae | Marinilactibacillus |
| Family_Sphingobacteriales | Marinobacter |
| Family_Sphingobacteriales | Marinomonas |
| Family_Sphingomonadaceae | Marmoricola |
| Family_Spirosomaceae | Massilia |
| Family_Sporichthyaceae | Megamonas |
| Family_SR1_bacterium | Megasphaera |
| Family_Staphylococcaceae | Meiothermus |
| Family_Steroidobacteraceae | Melittangium |
| Family_Streptomycetaceae | Mesorhizobium |
| Family_Symbiochloris_handae | Methylobacterium |
| Family_Syntrophomonadaceae | Methylocella |
| Family_Tannerellaceae | Methylotenera |
| Family_Tepidisphaeraceae | Methyloversatilis |
| Family_Tepidisphaerales | Micavibrio |
| Family_Tetradesmus_obliquus | Microbacterium |
| Family_Thermoactinomycetaceae | Micrococcus |
| Family_Thermoanaerobacteraceae | Microcoleus |
| Family_Thermomicrobiales | Microlunatus |
| Family_Thermomicrobiales | Micromonospora |
| Family_Trebouxia | Micropruina |
| Family_Triticum_aestivum | Microtrichaceae |
| Family_Triticum_polonicum | Microvirga |
| Family_Vaucheria_litorea | Mobilicoccus |
| Family_Veillonellaceae | Mobiluncus |
| Family_Vermiphilaceae | Modestobacter |
| Family_Verrucomicrobiaceae | Mogibacterium |
| Family_Vibrionaceae | Moheibacter |
| Family_Virgulinella_fragilis | Moraxella |
| Family_Watanabea_reniformis | Moritella |
| Family_Weeksellaceae | Moryella |
| Family_Xanthobacteraceae | Mucilaginibacter |
| Family_Xanthomonadaceae | Murdochiella |
| Family_Xylochloris_irregularis | Muricoccus |
| Fastidiosipila | Mycetocola |
| Faucicola | Mycobacterium |
| Ferruginibacter | Mycoplasma |
| Fibrella | Myxococcus |
| Fictibacillus | Nakamurella |
| Filifactor | Nannocystis |
| Finegoldia | Natribacillus |
| Flaviaesturariibacter | Negativicoccus |
| Flaviflexus | Neisseria |
| Flavihumibacter | Neochlamydia |
| Flavimarina | Neomicrococcus |
| Flavisolibacter | Nesterenkonia |
| Flavitalea | Niabella |
| Flavobacterium | Nitrospira |
| Flavonifractor | Niveispirillum |
| Flexibacter | Nocardioides |
| Fluviicola | Nocardiopsis |
| Fonticella | Nodosilinea |
| Frederiksenia | Nosocomiicoccus |
| Friedmanniella | Nostoc |
| Frondihabitans | Nostoc |
| Fusibacter | Nostoc |
| Fusicatenibacter | Noviherbaspirillum |
| Fusobacterium | Novosphingobium |
| Gaiella | Nubsella |
| Galbibacter | Oceanivirga |
| Gallicola | Oceanobacillus |
| Garciella | Ochrobactrum |
| Gardnerella | Oerskovia |
| Garicola | Ohtaekwangia |
| Gelidibacter | Oligella |
| Gemella | Oligoflexus |
| Geminicoccus | Olivibacter |
| Gemmatimonas | Olsenella |
| Gemmatirosa | Opitutus |
| Gemmobacter | Order_Absconditabacteriales |
| Geobacillus | Order_Acidimicrobiia |
| Geodermatophilus | Order_Actinobacteria |
| Georgenia | Order_Actinomarinales |
| Gilliamella | Order_Actinomycetales |
| Gillisia | Order_Anaerolineae |
| Gleocapsa | Order_Anaerolineae |
| Globicatella | Order_Armatimonadales |
| Gloeocapsa | Order_Bacillales |
| Glutamicibacter | Order_Bacteroidales |
| Glycomyces | Order_Betaproteobacteriales |
| Gordonia | Order_Blastocatellia |
| Granulicatella | Order_Chloroplast |
| Granulicatella | Order_Clostridiales |
| Granulicella | Order_Coriobacteriales |
| Gryllotalpicola | Order_Corynebacteriales |
| Haematobacter | Order_Frankiales |
| Haemophilus | Order_Gaiellales |
| Hahella | Order_Gammaproteobacteria |
| Haliangium | Order_Gracilibacteria |
| Halocella | Order_Ignavibacteria |
| Halomonas | Order_Ktedonobacteria |
| Haloplasma | Order_Lactobacillales |
| Hansschlegelia | Order_Micavibrionales |
| Helcobacillus | Order_Micrococcales |
| Helcococcus | Order_Microtrichales |
| Herbaspirillum | Order_Mollicutes |
| Herbinix | Order_Myxococcales |
| Herpetosiphon | Order_Nostocales |
| Holdemanella | Order_Pseudomonadales |
| Howardella | Order_Rhizobiales |
| Huanghella | Order_Rhodospirillales |
| Hydrogenispora | Order_Rickettsiales |
| Hymenobacter | Order_Saccharimonadales |
| Hyphomicrobium | Order_Thalassobaculales |
| Hyphomonadaceae | Oribacterium |
| Iamia | Ornithinicoccus |
| Idiomarina | Ornithinimicrobium |
| Ignavigranum | Ornithobacterium |
| Ilumatobacter | Ottowia |
| Ilumatobacteraceae | Oxyphotobacteria_Incertae_Sedis |
| Immundisolibacter | Paenibacillus |
| Inquilinus | Paeniclostridium |
| Intestinibacter | Paeniglutamicibacter |
| Intestinimonas | Paenisporosarcina |
| Isoptericola | Pajaroellobacter |
| Izhakiella | Paludibacteraceae |
| Jahnella | Paludibaculum |
| Jannaschia | Pantoea |
| Janthinobacterium | Parabacteroides |
| Jatrophihabitans | Paraburkholderia_tropica |
| Jeotgalibaca | Paracoccus |
| Jeotgalicoccus | Parapedobacter |
| Johnsonella | Parasutterella |
| Jonesia | Parvibaculum |
| Jonquetella | Parvimonas |
| Kallotenue | Pasteurella |
| Kandleria | Patulibacter |
| Kineococcus | Pedobacter |
| Kineosporia | Pelagibacterium |
| Kingdom_Bacteria | Peptoclostridium |
| Kingella | Peptococcus |
| Klebsiella | Peptoniphilus |
| Kocuria | Peptostreptococcus |
| Kosakonia | Peredibacter |
| Krasilnikovia | Persicitalea |
| Kribbella | Phascolarctobacterium |
| Kriegella | Phaselicystis |
| Ktedonobacter | Phenylobacterium |
| Kurthia | Phormidesmis |
| Kushneria | Phormidesmis |
| Kytococcus | Phormidium |
| Laceyella | Photobacterium |
| Lachnoanaerobaculum | Phycisphaeraceae |
| Lachnoclostridium | Phylum_Actinobacteria |
| Lachnospira | Phylum_BRC1 |
| Lachnospiraceae | Phylum_FBP |
| Lachnospiraceae | Phylum_Firmicutes |
| Lachnospiraceae | Phylum_Proteobacteria |
| Lachnospiraceae | Phylum_WPS-2 |
| Lachnospiraceae | Pigmentiphaga |
| Lachnospiraceae | Pilimelia |
| Lachnospiraceae | Pirellulaceae |
| Lachnospiraceae | Piscicoccus |
| Lactobacillus | Pisciglobus |
| Lactococcus | Planifilum |
| Lacunisphaera | Planktothricoides |
| Lapillicoccus | Planktothrix |
| Larkinella | Planococcus |
| Lautropia | Planomicrobium |
| Lawsonella | Pleurocapsa |
| LB3_76 | Polaromonas |
| Lechevalieria | Polyangium |
| Legionella | Polymorphobacter |
| Leptolyngbya | Pontibacter |
| Leptolyngbya | Porphyrobacter |
| Leptolyngbya | Porphyromonas |
| Leptolyngbya | Prevotella |
| Leptotrichia | Procabacter |
| Leucobacter | Promicromonospora |
| Leuconostoc | Propioniciclava |
| Levilinea | Propionicimonas |
| Lihuaxuella | Propionimicrobium |
| Limnobacter | Proteiniclasticum |
| Limnochorda | Pseudactinotalea |
| Listeria | Pseudarthrobacter |
| Loktanella | Pseudoalteromonas |
| Longimicrobium | Pseudoclavibacter |
| Luedemannella | Pseudoflavitalea |
| Luteibacter | Pseudofulvimonas |
| Luteimonas | Pseudoglutamicibacter |
| Luteococcus | Pseudokineococcus |
| Luteolibacter | Pseudomonas |
| Lutispora | Pseudonocardia |
| Lysinibacillus | Pseudopropionibacterium |
| Lysobacter | Pseudorhodobacter |
| Macrococcus | Pseudorhodoferax |
| Mannheimia | Pseudorhodoplanes |
| Marinactinospora | Pseudosphingobacterium |
| Marinilactibacillus | Pseudoxanthomonas |
| Marinilutecoccus | Psychrobacillus |
| Marinobacter | Psychrobacter |
| Marinococcus | Psychroflexus |
| Marmoricola | Psychroglaciecola |
| Marvinbryantia | Pusillimonas |
| Massilia | Pyrinomonadaceae |
| Mastigocladopsis | Qipengyuania |
| Megamonas | Quadrisphaera |
| Megasphaera | Rahnella |
| Melittangium | Rathayibacter |
| Methylobacterium | Reyranella |
| Methylocaldum | Rheinheimera |
| Methylocella | Rhizobacter |
| Methylopila | Rhizorhapis |
| Methylorosula | Rhodanobacter |
| Methylotenera | Rhodococcus |
| Micavibrio | Rhodocytophaga |
| Microbacterium | Rhodoferax |
| Micrococcus | Rhodopseudomonas |
| Microcoleus | Rhodovarius |
| Microcoleus | Rhodovastum |
| Microlunatus | Rickettsia |
| Micromonospora | Rickettsiella |
| Micropruina | Romboutsia |
| Microtrichaceae | Roseateles |
| Microtrichaceae | Roseburia |
| Microvirga | Roseomonas |
| Millisia | Rothia |
| Mobilicoccus | Rubellimicrobium |
| Mobilitalea | Rubritepida |
| Mobiluncus | Rubrivirga |
| Modestobacter | Rubrobacter |
| Mogibacterium | Rufibacter |
| Moheibacter | Ruminiclostridium |
| Moraxella | Ruminococcaceae |
| Morganella | Ruminococcaceae |
| Moryella | Ruminococcaceae |
| Motilibacter | Ruminococcaceae |
| Mucilaginibacter | Ruminococcaceae |
| Murdochiella | Ruminococcus |
| Muricoccus | Rummeliibacillus |
| Myceligenerans | Saccharibacillus |
| Mycobacterium | Saccharopolyspora |
| Mycoplasma | Salana |
| Myroides | Salegentibacter |
| Myxococcales | Salinicoccus |
| Myxococcus | Salinicola |
| Nakamurella | Salinimicrobium |
| Nannocystis | Salinivibrio |
| Natribacillus | Salipaludibacillus |
| Negativicoccus | Sandaracinobacter |
| Neisseria | Sanguibacter |
| Neorickettsia | Sarcina |
| Nesterenkonia | Scardovia |
| Niabella | Scytonema |
| Nibribacter | Scytonema |
| Nitratireductor | Sedimentibacter |
| Nitriliruptor | Segetibacter |
| Nitrolancea | Selenomonas |
| Nitrosococcus | Senegalimassilia |
| Nitrosomonas | Serratia |
| Nitrosospira | Shewanella |
| Nitrospira | Shimazuella |
| Nocardioides | Shimwellia |
| Nocardiopsis | Shinella |
| Nodosilinea | Shuttleworthia |
| Nosocomiicoccus | Siccibacter |
| Nostoc | Simonsiella |
| Nostoc | Singulisphaera |
| Nostoc | Sinobaca |
| Novibacillus | Skermanella |
| Noviherbaspirillum | Sneathia |
| Novosphingobium | Snodgrassella |
| Nubsella | Solibacillus |
| Oceanivirga | Solirubrobacter |
| Oceanobacillus | Solobacterium |
| Ochrobactrum | Soonwooa |
| Oerskovia | Sorangium |
| Ohtaekwangia | Sphingobacterium |
| Oleiagrimonas | Sphingobium |
| Oligella | Sphingomonadaceae |
| Oligoflexus | Sphingomonadaceae |
| Olivibacter | Sphingomonadaceae |
| Olsenella | Sphingomonadaceae |
| Order_Absconditabacteriales | Sphingomonas |
| Order_Acidimicrobiia | Sphingopyxis |
| Order_Acidobacteria | Sphingorhabdus |
| Order_Actinobacteria | Spirosoma |
| Order_Actinomarinales | Sporocytophaga |
| Order_Actinomycetales | Sporosarcina |
| Order_Aminicenantales | Staphylococcaceae |
| Order_Anaerolineae | Staphylococcus |
| Order_Anaerolineae | Stappia |
| Order_Armatimonadales | Stenotrophobacter |
| Order_Bacillales | Stenotrophomonas |
| Order_bacterium_Ellin6529 | Steroidobacter |
| Order_Bacteroidales | Stomatobaculum |
| Order_Betaproteobacteriales | Streptobacillus |
| Order_Blastocatellia | Streptococcus |
| Order_Blastocatellia | Streptococcus |
| Order_Chitinophagales | Streptomyces |
| Order_Chlamydiales | Streptomycetaceae |
| Order_Chloroplast | Subdoligranulum |
| Order_Clostridia | Sutterella |
| Order_Clostridiales | Tabrizicola |
| Order_Coriobacteriales | Taibaiella |
| Order_Coriobacteriia | Tannerella |
| Order_Corynebacteriales | Tardiphaga |
| Order_Cytophagales | Tepidiphilus |
| Order_Dehalococcoidia | Tepidisphaera |
| Order_Deltaproteobacteria | Terribacillus |
| Order_Deltaproteobacteria | Terriglobus |
| Order_Deltaproteobacteria | Terrimicrobium |
| Order_Elsterales | Terrimonas |
| Order_Frankiales | Terrisporobacter |
| Order_Gaiellales | Tessaracoccus |
| Order_Gammaproteobacteria | Tetragenococcus |
| Order_Gammaproteobacteria | Thauera |
| Order_Gracilibacteria | Thermoactinomyces |
| Order_Gracilibacteria | Thermoanaerobaculaceae |
| Order_Hyaloperonospora_arabidopsidis | Thermobacillus |
| Order_Ignavibacteria | Thermomonas |
| Order_Izimaplasmatales | Thermus |
| Order_Kiritimatiellae | Tissierella |
| Order_Ktedonobacteria | Family_Saccharimonadaceae |
| Order_Lactobacillales | Tolypothrix |
| Order_Micavibrionales | Treponema |
| Order_Micrococcales | Trichococcus |
| Order_Microtrichales | Truepera |
| Order_Mollicutes_RF39 | Trueperella |
| Order_Myxococcales | Tsukamurella |
| Order_Nostocales | Tumebacillus |
| Order_Propionibacteriales | Turicella |
| Order_Pseudomonadales | Turicibacter |
| Order_Rhizobiales | Tychonema |
| Order_Rhodospirillales | Tyzzerella |
| Order_Rickettsiales | Ureaplasma |
| Order_Rokubacteriales | Ureibacillus |
| Order_Saccharimonadales | Vagococcus |
| Order_Solirubrobacterales | Varibaculum |
| Order_uncultivated | Variovorax |
| Order_Xanthomonadales | Veillonella |
| Oribacterium | Verticia |
| Ornithinicoccus | Vibrio |
| Ornithinimicrobium | Virgibacillus |
| Oscillatoria | Virgisporangium |
| Ottowia | Vogesella |
| Oxyphotobacteria_Incertae_Sedis | Vulcaniibacterium |
| Oxyphotobacteria_Incertae_Sedis | Weeksella |
| Paenarthrobacter | Weissella |
| Paenibacillus | Williamsia |
| Paeniclostridium | Wilmottia |
| Paeniglutamicibacter | Xanthomonas |
| Paenisporosarcina | Xylophilus |
| Pajaroellobacter | Yaniella |
| Paludibacteraceae | Yersinia |
| Paludibaculum | Zoogloea |
| Paludisphaera |  |
| Pannonibacter |  |
| Pantoea |  |
| Paraburkholderia_tropica |  |
| Paraclostridium |  |
| Paracoccus |  |
| Parafilimonas |  |
| Parapedobacter |  |
| Parasutterella |  |
| Parvibaculum |  |
| Parvimonas |  |
| Parviterribacter |  |
| Pasteurella |  |
| Patulibacter |  |
| Paucisalibacillus |  |
| Pectobacterium |  |
| Pedobacter |  |
| Pedomicrobium |  |
| Pelagibacterium |  |
| Pelosinus |  |
| Peptoclostridium |  |
| Peptococcus |  |
| Peptoniphilus |  |
| Peptostreptococcus |  |
| Peredibacter |  |
| Persicitalea |  |
| Phascolarctobacterium |  |
| Phaselicystis |  |
| Phenylobacterium |  |
| Phormidesmis |  |
| Phormidesmis |  |
| Phormidium |  |
| Phormidium |  |
| Photobacterium |  |
| Phreatobacter |  |
| Phycicoccus |  |
| Phycisphaeraceae |  |
| Phyllobacterium |  |
| Phylum_Actinobacteria |  |
| Phylum_Armatimonadetes |  |
| Phylum_BRC1 |  |
| Phylum_FBP |  |
| Phylum_Firmicutes |  |
| Phylum_Patescibacteria |  |
| Phylum_Proteobacteria |  |
| Phylum_WPS-2 |  |
| Pigmentiphaga |  |
| Pilimelia |  |
| Piscicoccus |  |
| Planctomicrobium |  |
| Planifilum |  |
| Planococcus |  |
| Planomicrobium |  |
| Plantibacter |  |
| Pleomorphomonas |  |
| Pleurocapsa |  |
| Polaromonas |  |
| Polyangium |  |
| Polymorphobacter |  |
| Pontibacter |  |
| Porphyrobacter |  |
| Porphyromonas |  |
| Portibacter |  |
| Prevotella |  |
| Procabacter |  |
| Promicromonospora |  |
| Propioniciclava |  |
| Propionicimonas |  |
| Propionimicrobium |  |
| Prosthecobacter |  |
| Proteiniborus |  |
| Proteiniclasticum |  |
| Providencia |  |
| Pseudactinotalea |  |
| Pseudarthrobacter |  |
| Pseudoalteromonas |  |
| Pseudoclavibacter |  |
| Pseudofulvimonas |  |
| Pseudoglutamicibacter |  |
| Pseudogracilibacillus |  |
| Pseudokineococcus |  |
| Pseudolabrys |  |
| Pseudomonas |  |
| Pseudonocardia |  |
| Pseudopropionibacterium |  |
| Pseudorhodoferax |  |
| Pseudosphingobacterium |  |
| Pseudoxanthomonas |  |
| Psychrobacillus |  |
| Psychrobacter |  |
| Psychroflexus |  |
| Psychroglaciecola |  |
| Pyrinomonadaceae |  |
| Qipengyuania |  |
| Quadrisphaera |  |
| Rahnella |  |
| Raineyella |  |
| Ramlibacter |  |
| Rathayibacter |  |
| Reyranella |  |
| Rheinheimera |  |
| Rhizobacter |  |
| Rhizorhapis |  |
| Rhodanobacter |  |
| Rhodobacter |  |
| Rhodococcus |  |
| Rhodocytophaga |  |
| Rhodoferax |  |
| Rhodomicrobium |  |
| Rhodopila |  |
| Rhodoplanes |  |
| Rhodopseudomonas |  |
| Rhodovarius |  |
| Rhodovastum |  |
| Rickettsia |  |
| Rickettsiella |  |
| Rikenellaceae |  |
| Risungbinella |  |
| Romboutsia |  |
| Roseburia |  |
| Roseiflexus |  |
| Roseivirga |  |
| Roseomonas |  |
| Roseovarius |  |
| Rothia |  |
| Rubellimicrobium |  |
| Rubinisphaeraceae |  |
| Rubritepida |  |
| Rubrivirga |  |
| Rubrobacter |  |
| Rudanella |  |
| Rufibacter |  |
| Ruminiclostridium |  |
| Ruminococcaceae |  |
| Ruminococcaceae |  |
| Ruminococcaceae |  |
| Ruminococcaceae |  |
| Ruminococcaceae |  |
| Ruminococcaceae |  |
| Ruminococcaceae |  |
| Ruminococcaceae |  |
| Ruminococcus |  |
| Ruminofilibacter |  |
| Rummeliibacillus |  |
| Saccharibacillus |  |
| Saccharopolyspora |  |
| Salana |  |
| Salegentibacter |  |
| Salinicoccus |  |
| Salinicola |  |
| Salinimicrobium |  |
| Salinispora |  |
| Salinivibrio |  |
| Sandaracinobacter |  |
| Sandaracinus |  |
| Sanguibacter |  |
| Sarcina |  |
| Scardovia |  |
| Schlegelella |  |
| Scytonema |  |
| Scytonema |  |
| Sedimentibacter |  |
| Sediminibacterium |  |
| Sediminivirga |  |
| Segetibacter |  |
| Selenomonas |  |
| Sellimonas |  |
| Serinicoccus |  |
| Serratia |  |
| Shewanella |  |
| Shimwellia |  |
| Shinella |  |
| Shuttleworthia |  |
| Silvanigrella |  |
| Simonsiella |  |
| Singulisphaera |  |
| Sinobaca |  |
| Siphonobacter |  |
| Skermanella |  |
| Slackia |  |
| Sneathia |  |
| Solibacillus |  |
| Solirubrobacter |  |
| Solirubrobacteraceae |  |
| Solobacterium |  |
| Sorangium |  |
| Spelaeicoccus |  |
| Sphingoaurantiacus |  |
| Sphingobacterium |  |
| Sphingobium |  |
| Sphingomonadaceae |  |
| Sphingomonadaceae |  |
| Sphingomonadaceae |  |
| Sphingomonas |  |
| Sphingopyxis |  |
| Sphingorhabdus |  |
| Spiroplasma |  |
| Spirosoma |  |
| Sporobacter |  |
| Sporocytophaga |  |
| Sporolactobacillus |  |
| Sporosarcina |  |
| Staphylococcaceae |  |
| Staphylococcus |  |
| Stenotrophobacter |  |
| Stenotrophomonas |  |
| Steroidobacter |  |
| Stigonema |  |
| Stomatobaculum |  |
| Streptobacillus |  |
| Streptococcus |  |
| Streptococcus |  |
| Streptomonospora |  |
| Streptomyces |  |
| Streptomycetaceae |  |
| Streptosporangium |  |
| Subdoligranulum |  |
| Subsaxibacter |  |
| Succiniclasticum |  |
| Sutterella |  |
| Symbiobacterium |  |
| Symplocastrum |  |
| Syntrophaceticus |  |
| Tabrizicola |  |
| Taibaiella |  |
| Tannerella |  |
| Tardiphaga |  |
| Tepidimicrobium |  |
| Tepidimonas |  |
| Tepidiphilus |  |
| Tepidisphaera |  |
| Terribacillus |  |
| Terriglobus |  |
| Terrimicrobium |  |
| Terrimonas |  |
| Terrisporobacter |  |
| Tessaracoccus |  |
| Tetrasphaera |  |
| Thauera |  |
| Thermacetogenium |  |
| Thermaerobacter |  |
| Thermoactinomyces |  |
| Thermoanaerobacterium |  |
| Thermoanaerobaculaceae |  |
| Thermobacillus |  |
| Thermobifida |  |
| Thermobispora |  |
| Thermoflavimicrobium |  |
| Thermomonas |  |
| Thermus |  |
| Tissierella |  |
| Family_Saccharimonadaceae |  |
| Family_Saccharimonadaceae |  |
| Family_Saccharimonadaceae |  |
| Tolypothrix |  |
| Tomitella |  |
| Treponema |  |
| Truepera |  |
| Tuberibacillus |  |
| Tumebacillus |  |
| Tunicatimonas |  |
| Turicella |  |
| Turicibacter |  |
| Tychonema |  |
| Tyzzerella |  |
| Ulvibacter |  |
| unc_Caldilinea |  |
| Ureaplasma |  |
| Ureibacillus |  |
| Vagococcus |  |
| Varibaculum |  |
| Variovorax |  |
| Veillonella |  |
| Verticia |  |
| Vibrio |  |
| Virgibacillus |  |
| Virgisporangium |  |
| Viridibacillus |  |
| Vulcaniibacterium |  |
| wb1_P19 |  |
| Weeksella |  |
| Weissella |  |
| Williamsia |  |
| Wilmottia |  |
| Wolbachia |  |
| Wolinella |  |
| Xanthomonas |  |
| Xylophilus |  |
| Yaniella |  |
| Yersinia |  |
| Youngiibacter |  |

**Table S14.** The effects of season and environmental factors on bed dust alpha and beta-diversity. ^Alpha diversity were calculated based on observed richness and significance were calculated using the Wilcoxon test (for two groups) and Kruskal-Wallis test (for three or more groups), FDR corrected. #Effects were quantified with R2, and p-values, as determined by PERMANOVA on weighted UniFrac distances. Significant adjusted p-values (p<0.05) are shown in bold. Abbreviations - R: Rural, U: Urban, H: House, A: Apartment, C: Cat, D: Dog, B: both cat and dog.

| Season | Category | Variables | n | Bacterial  Alpha Diversity  (median richness) | Bacterial  Alpha Diversity^  Adjusted p-value | Bacterial  Alpha Diversity  Order | Bacterial  Beta  Diversity#  R2 / p-value | Fungal  Alpha Diversity  (median richness) | Fungal  Alpha Diversity^  Adjusted p-value | Fungal  Alpha Diversity Order | Fungal  Beta  Diversity#  R2 / p-value |
| --- | --- | --- | --- | --- | --- | --- | --- | --- | --- | --- | --- |
| Summer | Living Environment | Rural | 65 | 293 | 0.21 | R>U | 0.0077/0.302 | 270 | 0.91 | R>U | 0.0081/0.28 |
|  |  | Urban | 75 | 272 |  |  |  | 261 |  |  |  |
| Fall |  | Rural | 60 | 296 | 0.079 | R>U | 0.005/0.626 | 243 | 0.34 | R>U | 0.0065/0.375 |
|  |  | Urban | 101 | 269 |  |  |  | 216 |  |  |  |
| Winter |  | Rural | 67 | 296 | **0.012** | R>U | 0.01/0.09 | 236 | 0.23 | R>U | 0.00567/0.51 |
|  |  | Urban | 67 | 258 |  |  |  | 220 |  |  |  |
| Spring |  | Rural | 55 | 299 | 0.32 | R>U | 0.013/0.087 | 266 | 0.93 | R>U | 0.0413/**0.015** |
|  |  | Urban | 52 | 281 |  |  |  | 263 |  |  |  |
| Summer | Type of Home | Apartment | 60 | 274 | 0.35 | H>A | 0.0068/0.485 | 240 | 0.067 | H>A | 0.0156/**0.046** |
|  |  | House | 81 | 290 |  |  |  | 273 |  |  |  |
| Fall |  | Apartment | 77 | 266 | 0.19 | H>A | 0.005/0.432 | 222 | 0.11 | H>A | 0.024/**0.002** |
|  |  | House | 88 | 292 |  |  |  | 237 |  |  |  |
| Winter |  | Apartment | 57 | 264 | 0.16 | H>A | 0.008/0.362 | 205 | 0.099 | H>A | 0.008/0.362 |
|  |  | House | 77 | 292 |  |  |  | 236 |  |  |  |
| Spring |  | Apartment | 37 | 248 | 0.13 | H>A | 0.0146/**0.046** | 253 | 0.28 | H>A | 0.0041/0.708 |
|  |  | House | 70 | 301 |  |  |  | 268 |  |  |  |
| Summer | Pet Groups | Cat | 14 | 325 | 0.62 | C>D>B | 0.023/0.218 | 294 | 0.52 | C>D>B | 0.03/0.085 |
|  |  | Dog | 18 | 303 |  |  |  | 267 |  |  |  |
|  |  | Both | 9 | 290 |  |  |  | 246 |  |  |  |
| Fall |  | Cat | 26 | 281 | **0.003** | B>C>D | 0.024/**0.045** | 261 | **0.0061** | C>B>D | 0.0141/0.771 |
|  |  | Dog | 18 | 264 |  |  |  | 206 |  |  |  |
|  |  | Both | 5 | 552 |  |  |  | 242 |  |  |  |
| Winter |  | Cat | 24 | 219 | **0.0023** | B>D>C | 0.02/0.533 | 235 | 0.29 | B>C>D | 0.022/0.381 |
|  |  | Dog | 20 | 273 |  |  |  | 230 |  |  |  |
|  |  | Both | 9 | 347 |  |  |  | 253 |  |  |  |
| Spring |  | Cat | 21 | 307 | 0.59 | D>C>B | 0.028/0.313 | 212 | 0.69 | B>D>C | 0.039/0.168 |
|  |  | Dog | 12 | 372 |  |  |  | 298 |  |  |  |
|  |  | Both | 8 | 271 |  |  |  | 313 |  |  |  |

**Table S15.** The adjusted (marginal) effects of environmental factors on bed dust alpha and beta-diversity. ^Alpha diversity were calculated based on observed richness and significance were calculated using the ANOVA. #Effects were quantified with R2, and p-values, as determined by PERMANOVA on weighted UniFrac distances. Significant adjusted p-values (p<0.05) are shown in bold.

|  | Living Environment | Type of home | Pets | Season | Siblings |
| --- | --- | --- | --- | --- | --- |
| Bacterial (alpha)^  (p-value) | **0.0043** | 0.8812 | **0.0069** | **0.0388** | 0.253 |
| Fungal (alpha)^  (p-value) | 0.1525 | **0.0032** | 0.5739 | **6.17e-05** | **0.0082** |
| Bacterial (beta)#  (R2/p-value) | **0.003/0.022** | 0.002/0.24 | **0.007/0.048** | **0.021/0.001** | **0.01/0.01** |
| Fungal (beta)#  (R2/p-value) | **0.009/0.001** | 0.001/0.3 | 0.005/0.3 | **0.084/0.001** | 0.01/0.08 |
